# A temperature-responsive regulator that enhances virulence in the kiwifruit canker pathogen *Pseudomonas syringae* pv. a*ctinidiae*

**DOI:** 10.1016/j.csbj.2025.05.017

**Published:** 2025-05-15

**Authors:** Xueting He, Yifei Zhang, Chenbei Xu, Kaidi Fu, Yiqing Ding, Tiantian Zhang, Tingtao Chen, Aprodisia Murero, Limin Wang, Yuan Xu, Cheng Chen, Jinghui Yang, Li Li, Caihong Zhong, Lili Huang, Xin Deng, Xiaolong Shao, Guoliang Qian

**Affiliations:** a College of Plant Protection (State Key Laboratory of Agricultural and Forestry Biosecurity, College of Plant Protection), Nanjing Agricultural University, Nanjing 210095, China; b Institute of Fruit Tree Research, Guangdong Academy of Agricultural Sciences；Key Laboratory of South Subtropical Fruit Biology and Genetic Resource Utilization, Ministry of Agriculture and Rural Affairs；Guangdong Provincial Key Laboratory of Science and Technology Research on Fruit Tree, Guangzhou 510640, China; c Jiangsu Hilly Area Zhenjiang Institute of Agricultural Sciences, Jiangsu Academy of Agricultural Sciences, Jurong 212400, China; d CAS Engineering Laboratory for Kiwifruit Industrial Technology, CAS Key Laboratory of Plant Germplasm Enhancement and Specialty Agriculture, Wuhan Botanical Garden, Chinese Academy of Sciences, Wuhan, Hubei Province 430074, China; e State Key Laboratory of Crop Stress Biology for Arid Areas, College of Plant Protection, Northwest A&F University, Yangling, Shaanxi 712100, China; f Department of Biomedical Sciences, City University of Hong Kong, Kowloon Tong, Hong Kong SAR, China; g Shenzhen Research Institute, City University of Hong Kong, Shenzhen, Guangdong 518057, China

**Keywords:** Kiwifruit canker disease, *Pseudomonas syringae* pv. *actinidae*, Low temperature, Heat shock protein, HrpL, Type III secretion system

## Abstract

*Pseudomonas syringae* pv. *actinidiae* (*Psa*), the causative agent of kiwifruit canker disease, poses significant threats to global kiwifruit production, resulting in substantial economic losses. Disease incidence is notably higher under cooler temperatures (<20℃), yet the molecular mechanisms underlying *Psa*'s temperature-dependent virulence remain poorly understood. Here, we identify RS16350, encoding a heat shock protein homolog, as a positive regulator of *Psa* pathogenicity specifically at low temperature (16℃) but not at optimal growth temperature (28℃). Mechanistic studies reveal that RS16350 physically interacts with HrpL, the RpoN-dependent sigma factor controlling type III secretion system (T3SS) expression in *Psa*. This interaction enhances HrpL's binding affinity to the *hrp-box* promoter element, thereby upregulating T3SS effector genes and increasing virulence. We designate this novel regulator as TrpR2 (temperature-responsive pathogenic regulator 2). These findings provide molecular insights into temperature-modulated virulence in a key plant pathogen and identify potential targets for developing innovative disease control strategies.

## Graphical Abstract

**Figure:**
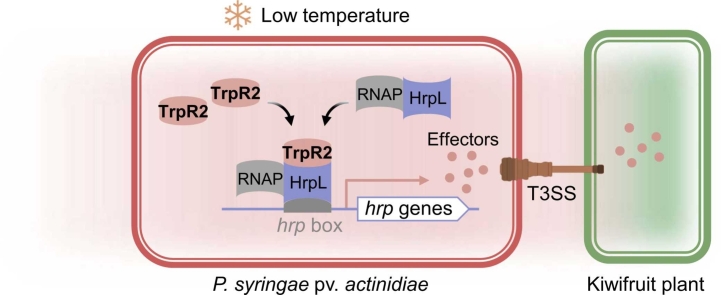

## 1 Introduction

Temperature plays a pivotal role in regulating bacterial enzymatic activity, membrane-associated functions, and gene expression [33], [36]. Fluctuations in temperature alter gene expression by activating temperature-sensing mechanisms that detect environmental changes and trigger appropriate responses [39], [42]. In plant pathogenic bacteria such as *Pseudomonas syringae*, temperature shifts modulate virulence gene expression through diverse regulatory systems [4], [6], [19]. These virulence factors facilitate host colonization through processes including phytotoxin biosynthesis, ice nucleation, exopolysaccharide production, and type III secretion system (T3SS)-mediated effector delivery [40].

The molecular basis of temperature-dependent virulence regulation in plant pathogens primarily involves conformational changes in DNA, RNA, or protein conformation, as well as membrane remodeling [17], [18], [20], [24], [26], [34]. However, the mechanisms underlying temperature effects on virulence determinants remain poorly understood. Notably, most virulence genes in plant pathogenic bacteria exhibit altered transcription levels at suboptimal temperatures compared to their expression under optimal growth conditions [17], [40]. To date, only 5 few thermosensing mechanisms linked to virulence gene regulation have been characterized in these bacteria, primarily involving two-component systems. Examples include the PhoP-PhoQ system in *Edwardsiella tarda*, which coordinates temperature-dependent virulence, and the CorSR system in *Pseudomonas syringae*, which enhances phytotoxin production [31], [41], [44], [5].

Bacterial canker disease, caused by *Pseudomonas syringae* pv. *actinidae* (*Psa*), is one of the most significant diseases affecting the kiwifruit industry on a global scale [16], [8]. *Psa* primarily targets its host plants through the T3SS, infecting the branches, leaves, and flowers via stomata and wounds [13], [50]. From the perspective of kiwifruit hosts, low temperature (16℃) significantly reduced the ethylene-mediated defense responses of kiwifruit to *Psa*, since low temperature inhibited the ethylene biosynthesis and signaling pathway of kiwifruit hosts, which in turn led to higher susceptibility of kiwifruit to *Psa* [47]*.* From the pathogen’s perspective, although temperature of 22–28℃ is optimal for growth and reproduction of *Psa*, increasing temperature combined with decreased leaf humidity prevent the formation and progression of symptoms [1], [9]. When the average daily temperature exceeds 22℃ and the leaf wetness (70 %-80 % humidity threshold) is fewer than 4 hours per day for more than 3 days, the disease undergoes an incubation period that overlaps with summer under Italian conditions [9]. A recent study shows that *Psa* moves more swiftly within kiwifruit veins at 16℃ than at 25℃, accelerating disease progression [7], [13]. However, it is unclear how the low temperature and cold environment affect the pathogenicity of *Psa*.

Heat shock proteins (HSPs) serve as pivotal virulence regulators across bacterial species, with well-characterized mechanisms in *Pseudomonas aeruginosa* and *Pseudomonas cichorii* [33], [43], [55]. During early plant infection phases, pathogenic bacteria significantly upregulate HSP production, particularly when ambient temperatures exceed their optimal growth range by 8–12℃. Functioning as evolutionarily conserved molecular chaperones, these proteins orchestrate essential biological processes including: protein folding homeostasis, stressor-induced cytoprotection, immunomodulation, and cellular signaling regulation [12].

Notably, comparative studies in model organisms (*Escherichia coli*, *Bacillus subtilis*, *Streptomyces spp.*) reveal expanded functional repertoires of HSPs beyond canonical proteostatic roles [11]. These molecular chaperones demonstrate pleiotropic stress-adaptive functions, mediating: thermal resilience through protein disaggregation, hypoxia tolerance via redox balance maintenance, UV radiation resistance by DNA repair facilitation, and cytoskeletal reorganization during environmental adaptation [29]. Additionally, HSPs have been found to influence the virulence of fungal and bacterial pathogens [12], [15], [29], [53], [54]. For instance, DnaJ, a member of the heat shock protein 40 family, regulates the survival and virulence of the plant pathogen *P. cichorii* [43].

This investigation establishes a temperature-responsive virulence mechanism in *Pseudomonas syringae* pv. *actinidiae* through functional characterization of TrpR2—a thermosensitive transcriptional co-activator encoded by a small heat shock protein (sHSP) gene. Biochemical and genetic analyses demonstrate that TrpR2 directly interacts with HrpL, the central sigma factor governing Type III Secretion System (T3SS) transcription, to enhance RNA polymerase binding affinity at *hrp-box-*containing promoters. This molecular partnership drives cold-adapted pathogenicity by coordinately upregulating T3SS effector genes during low-temperature (16℃) infection. Our findings resolve a paradigm wherein phytopathogenic bacteria co-opt molecular chaperones for environmental sensing, revealing functional divergence of HSPs as transcriptional amplifiers in thermal regulation of virulence pathways.

## 2 Results

### 2.1 Identification of *Psa* regulons under low-temperature conditions

Kiwifruit canker disease is known to predominantly occur under cold and wet conditions [2], [14]. To examine the effect of low temperature on disease development, we performed transcriptomic analysis of *Psa* cultured in T3SS-inducing minimal medium (MM) at two temperatures: 16℃ (lower temperature) and 28℃ (higher temperature), to mimic season related temperature changes.

**Fig. 1:**
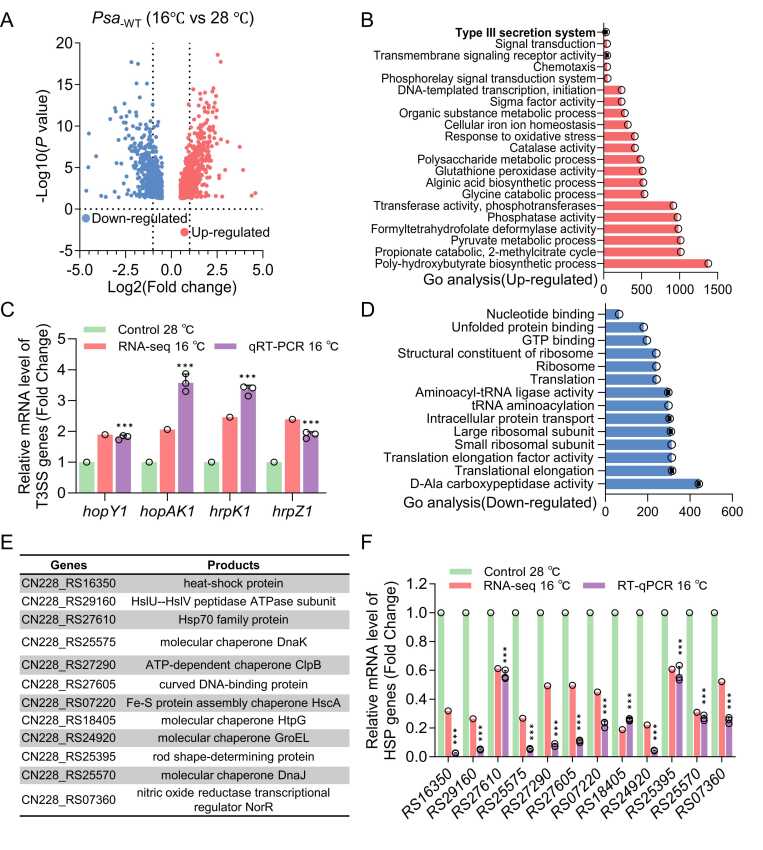
**Transcriptomic analysis reveals*****Psa’s*****low temperature regulon.** (A) Volcano plot of differential expressed genes in *Psa* at 16℃ versus 28℃. (B) Gene Ontology (GO) enrichment analysis of upregulated genes. (C) Validation of T3SS gene expression (*hopY1*, *hopAK1*, *hrpZ1*, and *hrpK1)* by RNA-seq and qRT-PCR, showing significantly higher expression at 16℃. (D) GO enrichment analysis of downregulated genes. (E) Annotation of 12 significantly downregulated heat shock proteins (HSPs) at 16℃. (F) RNA-seq and qRT-PCR confirmation of HSPs downregulation at 16℃. Data represent mean ± SD (n ≥ 3). * ** P < 0.001.

Our transcriptome data revealed significant differential expressed genes (DEGs), with 708 genes downregulated and 741 genes upregulated at 16℃ compared to 28℃ (Fig. 1A; Table S1). Gene Ontology (GO) enrichment analysis demonstrated that upregulated genes were predominantly associated with T3SS function, biosynthetic processes, and metabolic pathways (Figs. 1B, 1C). This temperature-dependent upregulation of T3SS genes (including *hopY1*, *hopAK1*, *hrpZ1*, and *hrpK1*) were further confirmed by quantitative reverse transcription PCR (qRT-PCR) (Fig. 1C).

Conversely, downregulated genes were enriched in functional categories including carboxypeptidase activity, translational elongation, ribosome biogenesis, and nucleotide binding (Fig. 1D). Notably, we observed significant downregulation of 12 HSPs genes (listed in Fig. 1E), which was validated by qRT-PCR (Fig. 1F). This finding suggests that HSPs serve as temperature sensors in *Psa*. To systematically investigate the potential correlation between HSPs, T3SS regulation, and pathogenicity at low temperature, we analyzed all 23 HSP genes in the *Psa* genome. As detailed in Fig. S1A, our experimental approach involves: (1) screening for temperature-responsive HSPs through pathogenicity assays on kiwifruit leaves at 16℃, followed by (2) evaluation of their effects on T3SS genes expression.

### 2.2 The predicted HSP RS16350 (TrpR2) positively regulates the *Psa* pathogenicity under low-temperature conditions

**Fig. 2:**
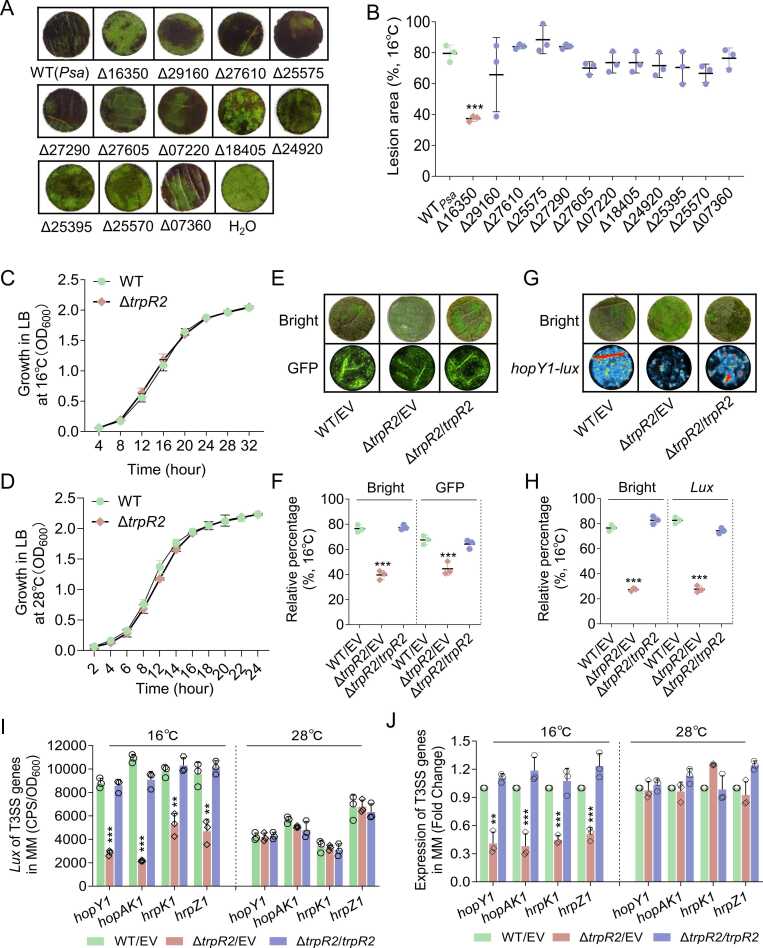
**RS16350 is essential for*****Psa*****pathogenicity and T3SS activation at low temperature.** (A) Leaf disc assays showing reduced pathogenicity of ΔRS16350 compared to wild-type (WT). Lesion areas were quantified using ImageJ (average of 10 discs per replicate). (B) Representative images of lesion development, indicating bacterial spread in host tissue. (C-D) Growth curve of ΔRS16350 and wild type in LB medium at 16℃ and 28℃. (E-F) GFP fluorescence intensity quantification of infected leaf discs (ImageJ analysis of 3 discs per replicate). (G-H) *hopY1-lux* reporter activity in leaf discs. (I) *Lux*-reporter assays of T3SS gene expression (*hopY1*, *hopAK1*, *hrpK1*, *hrpZ1*) in Δ*trpR2*, complemented strain (Δ*trpR2*/*trpR2*), and WT at both temperatures. (J) qRT-PCR validation of T3SS genes expression. Data represent mean ± SD (n ≥ 3). * *P < 0.01, * **P < 0.001.

To investigate the role of HSPs in *Psa’s* temperature response, we constructed a library of 10 in-frame HSP deletion mutants in the wild-type *Psa* M228 strain. Pathogenicity assay on kiwifruit leaves revealed that the ΔRS16350 mutant showed significantly reduced virulence compared to wild-type M228 strain. Quantitative analysis of lesion areas confirmed this attenuation, particularly at 16℃ (Figs. 2A, 2B). Importantly, RS16350 deletion did not affect bacterial growth at either 16℃ or 28℃ (Figs. 2C, 2D), prompting us to designate this gene as TrpR2 (temperature-responsive pathogenic regulator 2).

To characterize TrpR2’s function, we generated a complemented strain (∆*trpR2*/*trpR2*) by introducing a TrpR2 overexpression plasmid into ∆*trpR2* mutant. Using GFP-labeled *Psa* strains to quantify viable bacteria *in planta*, we observed that both lesion development (Fig. 2E) and bacterial fluorescence intensity (Fig. 2F) were fully restored in the complemented strain. These results demonstrate that HSP family member, TrpR2, has a specific role in regulating temperature-dependent virulence in *Psa*.

### 2.3 TrpR2 enhances T3SS gene expression under low-temperature conditions

Given the central role of T3SS in *Psa*, we investigated the relationship between TrpR2 and T3SS regulation. Using *hopY1* (encoding the type III effector HopY1) as the T3SS reporter, we constructed a *hopY1-lux* transcriptional fusion and introduced it into wild-type (WT/EV), ∆*trpR2*/EV, and complemented ∆*trpR2*/*trpR2* stains. *Lux*-reporter assays in infected kiwifruit leaves revealed significantly reduced luminescence in Δ*trpR2* compared to both wild-type and complemented strains (Figs. 2G, 2H), demonstrating TrpR2's positive regulation of T3SS expression.

To verify this temperature-dependent regulation, we examined additional T3SS genes, (*hopAK1*, *hrpK1*, and *hrpZ1*) in minimal medium at both 16℃ and 28℃ conditions. Both *lux*-reporter assays and qRT-PCR analyses consistently showed TrpR2-dependent activation of these genes at 16℃ (Figs. 2I, 2J), confirming that TrpR2 modulates temperature-responsive pathogenicity through T3SS regulation.

### 2.4 Identification of functional residues in TrpR2

**Fig. 3:**
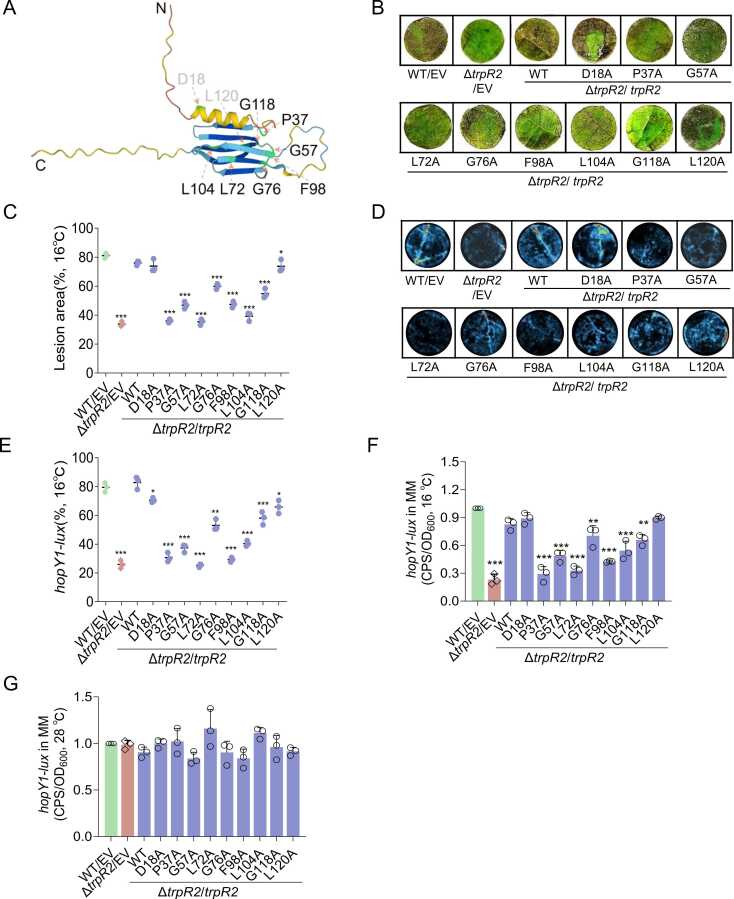
**Functional characterization of conserved residues in TrpR2.** (A) Predicted 3D structure of TrpR2 (AlphaFold) with nine conserved residues indicated. (B-C) Pathogenicity assays of alanine substitution mutants, with lesion areas quantified (3 discs per replicate). (D-E) *hopY1*-*lux* expression in mutants at 16℃ (*in planta)*. (F-G) *hopY1-lux* expression at both temperatures in T3SS-inducing medium. Data represent mean ± SD (n ≥ 3). * **P < 0.001.

Bioinformatic analysis revealed that TrpR2 contains conserved HSP domains and is widely distributed among bacterial species, with particularly high conservation in the *Pseudomonas* complex (Fig. 3A, S2A). Sequence alignment identified nine highly conserved residues (identity >75 %): Asp18, Pro37, Gly57, Leu72, Gly76, Phe98, Leu104, Gly118, and Leu120 (Fig. S2B).

We generated alanine (A) substitution mutants for each residue and assessed their functional impact. Pathogenicity assays showed that all nine residues were essential for TrpR2-mediated virulence *in planta* (Figs. 3B, 3C). Subsequent *lux*-reporter assays at both temperatures confirmed that all of these variants failed to restore *hopY1* expression either *in planta* (Figs. 3D, 3E) or T3SS-inducing minimal medium (Figs. 3F, 3G), highlighting their critical role in TrpR2 function.

### 2.5 TrpR2 binds to the T3SS regulator HrpL to enhance T3SS gene expression

**Fig. 4:**
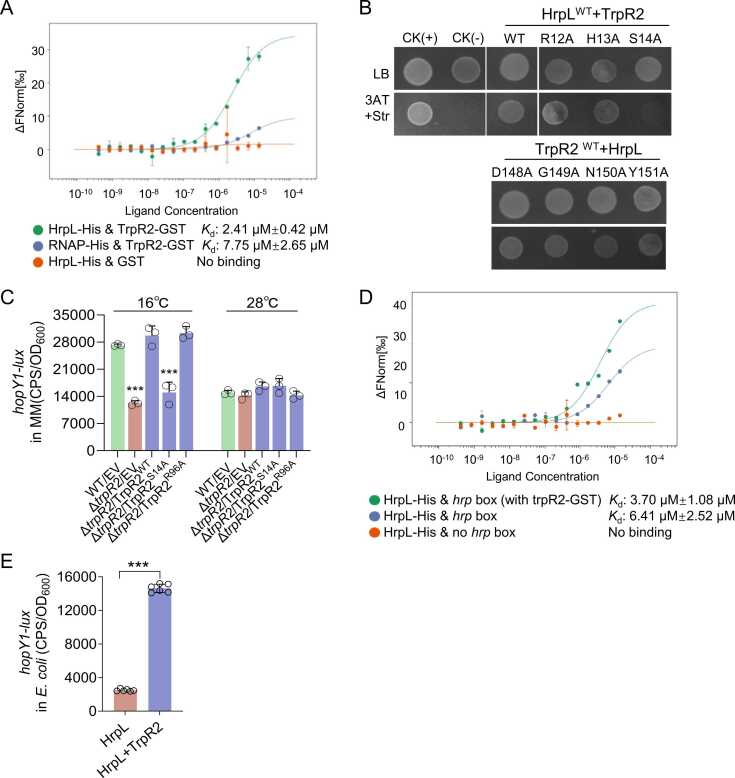
**TrpR2-mediated T3SS regulation via HrpL interaction.** (A) MST analysis confirming TrpR2-HrpL interaction (*K*_d_ = 2.41 μM). (B) Bacterial two-hybrid mapping of interaction candidate residues between TrpR2 and HrpL. (C) *hopY1-lux* expression in TrpR2 point mutants (TrpR2^S14A^ and TrpR2^R96A^). (D) MST showing TrpR2-enhanced HrpL binding to *hrp-box*-containing promoter (*K*_d_ = 3.70 μM vs 6.41 μM without TrpR2). (E) HrpL-dependent *hopY1* expression in *E. coli*, enhanced 5-fold by TrpR2 co-expression. Data represent mean ± SD (n ≥ 3). * **P < 0.001.

Given that TrpR2 lacks identifiable DNA-binding domain (Fig. 3A) and T3SS gene expression is controlled by the HrpR/HrpS-HrpL regulatory pathway, we hypothesized it regulates T3SS through interaction with known T3SS regulators. To test our hypothesis, we performed a bacterial two-hybrid assay, and results revealed specific interaction between TrpR2 and HrpL, but not with HrpR or HrpS (Fig. S3A). This interaction was confirmed by microscale thermophoresis (MST), showing strong binding between TrpR2-GST and HrpL-His (*K*_d_ =2.41 μM), significantly higher than control interactions (Fig. 4A). Since we were unable to obtain soluble HrpL, we co-expressed and purified HrpL with RNAP as previously described [46].

While the nine conserved residues identified in Fig. S2B were not critical for TrpR2-HrpL binding (Fig. S3B), molecular docking was employed to predict potential interaction sites (Fig. S4-S5). Notably, Arg96 (key residue), Arg12-His13-Ser14 cluster, and other surface residues are potential interaction sites for TrpR2. Ser144-Asp148-Gly149-Asn150-Tyr151 region are potential interaction sites for HrpL. Site-directed mutagenesis and bacterial two-hybrid assays were respectively performed to identify these predicted sites. Firstly, the TrpR2 R12-S14 region and HrpL D148-Y151 region are important for interaction. Then, TrpR2 Ser14 and HrpL Asn150 are further identified as key contact residues (Fig. 4B; S6B-S6C). Functional validation showed TrpR2^S14A^ failed to restore *hopY1*-*lux* expression in ΔTrpR2, while TrpR2^R96A^ (negative control) fully complemented this defect (Fig. 4C), confirming Ser14's essential role.

### 2.6 Mechanistic basis of TrpR2-mediated T3SS regulation

Based on these findings, we hypothesized that TrpR2 enhances expression of HrpL-dependent T3SS genes containing the *hrp-*box sequence. To investigate TrpR2-HrpL interaction, we first conducted microscale thermophoresis (MST) experiments to determine whether TrpR2 enhances the binding affinity of HrpL to *hrp*-box. As depicted in Fig. 4E, HrpL specifically binds *hrp*-box-containing *hopY1* promoter (*K*_d_=6.41 μM), while no binding occurred between HrpL and the *hopY1* promoter without *hrp*-box. When TrpR2 was introduced, the binding affinity between HrpL and *hrp*-box was significantly enhanced (*K*_d_=3.70 μM), indicating that the TrpR2-HrpL complex promotes the binding of HrpL to *hrp*-box (Fig. 4D).

To further investigate the effect of the TrpR2-HrpL complex on the expression of *hrp* genes, transcriptional reporter assays in *E. coli* showed co-expression of TrpR2 and HrpL increased *hopY1-lux* expression ∼5-fold compared to HrpL alone (Fig. 4E). Our findings confirm that TrpR2-HrpL interaction strengthens HrpL's binding to *hrp-*box, and this enhanced binding boosts transcription of downstream T3SS genes.

## 3 Discussion

The phytopathogen *Pseudomonas syringae* pv. *actinidiae* (*Psa*), the causative agent of kiwifruit canker disease, exhibits enhanced environmental adaptability and infectivity under low-temperature conditions [32], [47], though the molecular basis of its temperature-modulated pathogenesis remains elusive. Our study identifies TrpR2, encoding a heat shock protein (HSP), as a thermosensitive virulence regulator that potentiates *Psa* pathogenicity by activating type III secretion system (T3SS) genes at lower temperatures. While the *Escherichia coli* homolog IbpA primarily functions as an aggregation sensor in heat shock response [27], [28], we demonstrate through protein interaction assays that TrpR2 directly chaperones HrpL—the master sigma factor governing T3SS transcription—in plant pathogens.

We propose a temperature-dependent regulatory model: at 16℃, TrpR2-HrpL complex formation enhances RNA polymerase binding to *hrp-box*-containing promoters, driving T3SS activation and virulence potentiation. This regulatory axis becomes functionally compromised at 28℃, suggesting a thermal switch mechanism. These findings establish a novel cold-adaptation pathway where bacterial pathogens co-opt molecular chaperones for environmental sensing. TrpR2 may serve as a promising and innovative candidate target for molecular intervention against kiwifruit canker.

The expression of virulence genes in plant-pathogenic bacteria has been well documented in *Pseudomonas syringae*, *Pectobacterium spp.*, and *Dickeya dadantii* and others [4], [6], [17], [19], [20]. While temperature-modulated virulence mechanisms have been characterized in various phytopathogens—including the CorSR-phytotoxin pathway in *P. syringae* [31], [41], [44]—the thermal regulation of T3SS core components remains poorly understood. Previous studies reported temperature-dependent secretion patterns for specific effectors like HopPsyA and AvrPto [45], yet failed to identify upstream regulators coordinating this response. Genomic localization analysis demonstrated that 80 % of the temperature-responsive effectors (*hopY1*, *hopF1*, *hopAF1*, *avrB4–1*) and the helper protein HopAK1 reside outside the primary T3SS genomic island. This spatial distribution pattern suggests an expanded regulatory network governing cold-adapted virulence. Absence of canonical effectors like *hopM* and *avrE1* in our dataset likely stems from: i) Technical constraints in RNA-seq library preparation (rRNA depletion efficiency). ii) Sequencing depth limitations (30 M reads/sample) for low-abundance transcripts. We will prioritize targeted qRT-PCR validation of these key effectors in future experiments. Our work bridges this knowledge gap by elucidating TrpR2-mediated transcriptional control of T3SS genes under cold stress.

Heat shock proteins (HSPs) represent a broad category of chaperones present in both eukaryotic and prokaryotic organisms. Beyond their canonical roles in stress response, HSPs increasingly emerge as multifunctional regulators of bacterial virulence [10], [22], [25], [3]. In fact, HSPs have been found to have additional biological functions, such as regulating cytoskeleton kinetics, macrophage function, bacterial survival, biofilm formation, and virulence regulation[11], [15], [23], [29], [43], [53], [54], [55]. Our findings reveal an unprecedented mechanism where TrpR2, a predicted small HSP, undergoes cold-induced structural modifications that enhance its interaction with HrpL. This regulatory axis ultimately increases RNA polymerase recruitment to T3SS promoters, establishing a direct link between thermal sensing and virulence gene expression. To our knowledge, this represents the first demonstration of an HSP functioning as a thermosensitive transcriptional co-activator in bacterial pathogenesis. Additionally, our finding determined that the expression of *trpR2* is downregulated at lower temperatures, which is inconsistent with the activation of T3SS genes by TrpR2 at lower temperatures. Here, we propose that, although the transcription of *trpR2* is downregulated at lower temperatures, the TrpR2 protein produced from the reduced expression of *trpR2* remains effective in activating T3SS gene expression. Therefore, TrpR2 is required for full virulence. Moreover, our comparative RNA-seq data show that transcription of a well-characterized virulence regulator, AefR (CN228_RS11030; [21]), was also downregulated at lower temperatures (> 2-fold change; Table S1), which supports our hypothesis.

## 4 Materials and methods

### 4.1 Bacterial strains, plasmids, and growth conditions

Bacterial strains and plasmids used in this study have been listed in supplemental Table S2. The *Pseudomonas syringae* pv. *actinidiae* Shaanxi:M228 strain (*Psa*_M228) was cultured overnight at 28 ℃ in Luria-Bertani (LB) medium with shaking at 220 rpm and the *Escherichia coli* was grown at 37℃. Antibiotics were used for *E. coli* at the following concentrations: 50 μg/mL kanamycin (Km), 50 μg/mL spectinomycin (Spe), 34 μg/mL chloromycetin (Cm), 12.5 μg/mL tetracycline (Tc). Antibiotics were used for *Psa*_M228 at the following concentrations: 100 μg/mL kanamycin (Km), 100 μg/mL spectinomycin (Spe).

### 4.2 Transcriptome sequencing

RNA samples preparation and transcriptome sequencing was performed as previous described [37]. The *Psa*_M228 wild type strain was inoculated into Luria-Bertani (LB) broth with overnight cultured at 28℃. The strain was washed with MM [50 mM KH_2_PO_4_, 7. 6 mM (NH_4_)_2_SO_4_, 1. 7 mM MgCl_2_, 1. 7 mM NaCl, and 10 mM fructose, pH 5. 7] at least three times and diluted to OD_600_∼0.6. The bacterial samples were divided into duplicates and subjected to two temperatures (16℃ and 28℃) in MM cultures shaking for 7 h. Then, 2 mL of bacterial cultures were collected by centrifugation (12,000 rpm, 4℃). RNA extraction was performed by using the TianGen RNA extraction kit with the recommended DNase digestion. After removing rRNA by following the RiboRid protocol, mRNA was used to construct the cDNA library according to the NEBNext UltraTM II RNA Library Prep Kit protocol (NEB), which was then sequenced using the HiSeq 2000 system (Illumina). The sequencing depth (average coverage of 100-fold) and quality thresholds (Q30 ≥ 94 %) used for raw data processing.

### 4.3 Transcriptome analysis

Transcriptome analysis was performed as previous described [37]. Bacterial RNA-seq reads were trimed using Trimmomatic (v0.39, sliding-window 4:20), and mapped to the *Pseudomonas syringae* pv. *actinidiae* Shaanxi:M228 genome (GenBank accession: GCF_000344475.3) using STAR (v2.7.10a). The genome index was generated with the annotated GTF file and FASTA sequence, permitting a maximum splice alignment length of 30 kb. In paired-end mode, reads were aligned with parameters set as a maximum of 2 mismatches per read and a seed search length of 25 bp. Low-quality sequence fragments with lengths shorter than 25 base pairs were filtered out. To ensure accuracy, only uniquely mapped reads were retained by using the --outSAMmultNmax 1 parameter to discard multi-mapped reads (mapped to ≥ 2 genomic loci). Qualimap (v2.2.1) was employed to assess read alignment quality, evaluating coverage uniformity and strand specificity. For differential expression analysis, read counts from uniquely mapped reads were processed using DESeq2 (v1.30.1) with median-of-ratios normalization. The Benjamini-Hochberg (BH) correction was applied for multiple testing. Only the uniquely mapped reads were kept for the subsequent analyses (cut-off: log2 Fold Change < 0.5 & adjusted *P* < 0.05).

### 4.4 Deletion mutant construction

Sucrose counter-selection vector pK18mobsacB was digested by using *EcoR*I and *BamH*I [35], [37]. The upstream 1500 bp and downstream 1500 bp fragments of the opening reading frame (ORF) of the HSP genes including RS16350 to be deleted were amplified by using corresponding primers (Table S3) from *Psa*_M228 genome. The upstream and downstream fragments and pK18mobsacB (*EcoR*I/*BamH*I) are linked by ligase to form recombinant plasmids. The recombinant plasmids were electroporated into the wild type *Psa*_M228 with selection for Km resistance. The single colonies were picked to a LB-agar plate amend with 5 % sucrose, and then cultured in both LB-agar with 100 μg/mL Km and unamended LB-agar. The single colonies that could not survive in presence of Km but could grow on LB-agar plates were further checked by PCR for the presence of mutation and used in subsequent qRT-PCR experiments.

### 4.5 Complemented strains construction

The pHM1 plasmid was used to construct the complemented strain as we previously reported [37]. Briefly, the opening reading frame (ORF) of RS16350 was amplified form *Psa*_M228 genomic DNA by PCR used a pair of primers pHM1-TrpR2(M228)-F/R (Table S3). The fragments were cloned into *Hind*III digested pHM1 to generate pHM1-TrpR2 plasmid. Then, the pHM1-TrpR2 plasmid was transformed into RS16350 deletion mutant to generated RS16350 complemented strain.

### 4.6 Amino acid point mutations

The point mutation of RS16350 was achieved by overlap Extension PCR as previously described [30]. Primers (forward and reverse) were designed in order to contain mutant bases at the mutation site. Subsequently, segmented PCR of the target was DNA performed with primers to generate two overlapping fragments with mutation sites. The two fragments were then mixed and extended by PCR to form intact mutant DNA. Lastly, Each of products was cloned into *Hind*III digested pHM1 to generate a series of point mutated pHM1-TrpR2 plasmids, and the mutation was verified by sequencing.

### 4.7 Quantitative reverse transcription PCR (qRT-PCR)

RNA purification was performed by using Bacteria Total RNA Isolation Kit (Sangon Biotech). The cDNA synthesis was performed using a HiScript II Q RT SuperMix (Vazyme). The reactions were run at 50℃ for 15 min, 85℃ for 5 s, and kept at 4℃ until used. The qRT-PCR was performed by ChamQ SYBR Color qPCR Master Mix Kit (Vazyme) and prepared by following the manufacturer’s instruction. The volume of each reaction was 10 μL, with 300 ng cDNA and 16S rRNA as the internal control and 100 nM primers were used for each reaction. The fold change represents relative expression level of mRNA, which can be estimated by the threshold cycle (Ct) values of 2^-(∆∆Ct)^ [37].

### 4.8 Lux-reporter assay

The *lux* activity of the samples was measured as previously described [48]. Briefly, the promoter regions of T3SS genes, including *hopY1*, *hopAK1*, *hrpZ1* and *hrpK1*, were respectively cloned into the promoter-less pMS402 (*BamH*I) plasmid by ClonExpress II One Step Cloning Kit (Vazyme, China) to generate corresponding luciferase (*lux*)-based reporter plasmids. *Psa*_M228 with plasmid of luciferase (*lux*)-based reporter were cultured in LB (100 μg/mL Km, 25 μg/mL Rifampicin) at 28℃ to an OD_600_ of 0.8–1.5, then washed three times with the minimal medium (MM) and a 100 μL-aliquot from each of the samples was added to two sterile black transparent bottom 96-well plate at OD_600_ of 0.4–0.6 in MM. One plate was cultured at 16℃ and the other at 28℃ for 9 h. The luminance and OD_600_ of all samples were measured in a Synergy 2 plate reader (BioTek) at the same time.

### 4.9 Leaf-disc inoculation assay

Leaf-disc inoculation assay was performed as the previous study with minor modification [49]. The bacteria carrying plasmid pPROBE-GFP or pKD-*hopY1-lux* were cultured in LB (100 μg/mL Km, 100 μg/mL Spe) until reached an bacteria solution OD_600_ of 0.8–1.2. Then washed twice with sterilized distilled H_2_O and adjusted OD_600_ to 0.1. The leaves newly grown within two weeks of *Actinidia chinensis* ‘Hongyang’ were selected, punched into 1.5 cm disc and inoculated with bacteria solution (OD_600_=0.1) of M228 by vacuum infiltration. For each transformant strains, three leaf discs were placed on 0.5 % water agar plate and kept in a climatic cabinet at 16℃ with 70 % relative humidity and 8 hours of sunshine per day for 5–7 days before the incidence of disease was observed. The lesion area and fluorescence area of the leaf discs were obtained by gray scale analysis and calculation of ImageJ software.

### 4.10 Construction of fluorescence tagged strains

The methods of the construction of fluorescence-tagged strains followed our previous study [38]. The plasmid pPROBE carrying the gene encoding green fluorescent protein (GFP) were electroporated into M228. Transformants were cultured on LB agar plates (100 μg/mL Km) at 28℃ for 48 h.

### 4.11 Growth curve assay

The bacteria were cultured in LB until they reached an OD_600_ of 2.0, and then 200 µL were absorbed with a pipette and added to conical bottle containing 20 mL LB, cultured at 16℃ or 28℃. OD_600_ values were measured with spectrophotometer every 4 h at 16℃ or 28℃. Each group of bacteria was tested in duplicate.

### 4.12 Bacterial two-hybrid assay

The BacterioMatch II two-hybrid (B2H) system (Agilent Technologies, USA) was used to detect protein-protein interactions [51]. The coding region of TrpR2 target protein was cloned into the pBT plasmid, and the coding region of HrpR, HrpS or HrpL target proteins was cloned into the pTRG plasmid, which was transformed into *E.coli* strain XL1-Blue. Plasmids pBT-GacS and pTRG-GacS were carried by the positive control strain and the empty pTRG and pBT were carried by the negative control strain. The co-transformants were spotted onto the selective medium: M9 supplemented with 12.5 μg/mL Tc, 34 μg/mL Cm, 30 μg/mL Km, 8 μg/mL Str and 5 mM 3-AT (3-amino-1,2,4-triazole) grown at 28℃ for 1–2 days.

### 4.13 Purification of TrpR2

GST tagged TrpR2 protein expression and purification were performed followed previous protocols [52]. The coding sequence of TrpR2 was inserted into the pGEX-6P-1 plasmid, utilizing the primers detailed in Table S2. These plasmids were then introduced into *E. coli* BL21 (DE3) strains (Table S1). Bacterial cultures were cultivated until they reached an optical density at 600 nm (OD_600_) of 0.6 at 37℃. Subsequently, they were induced with 0.5 mM isopropyl β-D-1-thiogalactopyranoside (IPTG, Sigma, USA) at 16℃ for a duration of 16 h. The GST-fused TrpR2 was purified from 300 mL *E. coli* BL21 (DE3) culture using GST resin (GE Healthcare, Shanghai, China). The purity of the proteins was assessed through Sodium Dodecyl Sulfate-Polyacrylamide Gel Electrophoresis (SDS-PAGE), and the protein concentration was determined using a BCA protein assay kit (Sangon Biotech, Shanghai, China).

### 4.14 Purification of RNAP-HrpL holoenzyme

RNAP-HrpL holoenzyme was purified as previously described [46]. Briefly, the RNAP core enzyme (6His-β’) and His6X tagged HrpL were co-expressed in *E. coli* BL21 (DE3) from pVS10 and pET28a-*hrpL* via induction with 0.5 mM IPTG. Cell pellets were resuspended in buffer A (100 mM Tris-HCl pH 7.4, 500 mM NaCl, 5 % glycerol and 0.1 mM EDTA), disrupted by sonication and the resultant soluble fraction purified by metal affinity chromatography (His-Trap HP column, GE Healthcare) using a linear gradient of 0–1 M imidazole (in buffer A). The desired protein fractions were dialysed twice against storage buffer (50 mM Tris-HCl pH 8, 100 mM NaCl, 20 % glycerol (w/v), 1 mM dithiothreitol and 0.1 mM EDTA).

### 4.15 Microscale thermophoresis assay (MST)

Protein-protein binding affinities were determined using Monolith NT.115 as previous protocols [52]. Briefly, the RNAP-HrpL holoenzyme His-tagged protein was labeled with the fluorescent dye RED-Tris-NTA (MO-L018). A constant concentration of the labeled protein (50 nM) in MST buffer was titrated against GST-TrpR2 protein or GST protein at concentrations ranging from 0.38 nM to 25 μM. MST premium-coated capillaries (MO-K005) were used to load the samples into the MST instrument at 25℃ with medium MST power and 90 % LED power. Laser on and off times were set at 10 s. All experiments were performed in triplicate, and data were analyzed using MO. Affinity software.

### 4.16 Interaction validation assay for HrpL and TrpR2

HrpL and TrpR2 were co-cloned into pETDuet plasmid to generated pETDuet- HrpL+TrpR2, and the HrpL was cloned into the pETDuet alone to generate pETDuet- HrpL (control). The generated plasmids were respectively transferred into *E. coli* BL21 (DE3) together with pKD-*hopY1-lux* to generate HrpL/*hopY1-lux* strains and HrpL+TrpR2/*hopY1-lux* strains. A single colony was picked and shaken to an OD_600_ of 0.4–0.6 in LB, and then 0. 4 μM IPTG was added. A 100 μL-aliquot from each of the samples was added to identical sterile black transparent bottom 96-well plate. Then the plate with shaking at 220 rpm,16℃ for 8 h. The luminance and OD_600_ of all samples were detected by OD_600_ in a Synergy 2 plate reader (BioTek) at the same time.

## CRediT authorship contribution statement

**Limin Wang:** Writing – review & editing. **Aprodisia Murero:** Methodology, Investigation. **Cheng Chen:** Writing – review & editing. **Xiaolong Shao:** Writing – review & editing, Supervision, Project administration, Funding acquisition, Conceptualization. **Yuan Xu:** Writing – review & editing. **Xin Deng:** Writing – review & editing. **Yiqing Ding:** Writing – review & editing, Methodology, Data curation. **Lili Huang:** Writing – review & editing. **Tingtao Chen:** Methodology, Investigation. **Guoliang Qian:** Writing – review & editing, Funding acquisition. **Tiantian Zhang:** Methodology, Investigation. **Li Li:** Writing – review & editing. **Yifei Zhang:** Writing – review & editing, Writing – original draft, Methodology, Investigation, Data curation. **Jinghui Yang:** Writing – review & editing. **Xueting He:** Writing – review & editing, Writing – original draft, Methodology, Investigation, Data curation. **Kaidi Fu:** Writing – review & editing, Writing – original draft, Data curation. **Caihong Zhong:** Writing – review & editing. **Chenbei Xu:** Writing – review & editing, Writing – original draft, Data curation.

## Declaration of Competing Interest

The authors declare that they have no competing interests.

## Data Availability

RNA-seq data are available in the National Center for Biotechnology Information Gene Expression Omnibus database under accession codes GSE283068. The data that supports the findings of this study are available in the supplementary material of this article.
