# Supplementary material for: A temperature-responsive regulator that enhances virulence in the kiwifruit canker pathogen Pseudomonas syringae pv. actinidiae
Source: Comput Struct Biotechnol J. 2025 May 15;27:1935–44. doi: 10.1016/j.csbj.2025.05.017 (PMC12145522; doi:10.1016/j.csbj.2025.05.017)
Supplement: Supplementary file 1 — Supplementary material [file mmc1.docx]

**Supplementary information**

**A Temperature-Responsive Regulator that Enhances Virulence in the Kiwifruit Canker Pathogen *Pseudomonas syringae* pv. *actinidiae***

Xueting He ^1, #^, Yifei Zhang^1, #^, Chenbei Xu^1^, Kaidi Fu^1^, Yiqing Ding^2^, Tiantian Zhang^1^, Tingtao Chen^1^, Aprodisia Murero^1^, Limin Wang^1^, Yuan Xu^3^, Cheng Chen^3^, Jinghui Yang^3^, Li Li^4^, Caihong Zhong^4^, Lili Huang^5^, Xin Deng^6, 7^, Xiaolong Shao^1, *^, Guoliang Qian^1^

This supplementary information contains 6 supplementary figures and 3 supplementary tables.

**Supplementary figures:**


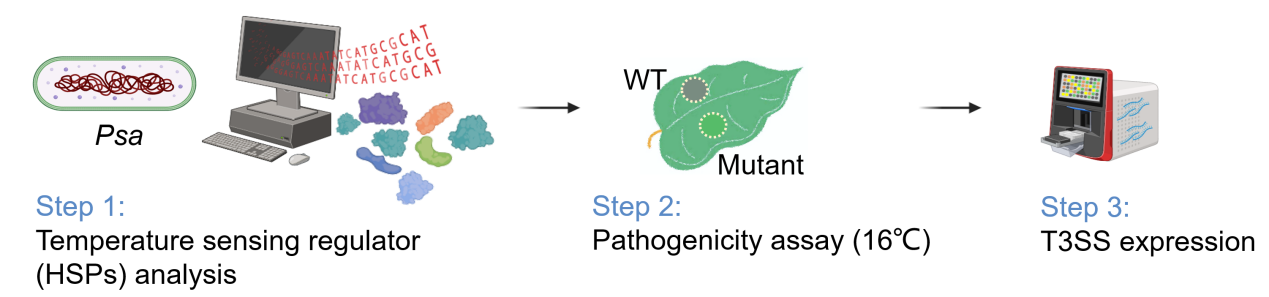


**Fig. S1.** **Schematic workflow of temperature-sensitive regulator screening.** Step 1: temperature-responsive regulators analysis by transcriptome sequencing; Step 1: Screening for temperature-sensing HSPs using a pathogenicity assay on kiwifruit leaves at operates at low temperature (16℃); Step 3: Evaluation of T3SS expression.


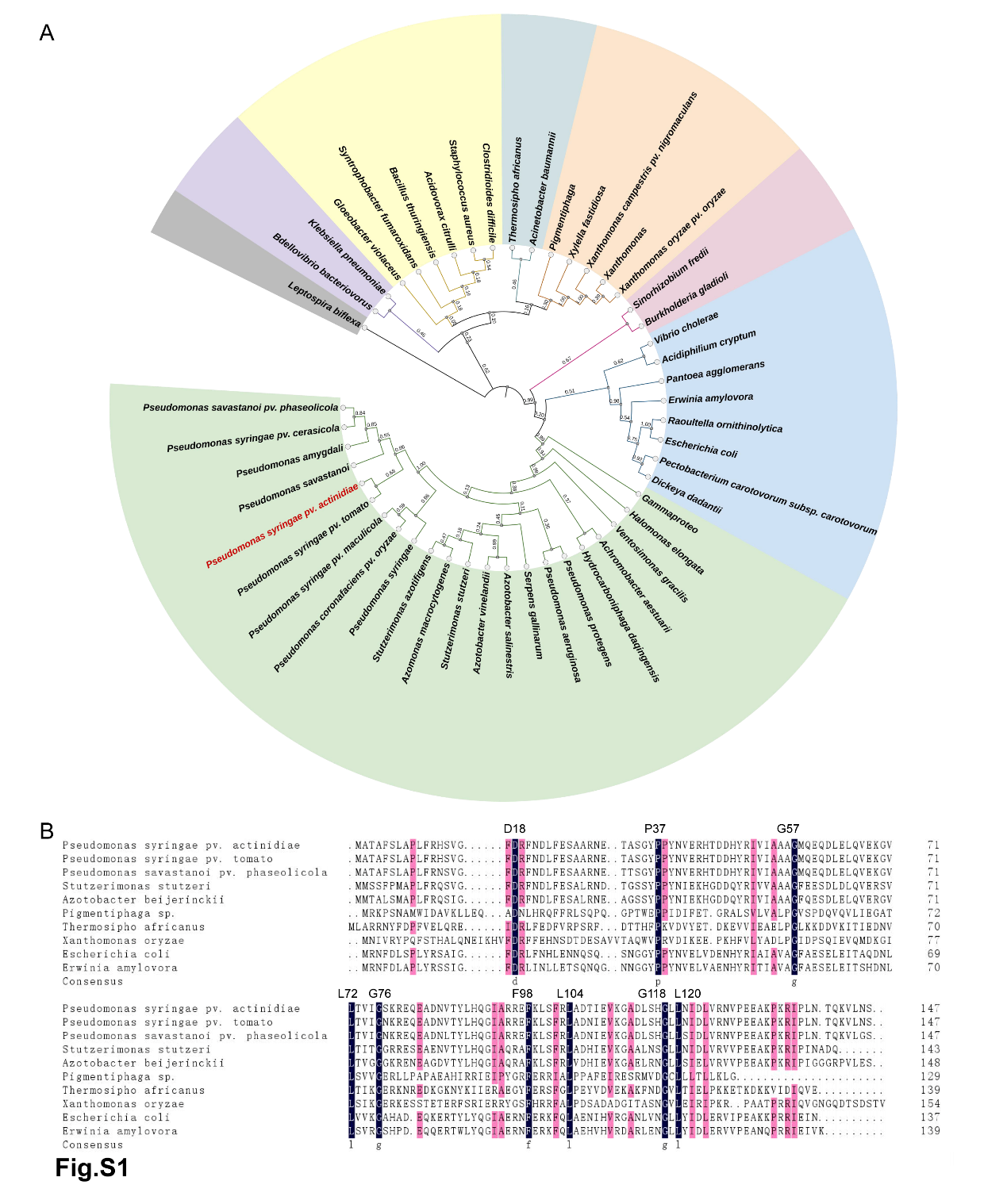


**Fig. S2.** **Phylogenetic analysis and identification of conserved amino acid residues in TrpR2.** (A) The homologous phylogenetic Tree of TrpR2 in Bacterial kingdom. (B) Protein sequence alignment of TrpR2 in 10 species of bacteria (including *Psa*_M228). The protein sequences were found on NCBI and aligned by DNAMAN. The amino acid sites marked black were exactly the same, and the homology rate of the amino acid sites marked pink were more than 75%.


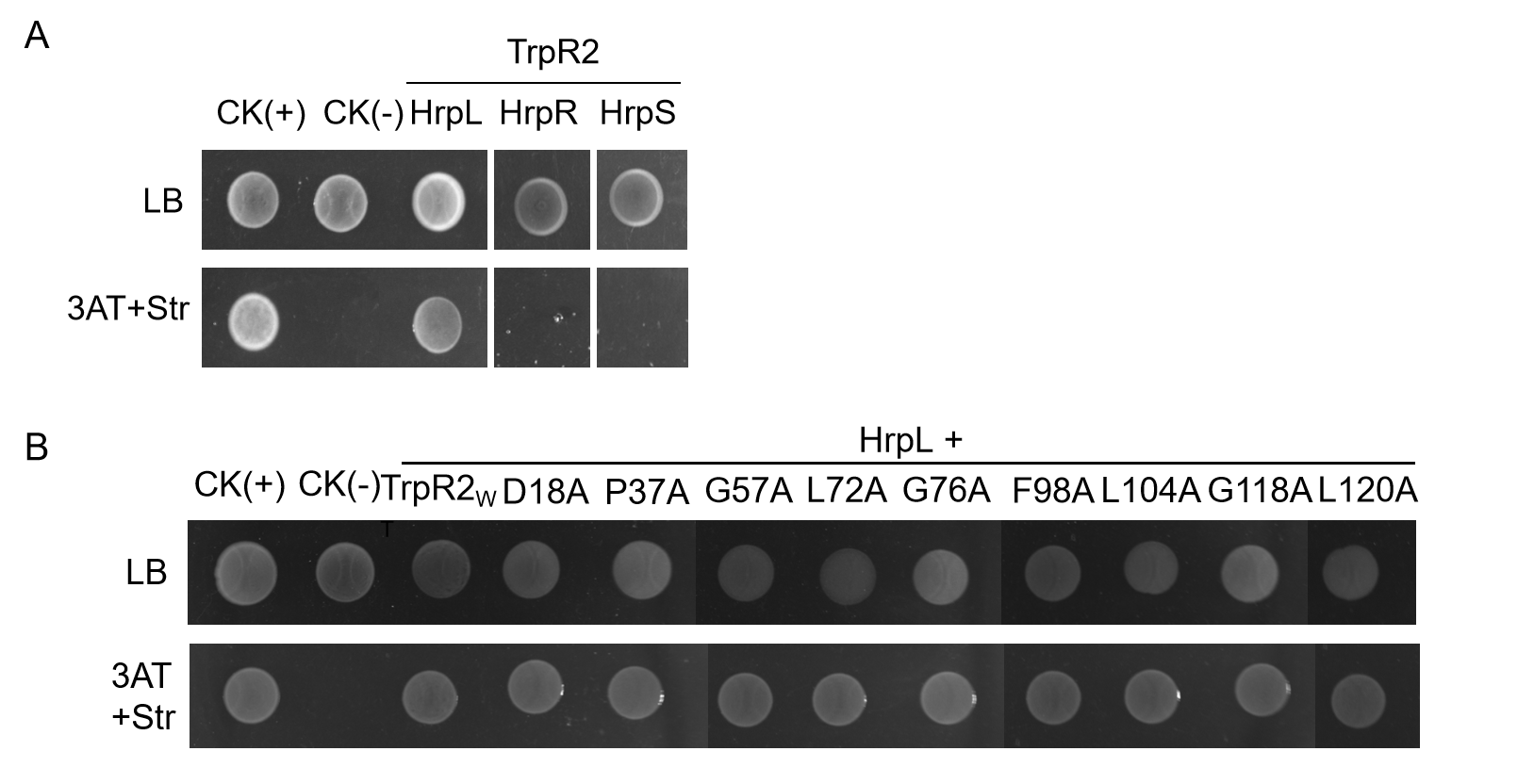
 **Fig. S3.** **Bacterial two-hybrid assay demonstrating that nine conserved candidate residues in TrpR2 do not mediate interaction with HrpL.** (A) Bacterial two-hybrid assays of TrpR2 and HrpR, HrpS, HrpL: only the XL1-Blue co-transformed into pBT-TrpR2 and pTRG-HrpL grew on the selective medium. (B) All experiments were repeated at least three times, with similar results.


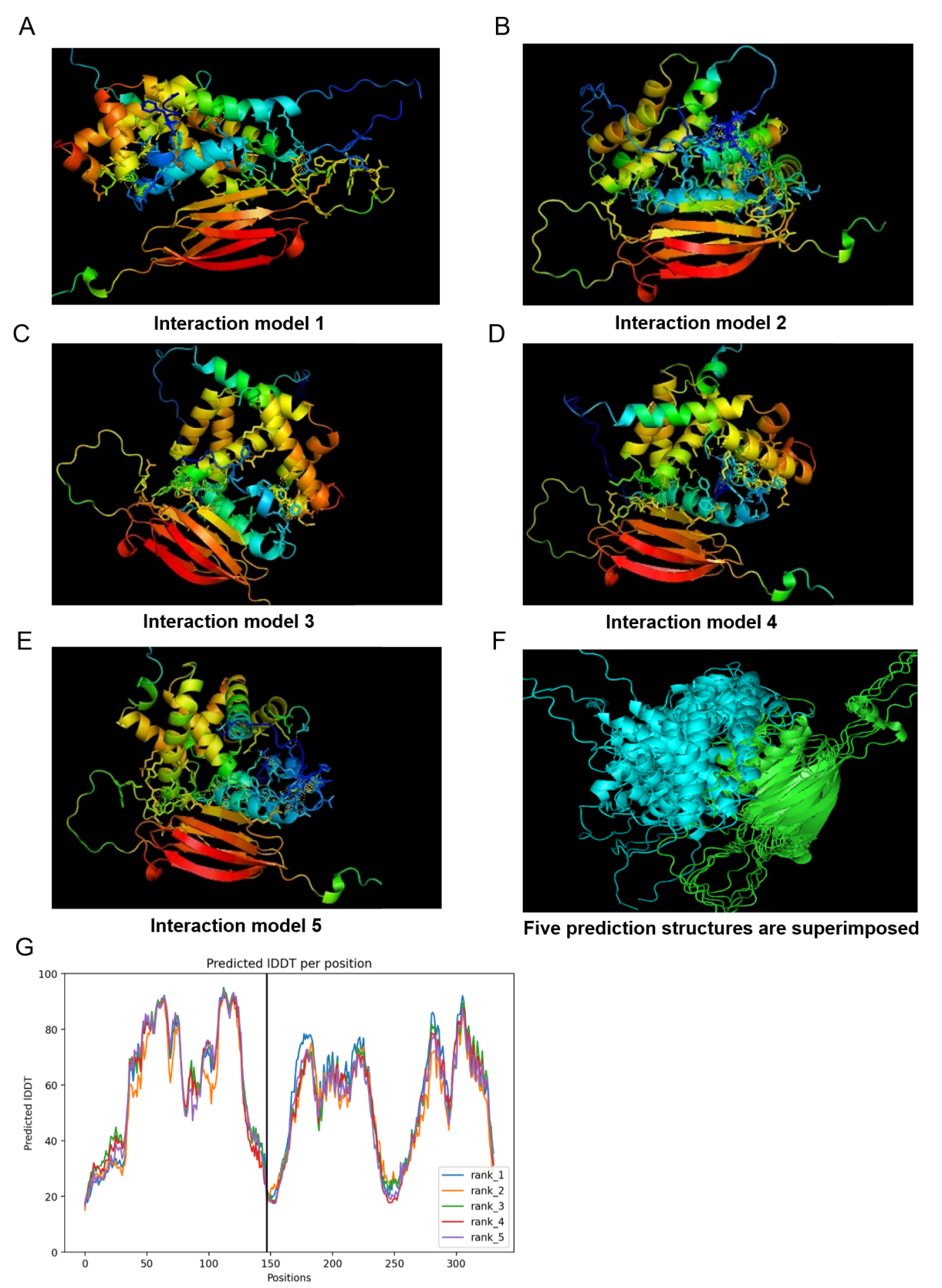
 **Fig. S4. Structural prediction of TrpR2-HrpL interaction complex using molecular docking.** (A-F) Other four Three-dimensional models of interaction between TrpR2*_Psa_* and HrpL*_Psa_*. (G) The predicted Local Distance Difference Test (pLDDT) of different segments of CN228_RS16350-HrpL models is one of the indexes used to evaluate the quality of protein structure prediction. It is mainly used to evaluate the local structural accuracy of the prediction. pLDDT was introduced by DeepMind's AlphaFold2 team and is widely used to assess the accuracy of protein structure predictions.


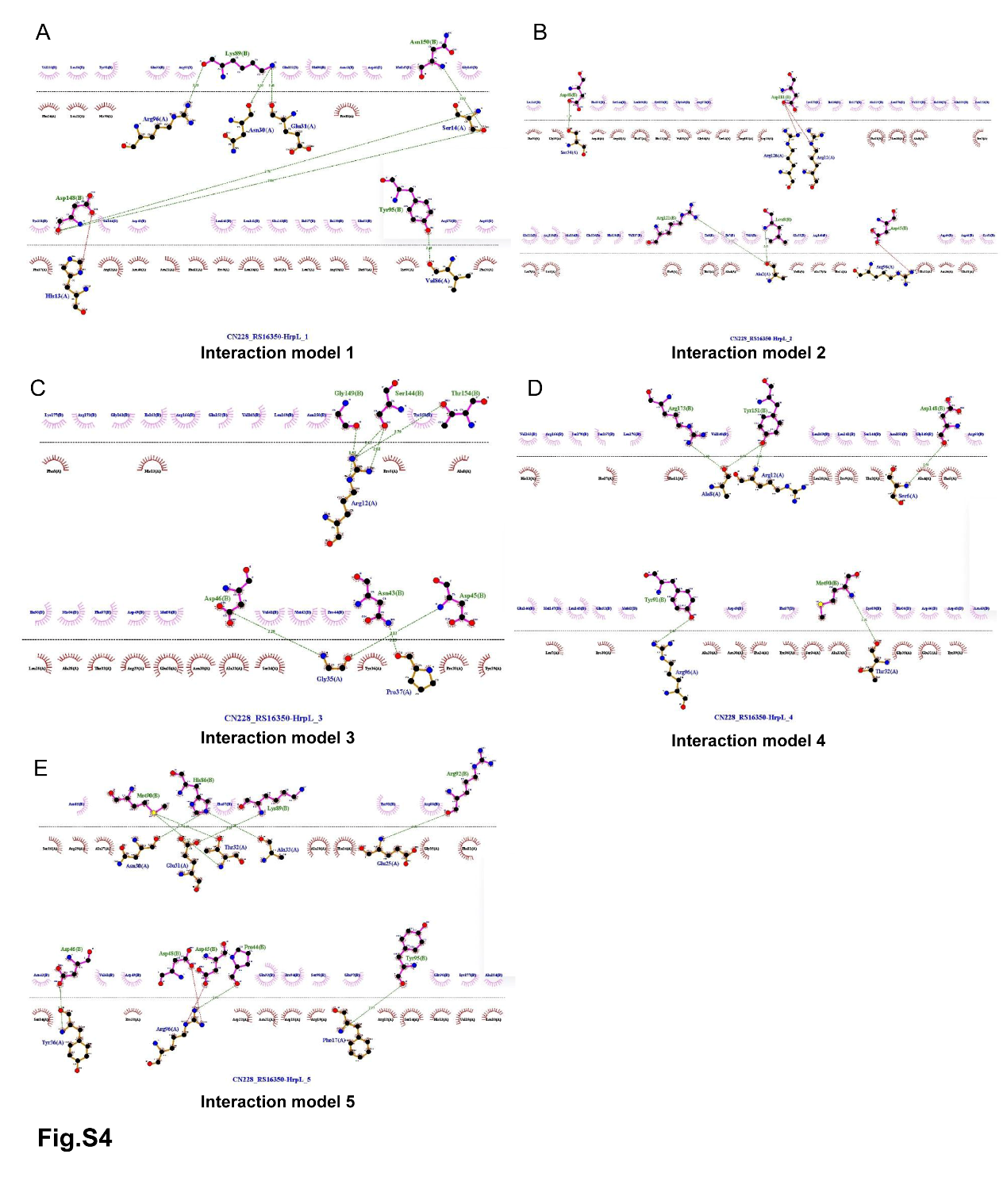


**Fig. S5. Computational prediction of critical interaction interfaces between TrpR2 and HrpL.** (A-E) LigPlus was used to label hydrogen bonds and other hydrophobic forces.


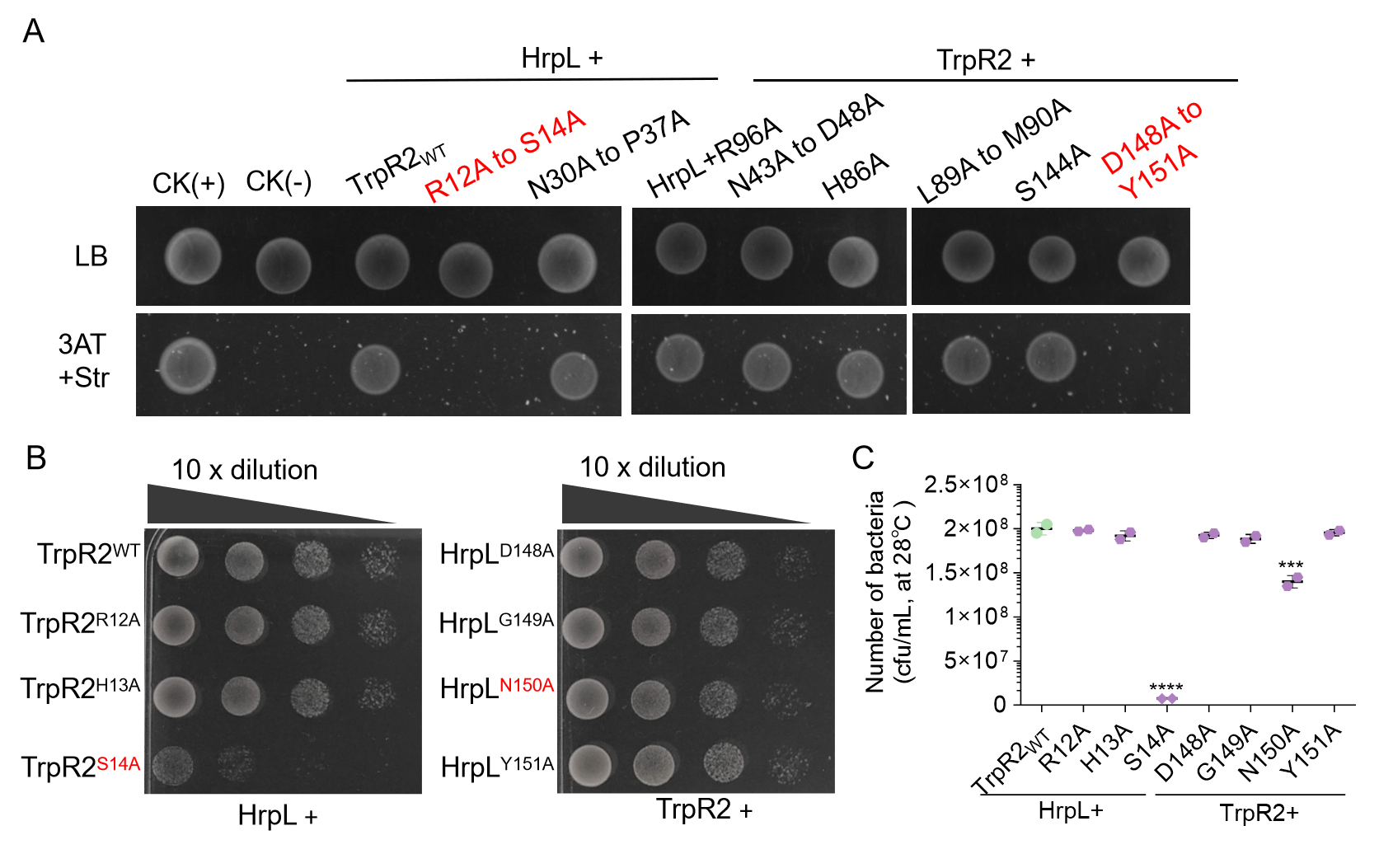


**Fig. S6. Experimental validation of predicted TrpR2-HrpL interaction interfaces through bacterial two-hybrid assay.** (A) Critical interaction interfaces between TrpR2 and HrpL were experimentally validated through bacterial two-hybrid assay. (B) Quantitative assessment of binding affinity between structure-guided TrpR2 mutants and HrpL mutants predicted by molecular docking. (C) Colony-forming units (CFUs) were quantified on selective media containing 5 mM 3-amino-1,2,4-triazole (3-AT) with duplicate technical replicates per experimental group. Data represent mean ± SD (****P < 0.0001, ***P < 0.001 by Student's t-test). Results were consistent across three independent biological replicates.

**Table S1. RNA-seq of *Psa*_M228 (16℃ vs 28℃).**

| **Gene ID** | **log2FoldChange** | **P value** | **adjust**  **P value** | **Gene ID** | **log2FoldChange** | **P value** | **adjust**  **P value** |
| --- | --- | --- | --- | --- | --- | --- | --- |
| RS26595 | -5.12099 | 0.002623 | NA | RS07610 | 0.52043 | 0.002817 | 0.013682 |
| RS21330 | -4.64491 | 0.006168 | NA | RS28540 | 0.522239 | 0.035911 | 0.101976 |
| RS12220 | -4.5333 | 9.32E-06 | 0.000112 | RS21750 | 0.522509 | 0.04601 | 0.124515 |
| RS19525 | -4.49799 | 7.81E-10 | 4.38E-08 | RS18100 | 0.522879 | 0.028799 | 0.08629 |
| RS25330 | -4.10823 | 4.4E-07 | 8.87E-06 | RS18325 | 0.523475 | 0.021494 | 0.068806 |
| RS31980 | -3.82963 | 0.025534 | NA | RS20535 | 0.525134 | 0.011537 | 0.042156 |
| RS15085 | -3.57675 | 0.034542 | NA | RS25305 | 0.526338 | 0.045589 | 0.12368 |
| RS19515 | -3.33508 | 1.47E-11 | 1.53E-09 | RS06875 | 0.527372 | 0.020918 | 0.067681 |
| RS22920 | -3.09378 | 0.008132 | 0.031745 | RS07980 | 0.529379 | 0.011055 | 0.040826 |
| RS11935 | -3.07144 | 6.84E-13 | 1.03E-10 | RS09740 | 0.53558 | 0.019389 | 0.063793 |
| RS10950 | -3.03214 | 1.77E-12 | 2.45E-10 | RS22945 | 0.536405 | 0.001747 | 0.00921 |
| RS25325 | -2.99355 | 2.85E-06 | 4.23E-05 | RS26660 | 0.538587 | 0.012777 | 0.045765 |
| RS12340 | -2.88243 | 4.85E-09 | 1.95E-07 | RS13410 | 0.540173 | 0.049133 | 0.1311 |
| RS11460 | -2.87657 | 0.037604 | NA | RS07375 | 0.540891 | 0.005689 | 0.024158 |
| RS10740 | -2.74158 | 9.06E-12 | 9.67E-10 | RS14670 | 0.541127 | 0.020788 | 0.067335 |
| RS04120 | -2.72786 | 3.31E-06 | 4.8E-05 | RS03675 | 0.542042 | 0.01113 | 0.041033 |
| RS16515 | -2.59319 | 0.000827 | 0.005008 | RS20075 | 0.542909 | 0.048469 | 0.129943 |
| RS06490 | -2.59245 | 3.77E-11 | 3.51E-09 | RS02150 | 0.544066 | 0.011115 | 0.041011 |
| RS10745 | -2.55296 | 1.99E-13 | 4.14E-11 | RS21745 | 0.545499 | 0.016169 | 0.055236 |
| RS23780 | -2.51043 | 3.2E-09 | 1.34E-07 | RS26915 | 0.547061 | 5E-05 | 0.000466 |
| RS25340 | -2.48485 | 4.06E-11 | 3.63E-09 | RS12440 | 0.550391 | 0.007769 | 0.030552 |
| RS12335 | -2.44947 | 2.33E-10 | 1.52E-08 | RS04155 | 0.550686 | 0.002293 | 0.011493 |
| RS13390 | -2.44846 | 6.45E-12 | 7.31E-10 | RS30190 | 0.551823 | 0.005744 | 0.024308 |
| RS11930 | -2.44183 | 0.000118 | 0.000948 | RS13510 | 0.551905 | 0.013011 | 0.046403 |
| RS27085 | -2.41486 | 0.01588 | 0.054416 | RS20455 | 0.553217 | 0.039952 | 0.111215 |
| RS18405 | -2.40357 | 1.36E-21 | 1.99E-18 | RS31535 | 0.554044 | 0.001079 | 0.006229 |
| RS30720 | -2.34405 | 0.005318 | 0.022904 | RS07915 | 0.55504 | 0.007019 | 0.028386 |
| RS31810 | -2.31579 | 1.44E-09 | 7.25E-08 | RS10270 | 0.555384 | 0.001898 | 0.009818 |
| RS19480 | -2.27878 | 1.88E-12 | 2.49E-10 | RS03990 | 0.555631 | 0.017503 | 0.058963 |
| RS13745 | -2.27384 | 4.99E-08 | 1.31E-06 | RS19365 | 0.555797 | 0.035841 | 0.101845 |
| RS31300 | -2.21473 | 2.72E-08 | 8.35E-07 | RS02660 | 0.556329 | 0.005413 | 0.023252 |
| RS04115 | -2.18929 | 7.31E-07 | 1.35E-05 | RS29665 | 0.556557 | 0.035372 | 0.100906 |
| RS23910 | -2.188 | 0.023447 | 0.074028 | RS20205 | 0.558196 | 0.043883 | 0.12002 |
| RS24920 | -2.17802 | 1.99E-18 | 1.25E-15 | RS30865 | 0.558548 | 0.013925 | 0.049063 |
| RS19495 | -2.16074 | 1.01E-07 | 2.51E-06 | RS31715 | 0.55974 | 0.024628 | 0.076927 |
| RS19470 | -2.15666 | 2.57E-08 | 8.22E-07 | RS29365 | 0.561159 | 0.000637 | 0.003961 |
| RS12230 | -2.13591 | 3.36E-09 | 1.4E-07 | RS00275 | 0.561783 | 0.001852 | 0.009648 |
| RS02785 | -2.09602 | 1.06E-10 | 8.41E-09 | RS27140 | 0.562062 | 0.044344 | 0.121053 |
| RS27975 | -2.09417 | 3.16E-09 | 1.34E-07 | RS05620 | 0.564638 | 0.005772 | 0.024405 |
| RS19505 | -2.06068 | 6.37E-06 | 8.1E-05 | RS12070 | 0.565238 | 0.002648 | 0.012993 |
| RS30295 | -2.02744 | 9.59E-06 | 0.000115 | RS28190 | 0.565336 | 0.019987 | 0.065406 |
| RS15130 | -2.01231 | 0.011234 | 0.041311 | RS15465 | 0.565737 | 0.003675 | 0.016982 |
| RS27980 | -2.0003 | 1.28E-08 | 4.55E-07 | RS07580 | 0.566946 | 0.011645 | 0.042429 |
| RS06045 | -1.99239 | 1.17E-07 | 2.81E-06 | RS30315 | 0.567277 | 0.000911 | 0.005401 |
| RS11455 | -1.98882 | 1.22E-08 | 4.43E-07 | RS14020 | 0.568074 | 0.002515 | 0.012391 |
| RS12290 | -1.9858 | 1.18E-05 | 0.000136 | RS13435 | 0.572065 | 0.005959 | 0.025074 |
| RS02780 | -1.98371 | 6.25E-08 | 1.62E-06 | RS07060 | 0.572721 | 0.045776 | 0.123957 |
| RS12330 | -1.97641 | 4.12E-08 | 1.15E-06 | RS03680 | 0.577815 | 0.01964 | 0.064473 |
| RS14500 | -1.94677 | 2.31E-06 | 3.57E-05 | RS23865 | 0.579085 | 0.00223 | 0.011242 |
| RS07485 | -1.92771 | 5.53E-06 | 7.29E-05 | RS15285 | 0.582284 | 0.024396 | 0.076309 |
| RS29160 | -1.92204 | 2.31E-12 | 2.81E-10 | RS06775 | 0.583213 | 0.009976 | 0.037471 |
| RS30290 | -1.90814 | 5.91E-06 | 7.63E-05 | RS03200 | 0.58401 | 0.008531 | 0.032919 |
| RS25575 | -1.90199 | 6.08E-12 | 7.19E-10 | RS31020 | 0.586112 | 0.023661 | 0.07465 |
| RS24925 | -1.89905 | 7.43E-16 | 3.44E-13 | RS12030 | 0.589733 | 0.007771 | 0.030552 |
| RS27505 | -1.88871 | 0.022672 | 0.071944 | RS22540 | 0.589834 | 0.043325 | 0.118941 |
| RS07765 | -1.8779 | 1.88E-10 | 1.31E-08 | RS23015 | 0.591203 | 0.008216 | 0.031989 |
| RS02140 | -1.86354 | 0.019111 | 0.06307 | RS21740 | 0.592773 | 0.001426 | 0.007792 |
| RS27990 | -1.85793 | 1.13E-07 | 2.74E-06 | RS08705 | 0.594574 | 0.03398 | 0.097827 |
| RS23915 | -1.84555 | 2.41E-07 | 5.29E-06 | RS00410 | 0.595316 | 0.02182 | 0.069647 |
| RS22090 | -1.83075 | 5.48E-05 | 0.000505 | RS29555 | 0.596063 | 0.012369 | 0.044512 |
| RS27915 | -1.8236 | 0.000196 | 0.001464 | RS30795 | 0.59841 | 0.006666 | 0.027312 |
| RS25890 | -1.81415 | 8.61E-08 | 2.19E-06 | RS30845 | 0.598585 | 0.010717 | 0.039878 |
| RS25565 | -1.81302 | 3.31E-18 | 1.81E-15 | RS31210 | 0.598777 | 0.004379 | 0.019535 |
| RS12225 | -1.81064 | 0.002102 | 0.010719 | RS26570 | 0.601376 | 0.002147 | 0.0109 |
| RS07775 | -1.78938 | 1.93E-05 | 0.000207 | RS22770 | 0.602802 | 0.030853 | 0.090857 |
| RS27985 | -1.76211 | 2.33E-08 | 7.54E-07 | RS00930 | 0.604184 | 0.003295 | 0.015469 |
| RS02850 | -1.75439 | 2.02E-10 | 1.38E-08 | RS10970 | 0.60419 | 0.008889 | 0.034061 |
| RS25605 | -1.75139 | 0.013551 | 0.047938 | RS22045 | 0.604262 | 0.032098 | 0.093391 |
| RS02845 | -1.74646 | 2.66E-21 | 2.91E-18 | RS23060 | 0.606591 | 0.000532 | 0.003416 |
| RS08340 | -1.74 | 0.000159 | 0.001221 | RS28575 | 0.609102 | 0.00821 | 0.031989 |
| RS22630 | -1.73023 | 0.031588 | 0.092648 | RS18845 | 0.609607 | 0.019999 | 0.065406 |
| RS09195 | -1.72639 | 0.019103 | 0.06307 | RS21635 | 0.611313 | 0.00069 | 0.004271 |
| RS16505 | -1.72634 | 0.005651 | 0.024078 | RS23020 | 0.61209 | 0.012307 | 0.044397 |
| RS18600 | -1.72468 | 0.014589 | 0.050787 | RS21585 | 0.61336 | 0.009078 | 0.034723 |
| RS20795 | -1.7219 | 3.64E-14 | 9.36E-12 | RS24290 | 0.613672 | 0.018066 | 0.060304 |
| RS04360 | -1.7187 | 0.011546 | 0.042156 | RS29195 | 0.614163 | 0.010009 | 0.037503 |
| RS12300 | -1.71206 | 2.52E-06 | 3.85E-05 | RS00535 | 0.614946 | 0.044441 | 0.121167 |
| RS08095 | -1.7 | 1.94E-06 | 3.08E-05 | RS19825 | 0.615371 | 0.011726 | 0.042617 |
| RS13710 | -1.69396 | 1.25E-08 | 4.49E-07 | RS13290 | 0.618514 | 0.014289 | 0.050062 |
| RS25570 | -1.69345 | 6.28E-11 | 5.38E-09 | RS29285 | 0.619349 | 0.000245 | 0.00176 |
| RS16710 | -1.68532 | 7.33E-07 | 1.35E-05 | RS01865 | 0.621014 | 0.000198 | 0.001471 |
| RS02930 | -1.6614 | 4.2E-08 | 1.16E-06 | RS25140 | 0.621749 | 0.001211 | 0.006814 |
| RS25580 | -1.66053 | 2.7E-08 | 8.35E-07 | RS22840 | 0.624964 | 0.001445 | 0.007857 |
| RS16350 | -1.65145 | 1.52E-05 | 0.000169 | RS26280 | 0.625414 | 0.000331 | 0.002292 |
| RS13700 | -1.64056 | 1.13E-08 | 4.11E-07 | RS24545 | 0.625719 | 0.03065 | 0.090501 |
| RS12235 | -1.63442 | 0.000474 | 0.003087 | RS10350 | 0.627118 | 0.00035 | 0.002386 |
| RS02770 | -1.62652 | 3.01E-07 | 6.39E-06 | RS30770 | 0.627159 | 0.015713 | 0.053936 |
| RS08485 | -1.62331 | 1.31E-05 | 0.000148 | RS04340 | 0.628059 | 0.011886 | 0.043093 |
| RS27790 | -1.62079 | 0.001984 | 0.010178 | RS16330 | 0.628576 | 0.005135 | 0.02236 |
| RS06010 | -1.6161 | 3.36E-05 | 0.000331 | RS23550 | 0.63079 | 0.041503 | 0.114874 |
| RS09560 | -1.60393 | 9.61E-06 | 0.000115 | RS31150 | 0.631192 | 0.003633 | 0.016821 |
| RS02875 | -1.60335 | 2.52E-09 | 1.14E-07 | RS10430 | 0.633241 | 0.000341 | 0.002336 |
| RS10415 | -1.59638 | 0.00055 | 0.003507 | RS28120 | 0.633348 | 0.000512 | 0.003304 |
| RS14495 | -1.59436 | 1.06E-05 | 0.000125 | RS10865 | 0.633519 | 0.011536 | 0.042156 |
| RS25155 | -1.59225 | 2.15E-05 | 0.000226 | RS26835 | 0.636539 | 0.0108 | 0.040076 |
| RS06035 | -1.58554 | 9.22E-06 | 0.000111 | RS07120 | 0.636776 | 0.048521 | 0.129943 |
| RS23905 | -1.57214 | 3.35E-06 | 4.84E-05 | RS21575 | 0.638141 | 0.000103 | 0.000844 |
| RS13460 | -1.56831 | 0.00034 | 0.002335 | RS22835 | 0.638408 | 0.000789 | 0.004807 |
| RS02870 | -1.56731 | 2.97E-07 | 6.34E-06 | RS21865 | 0.639476 | 0.009621 | 0.036356 |
| RS15965 | -1.56467 | 0.003176 | 0.015141 | RS20935 | 0.641416 | 0.028563 | 0.085964 |
| RS05280 | -1.56134 | 4.07E-07 | 8.28E-06 | RS26115 | 0.643122 | 0.006857 | 0.02786 |
| RS20800 | -1.55453 | 0.0183 | 0.060806 | RS29500 | 0.644902 | 0.001208 | 0.00681 |
| RS07225 | -1.55097 | 0.000742 | 0.004551 | RS19875 | 0.645262 | 0.005141 | 0.022363 |
| RS30950 | -1.55003 | 8.75E-07 | 1.54E-05 | RS18555 | 0.645389 | 0.015631 | 0.053774 |
| RS19500 | -1.54733 | 0.000879 | 0.005249 | RS23430 | 0.645401 | 0.002347 | 0.01171 |
| RS25530 | -1.54214 | 2.04E-09 | 9.71E-08 | RS01105 | 0.647279 | 6.3E-05 | 0.000567 |
| RS03650 | -1.52555 | 0.001341 | 0.007398 | RS07710 | 0.648009 | 0.009893 | 0.037223 |
| RS23470 | -1.51989 | 0.026285 | 0.080774 | RS18975 | 0.6481 | 0.016419 | 0.055913 |
| RS02830 | -1.5127 | 2E-06 | 3.17E-05 | RS03355 | 0.648181 | 0.00554 | 0.023631 |
| RS06055 | -1.50855 | 0.026866 | 0.082161 | RS21675 | 0.648937 | 0.003197 | 0.015173 |
| RS02915 | -1.50412 | 4.31E-07 | 8.72E-06 | RS21355 | 0.648976 | 0.001194 | 0.006749 |
| RS07480 | -1.49553 | 7.51E-05 | 0.000652 | RS25970 | 0.652096 | 0.039918 | 0.11119 |
| RS16665 | -1.49193 | 2.52E-07 | 5.51E-06 | RS03860 | 0.652831 | 0.033039 | 0.095811 |
| RS10735 | -1.48879 | 0.000266 | 0.001894 | RS12120 | 0.654616 | 0.005444 | 0.023309 |
| RS02840 | -1.48665 | 4.69E-08 | 1.26E-06 | RS11275 | 0.654698 | 0.017764 | 0.059612 |
| RS30065 | -1.47721 | 1.28E-09 | 6.67E-08 | RS28795 | 0.656682 | 0.012017 | 0.043506 |
| RS02825 | -1.46903 | 5.3E-06 | 7.03E-05 | RS21735 | 0.657718 | 0.027885 | 0.084681 |
| RS02855 | -1.46576 | 4.89E-08 | 1.3E-06 | RS24480 | 0.658644 | 0.030772 | 0.090741 |
| RS12675 | -1.4657 | 6.41E-06 | 8.13E-05 | RS29000 | 0.658903 | 0.022099 | 0.070383 |
| RS23845 | -1.44164 | 0.039065 | 0.109023 | RS29535 | 0.659436 | 0.002659 | 0.013014 |
| RS19410 | -1.4406 | 0.017586 | 0.059198 | RS20295 | 0.66124 | 0.001719 | 0.009094 |
| RS13370 | -1.43181 | 9.96E-07 | 1.72E-05 | RS16905 | 0.662384 | 0.012166 | 0.043961 |
| RS25895 | -1.42912 | 1.6E-05 | 0.000177 | RS04560 | 0.664133 | 0.034767 | 0.099698 |
| RS10585 | -1.42833 | 0.00218 | 0.011042 | RS10655 | 0.664371 | 0.000233 | 0.001686 |
| RS29155 | -1.4274 | 5.21E-08 | 1.36E-06 | RS01005 | 0.665738 | 0.01531 | 0.052879 |
| RS16510 | -1.42518 | 0.028067 | 0.085114 | RS12510 | 0.665759 | 0.001395 | 0.00767 |
| RS11020 | -1.42081 | 2.06E-07 | 4.61E-06 | RS05100 | 0.670359 | 0.023297 | 0.073662 |
| RS23155 | -1.42081 | 0.00019 | 0.001428 | RS15685 | 0.670563 | 8.05E-05 | 0.000693 |
| RS26775 | -1.41787 | 0.020969 | 0.067719 | RS12100 | 0.671141 | 0.003274 | 0.015455 |
| RS05270 | -1.41617 | 1.3E-05 | 0.000148 | RS05480 | 0.673747 | 0.004241 | 0.019074 |
| RS07490 | -1.41593 | 5.31E-07 | 1.04E-05 | RS00960 | 0.676518 | 0.04624 | 0.124857 |
| RS02005 | -1.41031 | 2.93E-08 | 8.83E-07 | RS24085 | 0.677061 | 0.008726 | 0.033642 |
| RS07410 | -1.39452 | 0.000283 | 0.001994 | RS26045 | 0.677556 | 0.01445 | 0.050464 |
| RS14320 | -1.39344 | 0.000118 | 0.000948 | RS05540 | 0.677572 | 7.37E-05 | 0.000642 |
| RS04945 | -1.39193 | 3.06E-08 | 9.12E-07 | RS20320 | 0.677772 | 0.000575 | 0.003629 |
| RS22445 | -1.38549 | 6.64E-11 | 5.59E-09 | RS21915 | 0.677809 | 0.028663 | 0.086028 |
| RS02280 | -1.38425 | 5.71E-06 | 7.44E-05 | RS12475 | 0.678144 | 0.009595 | 0.036309 |
| RS02860 | -1.38194 | 7.37E-07 | 1.36E-05 | RS17555 | 0.67847 | 0.00712 | 0.028601 |
| RS08490 | -1.3776 | 2.3E-10 | 1.52E-08 | RS18565 | 0.679665 | 0.000149 | 0.001159 |
| RS17685 | -1.37016 | 4.53E-06 | 6.17E-05 | RS03250 | 0.680628 | 0.0096 | 0.036309 |
| RS23995 | -1.36614 | 0.007959 | 0.031157 | RS03455 | 0.681323 | 0.016903 | 0.05725 |
| RS14350 | -1.36489 | 9.46E-05 | 0.00079 | RS13090 | 0.682864 | 0.006179 | 0.025776 |
| RS30645 | -1.36015 | 0.000792 | 0.004822 | RS11680 | 0.683601 | 0.042132 | 0.116249 |
| RS16500 | -1.35803 | 0.006544 | 0.02689 | RS22900 | 0.6856 | 0.022781 | 0.072188 |
| RS29130 | -1.35736 | 0.004208 | 0.018963 | RS28675 | 0.685618 | 0.019214 | 0.063315 |
| RS16165 | -1.35443 | 5.58E-07 | 1.08E-05 | RS02060 | 0.689121 | 0.004805 | 0.021133 |
| RS25525 | -1.35388 | 8.28E-07 | 1.47E-05 | RS05795 | 0.689634 | 0.010061 | 0.037663 |
| RS19300 | -1.35151 | 0.020695 | 0.067133 | RS10460 | 0.691632 | 0.005348 | 0.023011 |
| RS02900 | -1.35036 | 1.55E-07 | 3.56E-06 | RS13285 | 0.691888 | 0.001898 | 0.009818 |
| RS16520 | -1.34397 | 0.003526 | 0.016397 | RS23545 | 0.692219 | 0.00019 | 0.001428 |
| RS21035 | -1.34324 | 0.000278 | 0.001967 | RS17645 | 0.692808 | 0.001372 | 0.007552 |
| RS00620 | -1.33306 | 1.45E-07 | 3.35E-06 | RS21835 | 0.692933 | 0.005187 | 0.02245 |
| RS18475 | -1.33198 | 0.001826 | 0.009566 | RS06990 | 0.693002 | 0.003562 | 0.016546 |
| RS18560 | -1.33149 | 7.87E-16 | 3.44E-13 | RS21910 | 0.695203 | 0.000408 | 0.00272 |
| RS02895 | -1.33119 | 7.47E-06 | 9.28E-05 | RS23405 | 0.696763 | 0.000928 | 0.005486 |
| RS22640 | -1.32804 | 0.002191 | 0.011084 | RS06915 | 0.698552 | 0.019388 | 0.063793 |
| RS00455 | -1.32356 | 1.74E-10 | 1.23E-08 | RS10025 | 0.700399 | 0.030354 | 0.089988 |
| RS23165 | -1.31555 | 0.008346 | 0.032376 | RS07290 | 0.701025 | 0.020622 | 0.067057 |
| RS02910 | -1.31008 | 2.3E-07 | 5.07E-06 | RS13025 | 0.701192 | 0.026999 | 0.082505 |
| RS24240 | -1.3067 | 5.38E-07 | 1.05E-05 | RS27015 | 0.70151 | 0.033752 | 0.097491 |
| RS04915 | -1.29803 | 1.28E-05 | 0.000146 | RS16360 | 0.702766 | 0.005697 | 0.024158 |
| RS19700 | -1.29578 | 1.12E-05 | 0.000131 | RS19815 | 0.703435 | 0.013118 | 0.046748 |
| RS02365 | -1.28414 | 3.93E-07 | 8.04E-06 | RS25665 | 0.704097 | 0.042346 | 0.11669 |
| RS16085 | -1.27743 | 0.003051 | 0.014633 | RS24685 | 0.705978 | 0.004003 | 0.01826 |
| RS07145 | -1.2766 | 1.88E-08 | 6.33E-07 | RS09825 | 0.706377 | 0.012739 | 0.045693 |
| RS07475 | -1.27212 | 0.000707 | 0.00436 | RS22710 | 0.707403 | 0.000366 | 0.002476 |
| RS10580 | -1.26985 | 1.65E-05 | 0.000181 | RS31990 | 0.711029 | 0.003769 | 0.017335 |
| RS16660 | -1.26579 | 5.26E-05 | 0.000488 | RS16970 | 0.711867 | 0.036217 | 0.102647 |
| RS16150 | -1.26148 | 2.64E-06 | 3.97E-05 | RS07670 | 0.714529 | 0.000332 | 0.002292 |
| RS28915 | -1.26148 | 3.28E-05 | 0.000325 | RS29830 | 0.716559 | 0.008367 | 0.03243 |
| RS02885 | -1.25851 | 2.03E-07 | 4.59E-06 | RS17540 | 0.717434 | 0.007754 | 0.030552 |
| RS29920 | -1.25057 | 8.07E-06 | 9.95E-05 | RS23170 | 0.718555 | 0.002852 | 0.01382 |
| RS15405 | -1.24999 | 0.004516 | 0.020042 | RS14875 | 0.721246 | 0.005459 | 0.023352 |
| RS29135 | -1.24916 | 1.66E-05 | 0.000181 | RS07045 | 0.722188 | 0.01786 | 0.059797 |
| RS04910 | -1.24506 | 0.000455 | 0.002988 | RS29310 | 0.724487 | 9.1E-05 | 0.000767 |
| RS11035 | -1.23791 | 4.17E-09 | 1.71E-07 | RS25915 | 0.724848 | 0.025648 | 0.0796 |
| RS10285 | -1.23772 | 0.013546 | 0.047938 | RS26805 | 0.725923 | 2.37E-05 | 0.000246 |
| RS02820 | -1.23719 | 0.000148 | 0.001149 | RS12530 | 0.726254 | 0.007458 | 0.02975 |
| RS22955 | -1.23715 | 3.84E-05 | 0.000371 | RS02195 | 0.727196 | 0.000518 | 0.003334 |
| RS08555 | -1.23656 | 0.002406 | 0.011951 | RS03495 | 0.729334 | 0.002713 | 0.013254 |
| RS22780 | -1.23502 | 4.5E-05 | 0.000428 | RS22220 | 0.729978 | 0.006435 | 0.026615 |
| RS08595 | -1.23475 | 0.028692 | 0.086056 | RS05765 | 0.732418 | 0.01057 | 0.039401 |
| RS05045 | -1.23286 | 2.03E-05 | 0.000215 | RS20445 | 0.732695 | 0.000352 | 0.002397 |
| RS02880 | -1.23271 | 1.17E-06 | 2.01E-05 | RS09735 | 0.732738 | 0.000412 | 0.002743 |
| RS02920 | -1.23129 | 2.51E-06 | 3.85E-05 | RS00065 | 0.732812 | 0.006815 | 0.027739 |
| RS24245 | -1.22617 | 1.74E-07 | 3.96E-06 | RS10245 | 0.733754 | 0.001135 | 0.006494 |
| RS30180 | -1.22246 | 3.51E-07 | 7.39E-06 | RS20950 | 0.733795 | 0.000783 | 0.004779 |
| RS29145 | -1.22139 | 4.75E-07 | 9.41E-06 | RS19975 | 0.734505 | 0.035748 | 0.101646 |
| RS26510 | -1.22051 | 3.7E-06 | 5.28E-05 | RS24680 | 0.735101 | 0.000619 | 0.003861 |
| RS16035 | -1.22038 | 0.016982 | 0.057384 | RS31285 | 0.736244 | 0.006618 | 0.027142 |
| RS10555 | -1.21506 | 6.5E-07 | 1.21E-05 | RS19905 | 0.736599 | 0.001343 | 0.007404 |
| RS03015 | -1.20449 | 1.44E-08 | 4.95E-07 | RS18580 | 0.739966 | 5.11E-05 | 0.000475 |
| RS24250 | -1.19982 | 4.39E-05 | 0.00042 | RS14360 | 0.741516 | 0.001453 | 0.00788 |
| RS02800 | -1.19869 | 4.97E-05 | 0.000465 | RS01705 | 0.741913 | 0.000121 | 0.000965 |
| RS02905 | -1.19547 | 0.000298 | 0.002087 | RS30150 | 0.742471 | 0.025116 | 0.078169 |
| RS28275 | -1.19377 | 3.82E-08 | 1.09E-06 | RS05105 | 0.745696 | 9.87E-05 | 0.000816 |
| RS23745 | -1.19248 | 0.016301 | 0.055599 | RS08455 | 0.745815 | 0.003724 | 0.017192 |
| RS07850 | -1.18993 | 1.89E-09 | 9.09E-08 | RS12910 | 0.747629 | 0.00705 | 0.028461 |
| RS24960 | -1.18851 | 0.01278 | 0.045765 | RS11730 | 0.750213 | 0.000228 | 0.001664 |
| RS19000 | -1.18753 | 0.00144 | 0.007846 | RS26965 | 0.750282 | 0.019132 | 0.06309 |
| RS11005 | -1.18587 | 2.23E-06 | 3.47E-05 | RS25375 | 0.752657 | 0.000277 | 0.001966 |
| RS07385 | -1.1853 | 7.03E-05 | 0.000623 | RS19260 | 0.75273 | 7.23E-05 | 0.000637 |
| RS12320 | -1.17069 | 0.004124 | 0.01872 | RS22600 | 0.752865 | 0.000534 | 0.003419 |
| RS05030 | -1.16651 | 5.36E-10 | 3.17E-08 | RS25590 | 0.753182 | 1.52E-05 | 0.000169 |
| RS08125 | -1.16533 | 0.000472 | 0.00308 | RS02625 | 0.753551 | 0.024308 | 0.076088 |
| RS07275 | -1.16482 | 1.41E-08 | 4.9E-07 | RS09535 | 0.754584 | 0.001157 | 0.00659 |
| RS07845 | -1.16366 | 7.47E-07 | 1.37E-05 | RS26090 | 0.757311 | 0.003288 | 0.015459 |
| RS11030 | -1.16062 | 5.16E-10 | 3.09E-08 | RS15900 | 0.758993 | 0.009402 | 0.035807 |
| RS14385 | -1.1586 | 5.67E-06 | 7.41E-05 | RS23860 | 0.760582 | 7.14E-05 | 0.000631 |
| RS07840 | -1.15769 | 1.95E-05 | 0.000209 | RS29170 | 0.761015 | 0.002395 | 0.011919 |
| RS18115 | -1.15663 | 0.015661 | 0.053834 | RS05925 | 0.761148 | 0.011822 | 0.042897 |
| RS25485 | -1.15656 | 0.002428 | 0.012045 | RS26830 | 0.761282 | 0.00288 | 0.013942 |
| RS08565 | -1.15463 | 0.001743 | 0.00921 | RS01575 | 0.762414 | 0.000516 | 0.003328 |
| RS21090 | -1.15323 | 2.48E-10 | 1.6E-08 | RS29175 | 0.762914 | 6.92E-05 | 0.000616 |
| RS29735 | -1.15195 | 0.000149 | 0.001156 | RS11090 | 0.765363 | 2.69E-05 | 0.000273 |
| RS07220 | -1.15191 | 1.51E-09 | 7.53E-08 | RS13035 | 0.765491 | 1.63E-06 | 2.65E-05 |
| RS24955 | -1.15045 | 5.84E-05 | 0.000533 | RS07985 | 0.766671 | 0.000295 | 0.002069 |
| RS16685 | -1.14818 | 0.00056 | 0.003555 | RS05130 | 0.768332 | 0.004589 | 0.020303 |
| RS12285 | -1.14814 | 0.017155 | 0.057924 | RS00185 | 0.769698 | 0.00599 | 0.025158 |
| RS11945 | -1.14651 | 0.000109 | 0.000886 | RS13635 | 0.770483 | 0.003488 | 0.01627 |
| RS07235 | -1.14586 | 1.31E-08 | 4.6E-07 | RS00720 | 0.771333 | 0.007564 | 0.03001 |
| RS22385 | -1.14446 | 1.16E-05 | 0.000134 | RS14910 | 0.772065 | 0.003083 | 0.014746 |
| RS07860 | -1.14018 | 2.6E-11 | 2.53E-09 | RS29295 | 0.772411 | 0.013728 | 0.048447 |
| RS16145 | -1.13509 | 0.001542 | 0.008278 | RS16860 | 0.772942 | 0.006821 | 0.027739 |
| RS18420 | -1.13308 | 0.003951 | 0.018085 | RS06385 | 0.773848 | 0.011678 | 0.042513 |
| RS28220 | -1.13017 | 0.001117 | 0.006415 | RS23175 | 0.774185 | 0.018946 | 0.062666 |
| RS21180 | -1.129 | 0.028221 | 0.085316 | RS13670 | 0.774489 | 0.013485 | 0.047782 |
| RS12355 | -1.12869 | 0.000125 | 0.000993 | RS07685 | 0.775166 | 0.004208 | 0.018963 |
| RS15605 | -1.12859 | 4.98E-05 | 0.000465 | RS16720 | 0.77568 | 0.042986 | 0.118082 |
| RS02955 | -1.12481 | 5.6E-06 | 7.36E-05 | RS26610 | 0.780254 | 0.0011 | 0.006326 |
| RS25150 | -1.12408 | 0.000634 | 0.003947 | RS13940 | 0.780362 | 0.028628 | 0.086028 |
| RS17810 | -1.12365 | 0.002258 | 0.011373 | RS26695 | 0.781073 | 0.020038 | 0.065487 |
| RS25160 | -1.12217 | 0.00023 | 0.001675 | RS11600 | 0.785575 | 0.0006 | 0.003753 |
| RS26445 | -1.12196 | 0.035148 | 0.100579 | RS19990 | 0.785626 | 1.45E-05 | 0.000162 |
| RS28420 | -1.12128 | 0.000198 | 0.001471 | RS00400 | 0.786612 | 0.016256 | 0.05549 |
| RS19155 | -1.1188 | 0.005657 | 0.024081 | RS12040 | 0.787896 | 2.42E-05 | 0.000249 |
| RS15890 | -1.11543 | 0.000772 | 0.004727 | RS18575 | 0.790018 | 2.03E-06 | 3.18E-05 |
| RS25165 | -1.11483 | 2.49E-06 | 3.83E-05 | RS07945 | 0.79036 | 0.007637 | 0.030222 |
| RS07865 | -1.10502 | 0.000155 | 0.001195 | RS03855 | 0.791896 | 0.015593 | 0.053687 |
| RS10620 | -1.10101 | 9.58E-07 | 1.68E-05 | RS04030 | 0.79381 | 1.56E-05 | 0.000172 |
| RS02765 | -1.10072 | 3.31E-05 | 0.000326 | RS31060 | 0.794192 | 0.010807 | 0.040076 |
| RS02890 | -1.1 | 4.84E-09 | 1.95E-07 | RS09820 | 0.796278 | 0.030517 | 0.090264 |
| RS13750 | -1.09669 | 5.84E-05 | 0.000533 | RS12105 | 0.796448 | 0.000373 | 0.002521 |
| RS28365 | -1.09595 | 0.010953 | 0.040515 | RS15580 | 0.799656 | 0.004264 | 0.01914 |
| RS31995 | -1.09444 | 0.001406 | 0.0077 | RS31030 | 0.800812 | 0.023981 | 0.075389 |
| RS23590 | -1.09378 | 1.37E-06 | 2.3E-05 | RS05955 | 0.803247 | 0.003438 | 0.016058 |
| RS23870 | -1.09107 | 1.45E-06 | 2.43E-05 | RS27320 | 0.803686 | 2.04E-06 | 3.18E-05 |
| RS04445 | -1.0897 | 0.005431 | 0.023299 | RS30115 | 0.803693 | 0.004385 | 0.019543 |
| RS07495 | -1.08751 | 0.001557 | 0.008349 | RS26530 | 0.804297 | 0.000229 | 0.001674 |
| RS24605 | -1.08599 | 1.48E-05 | 0.000166 | RS01115 | 0.805321 | 3.37E-06 | 4.86E-05 |
| RS06380 | -1.0813 | 1.25E-06 | 2.13E-05 | RS01835 | 0.807599 | 7.82E-07 | 1.41E-05 |
| RS13335 | -1.0777 | 0.00312 | 0.01489 | RS16345 | 0.807617 | 7.54E-07 | 1.38E-05 |
| RS27490 | -1.07739 | 0.029523 | 0.088126 | RS12035 | 0.810267 | 0.001032 | 0.005995 |
| RS10330 | -1.07678 | 4.89E-06 | 6.57E-05 | RS11750 | 0.810331 | 0.001773 | 0.009326 |
| RS16680 | -1.07448 | 0.002895 | 0.013982 | RS04170 | 0.811219 | 1.35E-05 | 0.000152 |
| RS06360 | -1.07253 | 6.04E-09 | 2.4E-07 | RS07370 | 0.813318 | 3.07E-06 | 4.49E-05 |
| RS22960 | -1.07224 | 0.000198 | 0.001471 | RS00910 | 0.813841 | 0.007514 | 0.029863 |
| RS12245 | -1.06946 | 0.001648 | 0.008755 | RS26320 | 0.815482 | 0.010003 | 0.037503 |
| RS04110 | -1.06248 | 0.001176 | 0.006673 | RS06390 | 0.815703 | 0.01001 | 0.037503 |
| RS04920 | -1.06057 | 0.000729 | 0.004483 | RS06620 | 0.81634 | 0.003984 | 0.018197 |
| RS21065 | -1.0605 | 0.002174 | 0.011024 | RS01150 | 0.816443 | 0.00023 | 0.001675 |
| RS25015 | -1.05638 | 4.15E-08 | 1.15E-06 | RS26825 | 0.816575 | 0.003928 | 0.018018 |
| RS00450 | -1.05636 | 0.000252 | 0.001802 | RS12985 | 0.817101 | 0.006611 | 0.027139 |
| RS17665 | -1.05579 | 0.001905 | 0.009822 | RS20360 | 0.817559 | 7.97E-05 | 0.000688 |
| RS13120 | -1.05151 | 7.02E-06 | 8.81E-05 | RS04590 | 0.819491 | 0.000188 | 0.001422 |
| RS12570 | -1.05085 | 0.003181 | 0.015147 | RS27940 | 0.819978 | 0.001262 | 0.007053 |
| RS07260 | -1.04948 | 3.31E-08 | 9.6E-07 | RS13455 | 0.824171 | 0.001686 | 0.00893 |
| RS21190 | -1.04906 | 0.004275 | 0.019166 | RS00690 | 0.824759 | 9.17E-05 | 0.000772 |
| RS07165 | -1.0489 | 7.7E-09 | 2.98E-07 | RS18410 | 0.825362 | 0.004251 | 0.019098 |
| RS03605 | -1.04597 | 0.000855 | 0.005144 | RS02505 | 0.826982 | 0.003508 | 0.016331 |
| RS01735 | -1.03596 | 0.002655 | 0.013011 | RS05200 | 0.827624 | 0.008778 | 0.033781 |
| RS03980 | -1.03504 | 1.65E-05 | 0.000181 | RS27700 | 0.828348 | 0.000358 | 0.002427 |
| RS26440 | -1.03446 | 1.24E-07 | 2.91E-06 | RS27580 | 0.829371 | 0.007505 | 0.029863 |
| RS21195 | -1.03414 | 5.8E-05 | 0.000532 | RS02420 | 0.83208 | 0.001448 | 0.007859 |
| RS02935 | -1.03253 | 4.35E-06 | 5.99E-05 | RS00115 | 0.835429 | 0.001402 | 0.00769 |
| RS24195 | -1.02995 | 9.74E-06 | 0.000116 | RS17915 | 0.836174 | 0.016743 | 0.056842 |
| RS22695 | -1.02297 | 1.78E-06 | 2.86E-05 | RS31040 | 0.838106 | 0.009245 | 0.035303 |
| RS27290 | -1.02016 | 0.001773 | 0.009326 | RS04875 | 0.838578 | 4.29E-08 | 1.17E-06 |
| RS07515 | -1.01951 | 2.6E-13 | 4.94E-11 | RS22110 | 0.842303 | 0.000177 | 0.001345 |
| RS25130 | -1.01783 | 6.78E-05 | 0.000604 | RS22915 | 0.843173 | 0.026078 | 0.080307 |
| RS12735 | -1.01748 | 0.023722 | 0.074788 | RS19610 | 0.844388 | 0.003286 | 0.015459 |
| RS24935 | -1.01624 | 0.001844 | 0.009628 | RS08735 | 0.84502 | 0.02577 | 0.079773 |
| RS15980 | -1.01304 | 0.000103 | 0.000844 | RS12205 | 0.846256 | 0.002511 | 0.012386 |
| RS27605 | -1.01236 | 0.00307 | 0.014696 | RS13595 | 0.848138 | 0.007104 | 0.0286 |
| RS32380 | -1.01098 | 0.000107 | 0.000876 | RS15750 | 0.848711 | 0.002565 | 0.012628 |
| RS00555 | -1.01061 | 0.001602 | 0.008573 | RS06930 | 0.850266 | 0.000782 | 0.004779 |
| RS12265 | -1.00841 | 0.038237 | 0.107399 | RS30550 | 0.855185 | 0.000128 | 0.001017 |
| RS22380 | -1.00622 | 0.001031 | 0.005995 | RS17705 | 0.857997 | 0.006946 | 0.028118 |
| RS19795 | -1.00332 | 0.000319 | 0.002218 | RS14720 | 0.858077 | 0.001898 | 0.009818 |
| RS08120 | -0.99609 | 2.17E-05 | 0.000228 | RS15195 | 0.858828 | 0.000165 | 0.001266 |
| RS05830 | -0.99326 | 0.011472 | 0.042009 | RS00650 | 0.859788 | 0.000156 | 0.001205 |
| RS03425 | -0.99264 | 6.36E-05 | 0.000571 | RS13910 | 0.859953 | 7.34E-05 | 0.000642 |
| RS32370 | -0.99162 | 0.000378 | 0.002545 | RS10640 | 0.860792 | 0.006348 | 0.026307 |
| RS03390 | -0.99055 | 8.49E-06 | 0.000103 | RS22480 | 0.860835 | 0.01818 | 0.060452 |
| RS29690 | -0.98557 | 0.009365 | 0.035697 | RS00880 | 0.863973 | 0.001251 | 0.007012 |
| RS14825 | -0.98117 | 0.001746 | 0.00921 | RS32270 | 0.864165 | 0.034352 | 0.098703 |
| RS22775 | -0.97416 | 4.48E-05 | 0.000427 | RS15720 | 0.865858 | 8.44E-05 | 0.000722 |
| RS10560 | -0.97347 | 1.39E-05 | 0.000157 | RS16800 | 0.866972 | 0.019571 | 0.064295 |
| RS02945 | -0.97127 | 2.37E-05 | 0.000246 | RS08740 | 0.867509 | 0.031671 | 0.092658 |
| RS02760 | -0.97009 | 0.001331 | 0.007362 | RS05970 | 0.872835 | 0.000907 | 0.005385 |
| RS02950 | -0.96904 | 1.05E-07 | 2.57E-06 | RS04010 | 0.877517 | 0.000913 | 0.005404 |
| RS05425 | -0.96597 | 0.000128 | 0.001017 | RS00885 | 0.878339 | 6.43E-06 | 8.13E-05 |
| RS17790 | -0.96592 | 0.031148 | 0.091601 | RS18235 | 0.880286 | 4.52E-06 | 6.17E-05 |
| RS22375 | -0.96396 | 0.000465 | 0.003035 | RS15440 | 0.881238 | 0.000105 | 0.000862 |
| RS07835 | -0.9637 | 2.98E-08 | 8.94E-07 | RS06455 | 0.882177 | 0.036628 | 0.103678 |
| RS23935 | -0.95926 | 2.23E-05 | 0.000233 | RS20215 | 0.882802 | 0.004006 | 0.01826 |
| RS04545 | -0.95843 | 0.004368 | 0.019506 | RS21100 | 0.883464 | 0.000345 | 0.002354 |
| RS24860 | -0.95683 | 0.007353 | 0.029414 | RS30075 | 0.884465 | 0.000971 | 0.005713 |
| RS25495 | -0.95668 | 0.000134 | 0.00106 | RS10400 | 0.885465 | 0.029275 | 0.087446 |
| RS04130 | -0.95611 | 9.92E-06 | 0.000118 | RS04390 | 0.88753 | 0.007956 | 0.031157 |
| RS05275 | -0.95502 | 0.00014 | 0.001098 | RS14880 | 0.887795 | 0.001027 | 0.005982 |
| RS04125 | -0.95319 | 1.87E-05 | 0.000201 | RS03715 | 0.888268 | 9.46E-05 | 0.00079 |
| RS05025 | -0.9523 | 0.003606 | 0.016715 | RS22765 | 0.890007 | 0.000138 | 0.001084 |
| RS26335 | -0.95176 | 9.26E-06 | 0.000111 | RS23435 | 0.890622 | 0.000335 | 0.002309 |
| RS08590 | -0.95122 | 3.72E-06 | 5.28E-05 | RS03865 | 0.890868 | 0.00571 | 0.02419 |
| RS20175 | -0.9495 | 0.000162 | 0.001248 | RS21580 | 0.892398 | 3.97E-06 | 5.57E-05 |
| RS20240 | -0.94466 | 0.001618 | 0.008634 | RS18585 | 0.892763 | 2.28E-05 | 0.000238 |
| RS15585 | -0.94453 | 2.43E-06 | 3.75E-05 | RS11985 | 0.895443 | 3.66E-05 | 0.000357 |
| RS16675 | -0.94294 | 5.04E-06 | 6.72E-05 | RS07950 | 0.896489 | 0.001261 | 0.007053 |
| RS23795 | -0.94257 | 2.64E-06 | 3.97E-05 | RS06895 | 0.898582 | 0.014272 | 0.050044 |
| RS08040 | -0.94139 | 0.030244 | 0.089805 | RS10345 | 0.89862 | 0.000232 | 0.001682 |
| RS23245 | -0.94053 | 1.29E-10 | 9.73E-09 | RS23305 | 0.906255 | 0.000429 | 0.00283 |
| RS07360 | -0.93898 | 0.000247 | 0.001774 | RS10055 | 0.907051 | 1.98E-05 | 0.00021 |
| RS02310 | -0.93887 | 0.003287 | 0.015459 | RS02335 | 0.908256 | 9.77E-05 | 0.00081 |
| RS11380 | -0.93827 | 0.030835 | 0.090857 | RS00685 | 0.908656 | 1.06E-05 | 0.000125 |
| RS28285 | -0.93801 | 0.000221 | 0.001623 | RS20905 | 0.912126 | 0.00122 | 0.006851 |
| RS03145 | -0.9361 | 1.26E-07 | 2.92E-06 | RS18105 | 0.912637 | 7.24E-05 | 0.000637 |
| RS29715 | -0.93112 | 0.002714 | 0.013254 | RS29605 | 0.913756 | 0.029795 | 0.088697 |
| RS06480 | -0.92865 | 0.015216 | 0.052637 | RS30340 | 0.914791 | 0.008785 | 0.033781 |
| RS13935 | -0.9271 | 5.32E-06 | 7.03E-05 | RS17595 | 0.915254 | 0.000478 | 0.003108 |
| RS02805 | -0.92386 | 0.000554 | 0.003522 | RS18085 | 0.917128 | 0.000256 | 0.001831 |
| RS02810 | -0.92183 | 5.86E-06 | 7.59E-05 | RS14755 | 0.917527 | 0.012246 | 0.044216 |
| RS02940 | -0.91779 | 1.95E-06 | 3.09E-05 | RS23850 | 0.91833 | 0.003591 | 0.016663 |
| RS00710 | -0.91575 | 0.000324 | 0.002251 | RS01125 | 0.920419 | 8.18E-06 | 0.0001 |
| RS22845 | -0.91407 | 2.86E-05 | 0.000289 | RS20970 | 0.921364 | 0.000463 | 0.003029 |
| RS25275 | -0.91379 | 6E-05 | 0.000543 | RS06910 | 0.921428 | 4.55E-06 | 6.18E-05 |
| RS31965 | -0.9121 | 8.4E-05 | 0.000719 | RS05115 | 0.922144 | 0.000678 | 0.004204 |
| RS19450 | -0.90955 | 0.001906 | 0.009822 | RS32200 | 0.925115 | 0.014154 | 0.049718 |
| RS13015 | -0.90679 | 0.008129 | 0.031745 | RS11620 | 0.925197 | 0.000603 | 0.003769 |
| RS25480 | -0.90403 | 3.21E-05 | 0.000318 | RS24060 | 0.925278 | 9.19E-06 | 0.000111 |
| RS23000 | -0.90379 | 0.00163 | 0.008682 | RS03330 | 0.925349 | 3.3E-06 | 4.8E-05 |
| RS16295 | -0.90146 | 0.003946 | 0.018083 | RS14990 | 0.925365 | 6.15E-07 | 1.16E-05 |
| RS32350 | -0.90036 | 0.000595 | 0.003731 | RS04600 | 0.926291 | 0.027317 | 0.083361 |
| RS07150 | -0.90036 | 3.57E-06 | 5.1E-05 | RS04025 | 0.926421 | 3.05E-05 | 0.000304 |
| RS14090 | -0.89943 | 0.000194 | 0.001448 | RS26970 | 0.92934 | 0.004906 | 0.021492 |
| RS31960 | -0.89778 | 9.07E-06 | 0.00011 | RS14520 | 0.930214 | 0.013194 | 0.046902 |
| RS32035 | -0.8977 | 0.000109 | 0.000886 | RS28430 | 0.930555 | 2.79E-09 | 1.23E-07 |
| RS16450 | -0.89765 | 2.48E-05 | 0.000255 | RS26650 | 0.933535 | 1.18E-07 | 2.81E-06 |
| RS20190 | -0.89376 | 0.006224 | 0.025891 | RS12905 | 0.942008 | 0.006473 | 0.02667 |
| RS10830 | -0.89192 | 0.003046 | 0.014633 | RS27875 | 0.942031 | 0.002294 | 0.011493 |
| RS05455 | -0.89159 | 2.94E-05 | 0.000294 | RS08290 | 0.942627 | 4.2E-05 | 0.000404 |
| RS00855 | -0.8904 | 0.014713 | 0.051138 | RS08035 | 0.943099 | 0.00619 | 0.025797 |
| RS25490 | -0.88929 | 4.96E-05 | 0.000464 | RS19615 | 0.944108 | 2.68E-05 | 0.000273 |
| RS27500 | -0.88852 | 0.018882 | 0.062503 | RS25900 | 0.946321 | 2.49E-13 | 4.94E-11 |
| RS05640 | -0.88563 | 0.001099 | 0.006326 | RS04790 | 0.948053 | 1.54E-06 | 2.54E-05 |
| RS20260 | -0.88506 | 0.027498 | 0.083737 | RS20435 | 0.949073 | 0.001171 | 0.006662 |
| RS12565 | -0.88504 | 0.03041 | 0.090038 | RS16920 | 0.952449 | 0.002733 | 0.013331 |
| RS17165 | -0.88462 | 0.00434 | 0.019418 | RS04475 | 0.952775 | 0.002506 | 0.012378 |
| RS27445 | -0.88455 | 0.00042 | 0.002784 | RS04150 | 0.957176 | 0.000136 | 0.001069 |
| RS05385 | -0.88262 | 3.83E-05 | 0.000371 | RS24885 | 0.958389 | 0.022263 | 0.070802 |
| RS07160 | -0.88207 | 1.12E-05 | 0.000131 | RS07095 | 0.958954 | 0.000562 | 0.003561 |
| RS25735 | -0.8812 | 2.41E-05 | 0.000248 | RS30195 | 0.960438 | 2.3E-05 | 0.00024 |
| RS08280 | -0.88011 | 5.32E-07 | 1.04E-05 | RS02960 | 0.963283 | 0.015954 | 0.054585 |
| RS25690 | -0.87808 | 0.009594 | 0.036309 | RS21105 | 0.96427 | 2.34E-05 | 0.000243 |
| RS15415 | -0.87764 | 0.005296 | 0.022833 | RS19960 | 0.965744 | 0.006527 | 0.026843 |
| RS21365 | -0.87572 | 0.00689 | 0.027943 | RS22365 | 0.966389 | 0.008967 | 0.034332 |
| RS07760 | -0.87546 | 6.02E-06 | 7.71E-05 | RS05735 | 0.967388 | 1.22E-06 | 2.08E-05 |
| RS07800 | -0.87476 | 8.77E-05 | 0.000745 | RS31705 | 0.968191 | 0.005181 | 0.022448 |
| RS12360 | -0.86807 | 0.001286 | 0.007148 | RS31235 | 0.968463 | 0.000138 | 0.001084 |
| RS18860 | -0.86613 | 0.007717 | 0.030477 | RS08055 | 0.969628 | 7.78E-05 | 0.000675 |
| RS13790 | -0.8619 | 1.05E-05 | 0.000124 | RS05590 | 0.969989 | 0.0005 | 0.003229 |
| RS08020 | -0.86108 | 0.005219 | 0.022567 | RS22550 | 0.970192 | 0.001001 | 0.005855 |
| RS25410 | -0.85939 | 9.64E-07 | 1.68E-05 | RS06800 | 0.971722 | 0.021441 | 0.068773 |
| RS04135 | -0.85747 | 0.000298 | 0.002087 | RS24845 | 0.975583 | 0.033699 | 0.097402 |
| RS22495 | -0.85727 | 0.025387 | 0.078958 | RS18980 | 0.976609 | 1.97E-08 | 6.52E-07 |
| RS00975 | -0.85651 | 0.010443 | 0.038992 | RS15290 | 0.979318 | 0.001585 | 0.008491 |
| RS16305 | -0.85584 | 0.004035 | 0.018374 | RS14060 | 0.979352 | 0.003231 | 0.015319 |
| RS07795 | -0.85341 | 0.000103 | 0.000844 | RS31130 | 0.980766 | 1.94E-08 | 6.48E-07 |
| RS08070 | -0.85316 | 0.000133 | 0.001053 | RS02290 | 0.980986 | 8.1E-06 | 9.96E-05 |
| RS13580 | -0.85119 | 0.01833 | 0.060858 | RS09005 | 0.982185 | 0.006919 | 0.028033 |
| RS28620 | -0.84979 | 1.27E-05 | 0.000145 | RS26385 | 0.982869 | 0.000799 | 0.004852 |
| RS07265 | -0.84828 | 4.05E-08 | 1.14E-06 | RS22555 | 0.985178 | 0.000199 | 0.001471 |
| RS31120 | -0.84677 | 0.019695 | 0.064605 | RS17100 | 0.986135 | 0.002002 | 0.010249 |
| RS25740 | -0.84657 | 4.93E-06 | 6.59E-05 | RS07680 | 0.988598 | 0.003187 | 0.015158 |
| RS22340 | -0.84482 | 0.006698 | 0.027344 | RS05805 | 0.990491 | 0.038452 | 0.107931 |
| RS04435 | -0.84418 | 0.002933 | 0.014134 | RS31320 | 0.991206 | 0.021127 | 0.068029 |
| RS00615 | -0.84373 | 0.002444 | 0.012114 | RS22830 | 0.991256 | 1.07E-09 | 5.69E-08 |
| RS11580 | -0.84263 | 3.17E-06 | 4.63E-05 | RS04795 | 0.992963 | 0.00796 | 0.031157 |
| RS24585 | -0.84046 | 0.000283 | 0.001993 | RS24170 | 0.993263 | 0.035069 | 0.100432 |
| RS27385 | -0.84025 | 7.64E-07 | 1.39E-05 | RS07085 | 0.994409 | 0.000143 | 0.001117 |
| RS25230 | -0.83952 | 9.3E-05 | 0.000781 | RS26975 | 0.994549 | 0.008881 | 0.034061 |
| RS24900 | -0.8393 | 0.003268 | 0.015455 | RS01860 | 0.996123 | 0.000407 | 0.00272 |
| RS02120 | -0.83764 | 0.033602 | 0.097185 | RS26270 | 0.997647 | 0.001631 | 0.008682 |
| RS04510 | -0.83701 | 0.014512 | 0.050601 | RS09945 | 0.997719 | 0.044927 | 0.122263 |
| RS08140 | -0.83695 | 5.98E-06 | 7.69E-05 | RS04350 | 0.999231 | 0.001461 | 0.00791 |
| RS32365 | -0.83631 | 0.002146 | 0.0109 | RS19060 | 0.999577 | 0.021091 | 0.067962 |
| RS22635 | -0.83617 | 0.01202 | 0.043506 | RS11505 | 0.999992 | 0.001667 | 0.008841 |
| RS17575 | -0.83555 | 0.003371 | 0.015776 | RS07000 | 1.002283 | 0.000493 | 0.003188 |
| RS05420 | -0.83218 | 6.03E-06 | 7.71E-05 | RS06880 | 1.006789 | 0.001151 | 0.006567 |
| RS23345 | -0.82796 | 0.000586 | 0.003691 | RS23255 | 1.007193 | 3.71E-07 | 7.63E-06 |
| RS25540 | -0.8272 | 0.006128 | 0.025628 | RS09010 | 1.007703 | 8.07E-07 | 1.45E-05 |
| RS30035 | -0.82591 | 0.016875 | 0.0572 | RS00120 | 1.007845 | 3.04E-06 | 4.46E-05 |
| RS09615 | -0.82348 | 5.31E-05 | 0.000491 | RS03915 | 1.008539 | 0.020626 | 0.067057 |
| RS18830 | -0.82304 | 0.006087 | 0.02549 | RS04235 | 1.009082 | 7.95E-05 | 0.000687 |
| RS25765 | -0.82221 | 2.12E-09 | 9.98E-08 | RS21690 | 1.009255 | 0.005851 | 0.024691 |
| RS03090 | -0.82174 | 0.000985 | 0.005776 | RS05155 | 1.012597 | 0.000744 | 0.004558 |
| RS21420 | -0.82066 | 0.000221 | 0.001623 | RS04800 | 1.016947 | 0.000227 | 0.001659 |
| RS04460 | -0.81731 | 0.01157 | 0.04219 | RS17590 | 1.024002 | 0.001829 | 0.009566 |
| RS11625 | -0.81585 | 1.23E-07 | 2.9E-06 | RS29480 | 1.024709 | 0.007878 | 0.030918 |
| RS25755 | -0.81576 | 1.84E-06 | 2.95E-05 | RS07105 | 1.025342 | 0.012346 | 0.044465 |
| RS14125 | -0.81479 | 0.00778 | 0.03056 | RS00070 | 1.029924 | 0.000171 | 0.001305 |
| RS30060 | -0.80656 | 8.73E-05 | 0.000744 | RS30680 | 1.031385 | 0.003419 | 0.015986 |
| RS15155 | -0.80558 | 0.012338 | 0.044465 | RS09975 | 1.034231 | 0.042061 | 0.116124 |
| RS26765 | -0.80514 | 0.01314 | 0.046785 | RS00345 | 1.035463 | 0.000399 | 0.002677 |
| RS08200 | -0.8013 | 0.015506 | 0.053428 | RS05815 | 1.036424 | 6.99E-05 | 0.00062 |
| RS02815 | -0.79968 | 0.000591 | 0.003711 | RS23500 | 1.039307 | 4.74E-05 | 0.000446 |
| RS22880 | -0.79936 | 0.001268 | 0.007062 | RS04175 | 1.040286 | 8.96E-05 | 0.00076 |
| RS07925 | -0.79888 | 0.00739 | 0.029509 | RS04385 | 1.041935 | 0.001099 | 0.006326 |
| RS06340 | -0.79818 | 0.005886 | 0.024816 | RS29250 | 1.048224 | 0.001004 | 0.005865 |
| RS21715 | -0.79793 | 1.13E-05 | 0.000131 | RS16550 | 1.048546 | 3.99E-06 | 5.57E-05 |
| RS22905 | -0.7978 | 0.043842 | 0.119983 | RS20570 | 1.050866 | 0.000691 | 0.004271 |
| RS26855 | -0.79777 | 0.002279 | 0.011452 | RS05670 | 1.051681 | 6.34E-06 | 8.09E-05 |
| RS08580 | -0.79684 | 0.007027 | 0.028395 | RS07080 | 1.05176 | 0.00134 | 0.007398 |
| RS08475 | -0.79563 | 0.001518 | 0.008169 | RS09080 | 1.05249 | 0.031787 | 0.092757 |
| RS09190 | -0.79552 | 0.006215 | 0.025877 | RS26820 | 1.066648 | 1.25E-05 | 0.000143 |
| RS23705 | -0.7928 | 0.001445 | 0.007857 | RS26810 | 1.06916 | 9.7E-05 | 0.000807 |
| RS15260 | -0.79123 | 7.22E-06 | 9.01E-05 | RS02500 | 1.069941 | 0.007272 | 0.02914 |
| RS25500 | -0.78838 | 0.029133 | 0.087139 | RS22545 | 1.072265 | 0.003053 | 0.014633 |
| RS02330 | -0.78754 | 3.62E-05 | 0.000355 | RS05545 | 1.072425 | 0.00285 | 0.01382 |
| RS28140 | -0.78625 | 0.000168 | 0.001277 | RS21095 | 1.072685 | 0.001984 | 0.010178 |
| RS00805 | -0.7862 | 0.036773 | 0.10393 | RS01770 | 1.072756 | 2.5E-08 | 8.04E-07 |
| RS14960 | -0.78417 | 4.05E-06 | 5.65E-05 | RS26255 | 1.076857 | 2.89E-06 | 4.27E-05 |
| RS15590 | -0.78396 | 6.3E-05 | 0.000567 | RS05740 | 1.080583 | 1.42E-10 | 1.04E-08 |
| RS23930 | -0.78344 | 0.000143 | 0.00112 | RS02025 | 1.083618 | 0.004224 | 0.019018 |
| RS25245 | -0.78281 | 0.004199 | 0.018963 | RS06900 | 1.083729 | 0.016934 | 0.057268 |
| RS29630 | -0.78005 | 0.00066 | 0.0041 | RS14145 | 1.085169 | 0.037812 | 0.106272 |
| RS23325 | -0.77972 | 0.02595 | 0.080083 | RS20340 | 1.086601 | 0.000128 | 0.001015 |
| RS15250 | -0.77872 | 5.25E-06 | 6.99E-05 | RS04565 | 1.08702 | 6.56E-09 | 2.59E-07 |
| RS18050 | -0.77857 | 2.14E-05 | 0.000226 | RS15695 | 1.093 | 0.000327 | 0.002264 |
| RS23515 | -0.77851 | 0.00018 | 0.001361 | RS00475 | 1.093222 | 0.001398 | 0.007678 |
| RS29485 | -0.77815 | 0.029967 | 0.089086 | RS01170 | 1.093715 | 0.001902 | 0.009822 |
| RS23080 | -0.77708 | 4.89E-06 | 6.57E-05 | RS00350 | 1.094143 | 0.011301 | 0.041522 |
| RS05380 | -0.77704 | 0.000358 | 0.002427 | RS00105 | 1.094692 | 0.000251 | 0.001798 |
| RS24065 | -0.7759 | 0.03544 | 0.101032 | RS31240 | 1.09709 | 0.00087 | 0.005209 |
| RS23075 | -0.77567 | 4.58E-05 | 0.000434 | RS06885 | 1.0991 | 1.86E-05 | 0.0002 |
| RS12075 | -0.77343 | 0.04243 | 0.116848 | RS17055 | 1.099489 | 0.005054 | 0.022029 |
| RS25515 | -0.77305 | 0.016917 | 0.057254 | RS27540 | 1.09988 | 4.42E-05 | 0.000422 |
| RS30205 | -0.76982 | 5.99E-05 | 0.000543 | RS05745 | 1.103157 | 1.09E-05 | 0.000128 |
| RS28555 | -0.76891 | 0.032293 | 0.093898 | RS05135 | 1.104177 | 0.000374 | 0.002521 |
| RS02635 | -0.76676 | 0.000275 | 0.001954 | RS15380 | 1.106749 | 0.002035 | 0.010403 |
| RS23495 | -0.76524 | 0.006784 | 0.027641 | RS11055 | 1.106937 | 1.69E-05 | 0.000184 |
| RS16480 | -0.76461 | 0.028461 | 0.085777 | RS21950 | 1.111628 | 0.000427 | 0.002821 |
| RS14700 | -0.76301 | 0.019896 | 0.065219 | RS17455 | 1.113004 | 1.69E-05 | 0.000184 |
| RS17000 | -0.76293 | 0.031719 | 0.092658 | RS06760 | 1.113791 | 1.45E-06 | 2.43E-05 |
| RS00770 | -0.76288 | 0.01687 | 0.0572 | RS09000 | 1.114991 | 0.015491 | 0.053419 |
| RS17995 | -0.76014 | 0.042186 | 0.116324 | RS00540 | 1.116579 | 6.01E-07 | 1.15E-05 |
| RS20255 | -0.75809 | 0.028183 | 0.085316 | RS30675 | 1.118108 | 0.046251 | 0.124857 |
| RS31500 | -0.75797 | 4.71E-05 | 0.000445 | RS29320 | 1.118332 | 2.61E-06 | 3.96E-05 |
| RS22585 | -0.75796 | 0.003771 | 0.017335 | RS02130 | 1.11928 | 2.66E-06 | 3.99E-05 |
| RS30000 | -0.75712 | 0.002223 | 0.011218 | RS31695 | 1.125034 | 2.7E-05 | 0.000274 |
| RS23465 | -0.75695 | 8.83E-11 | 7.16E-09 | RS05950 | 1.129738 | 6.3E-07 | 1.18E-05 |
| RS26290 | -0.75316 | 0.007527 | 0.02989 | RS02560 | 1.13632 | 5.69E-07 | 1.1E-05 |
| RS18400 | -0.74937 | 0.002296 | 0.011493 | RS15320 | 1.138423 | 1.19E-05 | 0.000137 |
| RS09160 | -0.74908 | 0.00274 | 0.013338 | RS15790 | 1.144707 | 2.28E-07 | 5.06E-06 |
| RS03140 | -0.74892 | 1.55E-06 | 2.55E-05 | RS21530 | 1.146053 | 0.000223 | 0.001633 |
| RS30175 | -0.74663 | 0.014671 | 0.051033 | RS03740 | 1.146873 | 4.65E-07 | 9.25E-06 |
| RS21135 | -0.74606 | 1.77E-05 | 0.000192 | RS28235 | 1.150263 | 0.000631 | 0.003931 |
| RS24940 | -0.74583 | 0.002619 | 0.012861 | RS17220 | 1.152109 | 0.000441 | 0.002909 |
| RS21255 | -0.74364 | 0.00054 | 0.003454 | RS30885 | 1.153268 | 0.004056 | 0.018448 |
| RS01825 | -0.74146 | 0.000545 | 0.003481 | RS17415 | 1.154613 | 0.00712 | 0.028601 |
| RS06020 | -0.74123 | 0.017497 | 0.058963 | RS06975 | 1.155891 | 0.001174 | 0.00667 |
| RS03880 | -0.74114 | 0.009502 | 0.036095 | RS02345 | 1.156235 | 7.38E-05 | 0.000642 |
| RS08015 | -0.73928 | 0.000192 | 0.001445 | RS26080 | 1.15675 | 0.006261 | 0.025969 |
| RS22010 | -0.73927 | 0.001183 | 0.006695 | RS11110 | 1.16074 | 2.79E-06 | 4.16E-05 |
| RS31855 | -0.7368 | 0.002895 | 0.013982 | RS30805 | 1.161579 | 0.011333 | 0.041606 |
| RS10880 | -0.7365 | 0.013191 | 0.046902 | RS32045 | 1.162529 | 0.000532 | 0.003416 |
| RS27400 | -0.73644 | 0.003097 | 0.014796 | RS26050 | 1.165998 | 0.004852 | 0.021298 |
| RS08405 | -0.73626 | 0.004991 | 0.021776 | RS04840 | 1.166841 | 1.47E-05 | 0.000164 |
| RS31605 | -0.73427 | 0.01529 | 0.052852 | RS30210 | 1.167325 | 3.96E-06 | 5.57E-05 |
| RS25600 | -0.73399 | 3.51E-05 | 0.000344 | RS12680 | 1.170874 | 4.07E-06 | 5.66E-05 |
| RS19715 | -0.73267 | 0.002202 | 0.011128 | RS20430 | 1.172007 | 9.74E-05 | 0.000808 |
| RS18385 | -0.73035 | 0.00057 | 0.003604 | RS05375 | 1.184714 | 0.020293 | 0.066222 |
| RS03705 | -0.73027 | 7.37E-05 | 0.000642 | RS04535 | 1.186286 | 5.89E-05 | 0.000536 |
| RS16130 | -0.72807 | 5.42E-05 | 0.0005 | RS06890 | 1.189353 | 6.73E-05 | 0.000602 |
| RS07635 | -0.72567 | 0.008735 | 0.033647 | RS12130 | 1.19039 | 4.64E-08 | 1.25E-06 |
| RS16110 | -0.72318 | 0.008418 | 0.032569 | RS12140 | 1.200178 | 4.87E-05 | 0.000458 |
| RS20985 | -0.72136 | 0.000253 | 0.001812 | RS07005 | 1.203257 | 3.75E-15 | 1.37E-12 |
| RS08145 | -0.71955 | 7.7E-06 | 9.55E-05 | RS23370 | 1.208186 | 2.73E-06 | 4.07E-05 |
| RS25395 | -0.71899 | 0.001915 | 0.009858 | RS24510 | 1.212423 | 3.5E-06 | 5.02E-05 |
| RS06255 | -0.71767 | 0.031923 | 0.093007 | RS09850 | 1.212526 | 5.74E-06 | 7.46E-05 |
| RS13155 | -0.71724 | 0.002062 | 0.010531 | RS09780 | 1.215285 | 0.001141 | 0.006521 |
| RS13095 | -0.71686 | 0.011166 | 0.041129 | RS20840 | 1.220192 | 0.000278 | 0.001967 |
| RS26230 | -0.71658 | 4.72E-05 | 0.000445 | RS29890 | 1.220217 | 0.001886 | 0.009788 |
| RS05005 | -0.71408 | 0.000308 | 0.002144 | RS14240 | 1.222216 | 3.89E-06 | 5.5E-05 |
| RS15050 | -0.71365 | 0.005171 | 0.022447 | RS15400 | 1.223887 | 0.000109 | 0.000889 |
| RS24470 | -0.71294 | 0.001078 | 0.006229 | RS27620 | 1.226081 | 0.000144 | 0.001123 |
| RS13150 | -0.71157 | 0.000989 | 0.005795 | RS30325 | 1.230975 | 6.74E-05 | 0.000602 |
| RS22315 | -0.71111 | 0.002949 | 0.014199 | RS14070 | 1.231509 | 4.59E-05 | 0.000434 |
| RS27610 | -0.70856 | 0.041205 | 0.114236 | RS20655 | 1.231851 | 0.000209 | 0.001543 |
| RS04245 | -0.70819 | 0.000193 | 0.001447 | RS18130 | 1.2322 | 2.69E-08 | 8.35E-07 |
| RS04450 | -0.70741 | 0.029873 | 0.088869 | RS14595 | 1.232792 | 0.026352 | 0.080924 |
| RS07400 | -0.70738 | 3.18E-11 | 3.03E-09 | RS00480 | 1.232968 | 3.65E-05 | 0.000356 |
| RS07615 | -0.70642 | 0.021933 | 0.069955 | RS26815 | 1.234326 | 0.002391 | 0.011918 |
| RS11550 | -0.70637 | 0.027782 | 0.084485 | RS09985 | 1.234831 | 0.000194 | 0.001448 |
| RS04970 | -0.70578 | 0.003496 | 0.01629 | RS06985 | 1.237739 | 4.71E-06 | 6.38E-05 |
| RS07175 | -0.70384 | 0.016362 | 0.055763 | RS19425 | 1.239546 | 0.026487 | 0.08128 |
| RS15800 | -0.70087 | 0.012802 | 0.045807 | RS21855 | 1.241067 | 2.02E-06 | 3.18E-05 |
| RS19915 | -0.69867 | 0.015915 | 0.054496 | RS06960 | 1.243211 | 1.66E-11 | 1.69E-09 |
| RS23790 | -0.69678 | 0.00086 | 0.005172 | RS12135 | 1.25017 | 2.55E-07 | 5.56E-06 |
| RS07310 | -0.69645 | 0.019963 | 0.065389 | RS04530 | 1.250208 | 1.87E-06 | 2.98E-05 |
| RS18205 | -0.69614 | 2.9E-05 | 0.000291 | RS06585 | 1.251738 | 0.012634 | 0.04539 |
| RS31615 | -0.69426 | 0.003753 | 0.017308 | RS20910 | 1.253197 | 0.000877 | 0.005245 |
| RS27650 | -0.69384 | 0.047221 | 0.126929 | RS06980 | 1.254251 | 8.06E-05 | 0.000693 |
| RS07510 | -0.69303 | 0.000338 | 0.002322 | RS11705 | 1.254533 | 7.19E-07 | 1.34E-05 |
| RS00600 | -0.69235 | 0.000848 | 0.005112 | RS12425 | 1.256566 | 0.000956 | 0.005631 |
| RS30580 | -0.69085 | 0.000115 | 0.000931 | RS17580 | 1.258123 | 0.000382 | 0.002566 |
| RS07405 | -0.69011 | 0.003264 | 0.015455 | RS04240 | 1.258682 | 0.00011 | 0.000893 |
| RS25795 | -0.68913 | 0.007391 | 0.029509 | RS26005 | 1.265733 | 0.000243 | 0.001755 |
| RS25730 | -0.6886 | 0.004625 | 0.020441 | RS07115 | 1.267269 | 7.17E-10 | 4.07E-08 |
| RS22300 | -0.6872 | 0.005181 | 0.022448 | RS06165 | 1.273519 | 0.000881 | 0.005253 |
| RS17985 | -0.68668 | 0.020587 | 0.067032 | RS30590 | 1.276239 | 1.16E-07 | 2.8E-06 |
| RS15970 | -0.68635 | 0.005696 | 0.024158 | RS17510 | 1.280193 | 0.001068 | 0.006181 |
| RS23025 | -0.68511 | 0.001463 | 0.007912 | RS00195 | 1.282198 | 0.004144 | 0.018774 |
| RS08250 | -0.68335 | 0.014 | 0.049287 | RS17935 | 1.29327 | 0.000303 | 0.002119 |
| RS18255 | -0.68234 | 0.00671 | 0.027363 | RS07110 | 1.300449 | 5.05E-07 | 9.95E-06 |
| RS05000 | -0.68191 | 0.000455 | 0.002988 | RS06075 | 1.301726 | 2.93E-05 | 0.000294 |
| RS20675 | -0.68131 | 0.008159 | 0.03182 | RS21825 | 1.303519 | 0.004458 | 0.019806 |
| RS18245 | -0.68103 | 0.046868 | 0.126133 | RS02160 | 1.306227 | 0.001815 | 0.009527 |
| RS10760 | -0.67968 | 0.031241 | 0.091815 | RS09845 | 1.31592 | 2.03E-06 | 3.18E-05 |
| RS07470 | -0.67948 | 0.000155 | 0.001199 | RS24255 | 1.319341 | 4.37E-08 | 1.19E-06 |
| RS13755 | -0.67894 | 0.031975 | 0.093097 | RS22130 | 1.326995 | 0.000899 | 0.005345 |
| RS14150 | -0.67885 | 0.020926 | 0.067681 | RS05975 | 1.327859 | 0.001268 | 0.007062 |
| RS05415 | -0.67853 | 0.003193 | 0.015173 | RS20965 | 1.330163 | 0.000102 | 0.000839 |
| RS22800 | -0.67848 | 0.002814 | 0.013681 | RS18160 | 1.333556 | 1.67E-07 | 3.82E-06 |
| RS13080 | -0.67776 | 2.57E-05 | 0.000263 | RS24265 | 1.334355 | 8.68E-07 | 1.53E-05 |
| RS09725 | -0.67643 | 0.001504 | 0.008103 | RS26875 | 1.334427 | 0.003038 | 0.014607 |
| RS30225 | -0.67483 | 0.001203 | 0.00679 | RS00645 | 1.33659 | 1.59E-06 | 2.6E-05 |
| RS15030 | -0.67318 | 0.032997 | 0.095753 | RS08670 | 1.340189 | 3.31E-05 | 0.000326 |
| RS29860 | -0.67217 | 0.012677 | 0.045509 | RS14975 | 1.342999 | 3.51E-07 | 7.39E-06 |
| RS07465 | -0.67142 | 0.006678 | 0.027313 | RS12665 | 1.343439 | 0.001425 | 0.007792 |
| RS23630 | -0.67051 | 0.012887 | 0.046034 | RS28210 | 1.349635 | 8.26E-07 | 1.47E-05 |
| RS16310 | -0.67026 | 0.031816 | 0.092757 | RS24330 | 1.352675 | 0.00153 | 0.008223 |
| RS22325 | -0.66794 | 0.005669 | 0.024108 | RS19885 | 1.364201 | 0.000696 | 0.004298 |
| RS26590 | -0.66697 | 0.001947 | 0.010011 | RS17585 | 1.364834 | 2.94E-05 | 0.000294 |
| RS30995 | -0.66556 | 0.000426 | 0.002819 | RS10020 | 1.37029 | 4.16E-06 | 5.75E-05 |
| RS11170 | -0.66556 | 0.0004 | 0.002683 | RS22170 | 1.372272 | 0.005504 | 0.023498 |
| RS25315 | -0.6654 | 0.000345 | 0.002354 | RS06185 | 1.373402 | 0.000281 | 0.001983 |
| RS21425 | -0.6629 | 0.000334 | 0.002302 | RS32175 | 1.374067 | 4.53E-07 | 9.06E-06 |
| RS07785 | -0.66263 | 0.00183 | 0.009566 | RS17255 | 1.37476 | 0.000239 | 0.001724 |
| RS29835 | -0.65377 | 0.014314 | 0.050072 | RS02600 | 1.38646 | 0.003669 | 0.016972 |
| RS24795 | -0.65352 | 4.24E-05 | 0.000408 | RS08895 | 1.393026 | 0.034397 | 0.098767 |
| RS27380 | -0.65206 | 0.000974 | 0.005719 | RS14895 | 1.395355 | 0.000401 | 0.002685 |
| RS07155 | -0.64932 | 0.000417 | 0.002769 | RS14075 | 1.395427 | 0.009347 | 0.03566 |
| RS25625 | -0.64882 | 0.026071 | 0.080307 | RS00485 | 1.407377 | 4.48E-07 | 9E-06 |
| RS21595 | -0.64823 | 0.036862 | 0.10407 | RS04320 | 1.407888 | 1.41E-05 | 0.000159 |
| RS09785 | -0.64796 | 0.014481 | 0.050535 | RS13965 | 1.411339 | 9.78E-10 | 5.35E-08 |
| RS31350 | -0.64753 | 0.001224 | 0.006869 | RS09910 | 1.414189 | 7.2E-08 | 1.84E-06 |
| RS28645 | -0.64729 | 0.009422 | 0.035852 | RS21110 | 1.414739 | 0.000933 | 0.005512 |
| RS26175 | -0.64551 | 0.001287 | 0.007148 | RS04315 | 1.422484 | 0.0002 | 0.001477 |
| RS25360 | -0.64448 | 0.043648 | 0.119526 | RS19845 | 1.425693 | 0.007763 | 0.030552 |
| RS13495 | -0.6434 | 0.018097 | 0.060326 | RS09980 | 1.432499 | 4.15E-05 | 0.000401 |
| RS21505 | -0.64273 | 0.033942 | 0.097781 | RS02530 | 1.434851 | 8.18E-10 | 4.53E-08 |
| RS18810 | -0.641 | 0.045643 | 0.123722 | RS06235 | 1.435752 | 2.07E-07 | 4.62E-06 |
| RS11175 | -0.64017 | 0.005488 | 0.023454 | RS02535 | 1.437349 | 1.35E-09 | 6.87E-08 |
| RS27415 | -0.63896 | 0.010163 | 0.03801 | RS02520 | 1.43957 | 1.67E-06 | 2.71E-05 |
| RS00725 | -0.63871 | 0.005414 | 0.023252 | RS16810 | 1.440565 | 2.77E-05 | 0.00028 |
| RS31865 | -0.63832 | 9.5E-05 | 0.000792 | RS18985 | 1.44447 | 9.93E-10 | 5.37E-08 |
| RS06710 | -0.63812 | 8.1E-05 | 0.000695 | RS29315 | 1.444642 | 6.39E-08 | 1.64E-06 |
| RS24965 | -0.63533 | 0.000115 | 0.000931 | RS11650 | 1.448783 | 7.25E-05 | 0.000637 |
| RS22405 | -0.63362 | 0.010503 | 0.039184 | RS30500 | 1.455682 | 3.18E-08 | 9.42E-07 |
| RS28765 | -0.63331 | 0.028201 | 0.085316 | RS10455 | 1.457785 | 2.62E-06 | 3.96E-05 |
| RS31465 | -0.63262 | 0.004759 | 0.020974 | RS09815 | 1.458955 | 2.51E-05 | 0.000257 |
| RS31115 | -0.63176 | 0.004391 | 0.019549 | RS19385 | 1.459158 | 0.000118 | 0.000948 |
| RS01875 | -0.631 | 0.006678 | 0.027313 | RS06950 | 1.459414 | 9.88E-08 | 2.46E-06 |
| RS32090 | -0.63023 | 0.006246 | 0.025955 | RS02590 | 1.463462 | 0.00012 | 0.000963 |
| RS20230 | -0.62998 | 0.012163 | 0.043961 | RS19995 | 1.471891 | 2.44E-09 | 1.13E-07 |
| RS25180 | -0.62992 | 0.013568 | 0.047957 | RS29180 | 1.47556 | 7.88E-09 | 3E-07 |
| RS04740 | -0.62953 | 0.007729 | 0.030497 | RS11130 | 1.482911 | 9.65E-08 | 2.43E-06 |
| RS08155 | -0.62621 | 0.000357 | 0.002427 | RS11470 | 1.485555 | 3.77E-06 | 5.34E-05 |
| RS01910 | -0.62584 | 0.041611 | 0.115102 | RS32030 | 1.490741 | 2.68E-07 | 5.8E-06 |
| RS22320 | -0.6242 | 0.002269 | 0.011415 | RS17250 | 1.491126 | 0.000455 | 0.002988 |
| RS10855 | -0.62391 | 0.000819 | 0.004965 | RS23760 | 1.491914 | 3.54E-07 | 7.42E-06 |
| RS10975 | -0.62338 | 0.002912 | 0.014047 | RS29290 | 1.492016 | 1.69E-10 | 1.21E-08 |
| RS24970 | -0.62289 | 0.000566 | 0.003584 | RS14905 | 1.492161 | 1.98E-07 | 4.48E-06 |
| RS01245 | -0.622 | 0.044893 | 0.122247 | RS18590 | 1.495928 | 3.44E-13 | 6.02E-11 |
| RS02010 | -0.62175 | 0.000949 | 0.005594 | RS00335 | 1.496869 | 0.019476 | 0.064032 |
| RS08030 | -0.62132 | 0.036165 | 0.102565 | RS20915 | 1.503839 | 6.51E-12 | 7.31E-10 |
| RS13175 | -0.62117 | 0.001643 | 0.008735 | RS30935 | 1.504487 | 1.18E-05 | 0.000136 |
| RS10890 | -0.62025 | 0.008381 | 0.032457 | RS21275 | 1.505237 | 2.1E-05 | 0.000222 |
| RS29875 | -0.62025 | 0.000163 | 0.001249 | RS01140 | 1.506901 | 0.000586 | 0.003691 |
| RS17795 | -0.61917 | 0.015095 | 0.05234 | RS02585 | 1.508556 | 3.2E-05 | 0.000318 |
| RS18540 | -0.61886 | 0.001177 | 0.006673 | RS15565 | 1.512718 | 2.51E-05 | 0.000257 |
| RS14680 | -0.61864 | 0.006464 | 0.026664 | RS08505 | 1.515864 | 2.19E-08 | 7.2E-07 |
| RS28665 | -0.61838 | 0.001126 | 0.006453 | RS00605 | 1.527872 | 2.7E-08 | 8.35E-07 |
| RS28085 | -0.61715 | 0.005942 | 0.025024 | RS12660 | 1.52914 | 0.000234 | 0.001692 |
| RS25285 | -0.61455 | 0.005157 | 0.022411 | RS29885 | 1.530611 | 1.22E-07 | 2.88E-06 |
| RS03900 | -0.61355 | 0.018145 | 0.060384 | RS32015 | 1.534263 | 9.14E-06 | 0.000111 |
| RS25220 | -0.61106 | 0.020688 | 0.067133 | RS27710 | 1.543321 | 0.000117 | 0.000943 |
| RS08880 | -0.60773 | 0.0114 | 0.041782 | RS18155 | 1.552513 | 1.27E-09 | 6.67E-08 |
| RS15920 | -0.60722 | 0.038796 | 0.108481 | RS01045 | 1.556806 | 4.5E-06 | 6.17E-05 |
| RS10435 | -0.60514 | 0.042604 | 0.117256 | RS23330 | 1.562606 | 1.62E-05 | 0.000179 |
| RS20180 | -0.60494 | 0.021491 | 0.068806 | RS14230 | 1.568942 | 6.6E-06 | 8.33E-05 |
| RS04900 | -0.60282 | 0.0002 | 0.001477 | RS14140 | 1.569819 | 1.24E-14 | 3.63E-12 |
| RS07665 | -0.60208 | 0.021065 | 0.06794 | RS07170 | 1.571436 | 1.67E-08 | 5.72E-07 |
| RS26225 | -0.60076 | 0.001991 | 0.010201 | RS31265 | 1.572489 | 1.62E-09 | 7.93E-08 |
| RS23740 | -0.59992 | 0.041086 | 0.114009 | RS26735 | 1.577634 | 0.000272 | 0.001938 |
| RS07505 | -0.59478 | 0.002338 | 0.011678 | RS05015 | 1.585339 | 5.63E-06 | 7.38E-05 |
| RS02295 | -0.59428 | 0.007656 | 0.030264 | RS11540 | 1.590507 | 1.09E-06 | 1.87E-05 |
| RS07690 | -0.59425 | 0.013422 | 0.047597 | RS25945 | 1.598137 | 0.00921 | 0.035197 |
| RS27295 | -0.59134 | 0.023855 | 0.075153 | RS21890 | 1.598373 | 2.72E-09 | 1.22E-07 |
| RS01710 | -0.5896 | 0.010582 | 0.039412 | RS24260 | 1.601657 | 7.62E-11 | 6.29E-09 |
| RS00290 | -0.58813 | 0.024663 | 0.07698 | RS11125 | 1.603534 | 9.86E-08 | 2.46E-06 |
| RS07300 | -0.58715 | 0.002134 | 0.010859 | RS06410 | 1.605431 | 5.94E-14 | 1.44E-11 |
| RS03150 | -0.58642 | 0.000408 | 0.00272 | RS30960 | 1.607198 | 9.41E-05 | 0.000789 |
| RS04140 | -0.58575 | 0.035543 | 0.101262 | RS06450 | 1.611946 | 4.67E-10 | 2.84E-08 |
| RS04105 | -0.58546 | 0.029704 | 0.088547 | RS29845 | 1.613961 | 4.8E-06 | 6.49E-05 |
| RS28345 | -0.5851 | 0.000116 | 0.000937 | RS02595 | 1.616797 | 0.035166 | 0.100579 |
| RS09635 | -0.58348 | 0.001267 | 0.007062 | RS14980 | 1.620949 | 3.01E-09 | 1.31E-07 |
| RS01665 | -0.58194 | 0.017797 | 0.059679 | RS06945 | 1.621756 | 3.68E-10 | 2.27E-08 |
| RS20350 | -0.58163 | 0.026812 | 0.082105 | RS00210 | 1.635202 | 3.17E-09 | 1.34E-07 |
| RS15330 | -0.58126 | 0.029606 | 0.088314 | RS06195 | 1.637411 | 8.22E-06 | 0.000101 |
| RS07245 | -0.58126 | 0.043529 | 0.119349 | RS01765 | 1.642491 | 4.2E-06 | 5.79E-05 |
| RS19380 | -0.5793 | 0.000865 | 0.005194 | RS30080 | 1.646194 | 1.32E-09 | 6.81E-08 |
| RS00860 | -0.57651 | 0.037082 | 0.104624 | RS06415 | 1.649262 | 1.18E-10 | 9.03E-09 |
| RS20170 | -0.57616 | 0.000587 | 0.003693 | RS14925 | 1.654147 | 2.63E-08 | 8.33E-07 |
| RS12690 | -0.57537 | 0.01362 | 0.048103 | RS07560 | 1.655714 | 4.81E-08 | 1.28E-06 |
| RS07880 | -0.57529 | 0.008486 | 0.032775 | RS09290 | 1.658192 | 0.027858 | 0.084658 |
| RS27305 | -0.57258 | 0.001127 | 0.006453 | RS11780 | 1.660462 | 7.35E-05 | 0.000642 |
| RS05430 | -0.57137 | 0.003339 | 0.015654 | RS27635 | 1.661129 | 8.39E-07 | 1.49E-05 |
| RS32280 | -0.57095 | 0.000885 | 0.00527 | RS05930 | 1.666703 | 1.11E-07 | 2.7E-06 |
| RS18650 | -0.57086 | 0.025992 | 0.080156 | RS06170 | 1.670089 | 9.04E-05 | 0.000765 |
| RS16290 | -0.57046 | 0.022825 | 0.072221 | RS06200 | 1.677545 | 8.7E-05 | 0.000742 |
| RS11575 | -0.5698 | 0.005265 | 0.022721 | RS01335 | 1.681148 | 0.029038 | 0.086914 |
| RS13655 | -0.56977 | 0.032537 | 0.094481 | RS06475 | 1.684041 | 3.8E-08 | 1.09E-06 |
| RS07960 | -0.56902 | 5.63E-05 | 0.000517 | RS06935 | 1.685981 | 2.94E-07 | 6.31E-06 |
| RS07255 | -0.56898 | 6.27E-07 | 1.18E-05 | RS19790 | 1.687189 | 9.75E-07 | 1.69E-05 |
| RS06345 | -0.56896 | 0.00018 | 0.001361 | RS24780 | 1.693569 | 0.001291 | 0.007149 |
| RS01655 | -0.56812 | 0.000458 | 0.003002 | RS32040 | 1.701591 | 2.32E-08 | 7.54E-07 |
| RS28315 | -0.5679 | 0.003766 | 0.017335 | RS09990 | 1.703933 | 1.7E-06 | 2.74E-05 |
| RS05395 | -0.56594 | 0.027914 | 0.08471 | RS00940 | 1.711922 | 3.61E-07 | 7.49E-06 |
| RS09605 | -0.56587 | 0.004135 | 0.018753 | RS12585 | 1.732974 | 7.04E-06 | 8.81E-05 |
| RS00490 | -0.56527 | 0.024254 | 0.075994 | RS04765 | 1.757776 | 3.22E-08 | 9.45E-07 |
| RS11630 | -0.56504 | 0.017497 | 0.058963 | RS19965 | 1.764599 | 3.35E-08 | 9.65E-07 |
| RS07620 | -0.56274 | 0.001611 | 0.008608 | RS31275 | 1.766929 | 9.53E-09 | 3.56E-07 |
| RS23030 | -0.56265 | 0.007472 | 0.02978 | RS06175 | 1.775897 | 1.7E-05 | 0.000184 |
| RS32305 | -0.56256 | 0.006885 | 0.027943 | RS28460 | 1.782425 | 6.11E-11 | 5.35E-09 |
| RS25520 | -0.56185 | 0.013404 | 0.047572 | RS06755 | 1.784507 | 5.56E-08 | 1.45E-06 |
| RS23710 | -0.56097 | 0.000831 | 0.005024 | RS03380 | 1.785984 | 1.87E-08 | 6.33E-07 |
| RS02190 | -0.5603 | 0.002397 | 0.011919 | RS30090 | 1.790281 | 7.02E-10 | 4.04E-08 |
| RS25750 | -0.55858 | 0.017631 | 0.059282 | RS09800 | 1.797155 | 6.27E-13 | 9.8E-11 |
| RS20195 | -0.55845 | 0.009676 | 0.036503 | RS15460 | 1.79758 | 0.001022 | 0.005966 |
| RS10590 | -0.55789 | 0.021339 | 0.068512 | RS22870 | 1.808016 | 2.5E-09 | 1.14E-07 |
| RS13000 | -0.55725 | 0.004945 | 0.021617 | RS06190 | 1.809839 | 1.21E-06 | 2.07E-05 |
| RS02300 | -0.55663 | 0.001855 | 0.009653 | RS04230 | 1.83303 | 1.79E-12 | 2.45E-10 |
| RS25175 | -0.55651 | 0.014996 | 0.05208 | RS04775 | 1.844653 | 9.39E-15 | 2.93E-12 |
| RS04425 | -0.55589 | 0.014156 | 0.049718 | RS04330 | 1.858425 | 9.68E-08 | 2.43E-06 |
| RS25170 | -0.55369 | 0.000308 | 0.002144 | RS09700 | 1.860087 | 0.028555 | 0.085964 |
| RS10295 | -0.55351 | 0.010816 | 0.040076 | RS32025 | 1.872879 | 1.49E-06 | 2.48E-05 |
| RS32005 | -0.55205 | 0.012523 | 0.045029 | RS25630 | 1.887461 | 1.79E-05 | 0.000193 |
| RS03070 | -0.55147 | 6.32E-05 | 0.000568 | RS05750 | 1.896522 | 0.011052 | 0.040826 |
| RS31950 | -0.55121 | 0.044433 | 0.121167 | RS00180 | 1.897939 | 3.68E-05 | 0.000358 |
| RS01670 | -0.55116 | 0.008009 | 0.031321 | RS04205 | 1.903751 | 4.25E-05 | 0.000408 |
| RS04440 | -0.55015 | 0.020726 | 0.067184 | RS18925 | 1.90501 | 1.27E-06 | 2.15E-05 |
| RS25000 | -0.54921 | 0.006465 | 0.026664 | RS20200 | 1.909092 | 3.92E-11 | 3.57E-09 |
| RS25005 | -0.54839 | 0.008232 | 0.032021 | RS28455 | 1.922539 | 1.57E-13 | 3.52E-11 |
| RS12045 | -0.54833 | 0.048962 | 0.130883 | RS06180 | 1.926122 | 5.73E-07 | 1.1E-05 |
| RS32340 | -0.54738 | 0.015139 | 0.052452 | RS16795 | 1.929087 | 7.94E-07 | 1.43E-05 |
| RS29080 | -0.54384 | 0.000489 | 0.003172 | RS19950 | 1.929989 | 0.001488 | 0.008041 |
| RS13005 | -0.54382 | 0.036789 | 0.10393 | RS06405 | 1.941197 | 8.76E-09 | 3.3E-07 |
| RS20885 | -0.54192 | 0.002298 | 0.011493 | RS01850 | 1.95978 | 0.000166 | 0.001271 |
| RS23140 | -0.54096 | 0.049374 | 0.131664 | RS23005 | 1.966058 | 9.88E-09 | 3.66E-07 |
| RS26630 | -0.54004 | 0.0154 | 0.053148 | RS06425 | 1.970095 | 1.88E-11 | 1.87E-09 |
| RS32295 | -0.54004 | 0.001432 | 0.007815 | RS03370 | 1.976103 | 2.8E-07 | 6.04E-06 |
| RS31610 | -0.53477 | 0.031797 | 0.092757 | RS07650 | 1.976651 | 3.58E-07 | 7.45E-06 |
| RS16160 | -0.53355 | 0.006132 | 0.025628 | RS18415 | 1.98945 | 3.95E-08 | 1.11E-06 |
| RS20980 | -0.5333 | 0.003983 | 0.018197 | RS15450 | 1.995552 | 2.82E-08 | 8.58E-07 |
| RS04950 | -0.53328 | 0.013274 | 0.047149 | RS06215 | 1.999395 | 6.86E-06 | 8.63E-05 |
| RS20725 | -0.53242 | 0.020686 | 0.067133 | RS13380 | 2.000712 | 2.31E-12 | 2.81E-10 |
| RS22855 | -0.52971 | 0.003289 | 0.015459 | RS25640 | 2.023805 | 0.022809 | 0.072221 |
| RS21880 | -0.52582 | 0.012954 | 0.046237 | RS29350 | 2.02864 | 1.75E-25 | 3.82E-22 |
| RS08100 | -0.52436 | 0.001045 | 0.006067 | RS07645 | 2.034391 | 1.08E-08 | 3.97E-07 |
| RS15475 | -0.5232 | 0.005833 | 0.024639 | RS19850 | 2.034852 | 2.17E-10 | 1.46E-08 |
| RS07335 | -0.52247 | 0.006044 | 0.025335 | RS11695 | 2.038534 | 1.18E-07 | 2.81E-06 |
| RS10035 | -0.52148 | 0.022581 | 0.071709 | RS29795 | 2.04039 | 6.15E-13 | 9.8E-11 |
| RS24150 | -0.5193 | 0.03171 | 0.092658 | RS03065 | 2.042284 | 3.43E-13 | 6.02E-11 |
| RS06700 | -0.51822 | 5.96E-05 | 0.000541 | RS04525 | 2.066597 | 1.6E-06 | 2.61E-05 |
| RS12795 | -0.51663 | 0.048805 | 0.130545 | RS11140 | 2.067243 | 6.06E-07 | 1.15E-05 |
| RS07240 | -0.5161 | 0.021452 | 0.068773 | RS15515 | 2.072088 | 3.67E-07 | 7.58E-06 |
| RS09625 | -0.51533 | 7.93E-06 | 9.8E-05 | RS04380 | 2.081723 | 2.26E-09 | 1.05E-07 |
| RS12670 | -0.5152 | 0.005254 | 0.022697 | RS11665 | 2.082359 | 1.63E-09 | 7.93E-08 |
| RS22395 | -0.5151 | 0.028451 | 0.085777 | RS04075 | 2.092097 | 0.000151 | 0.00117 |
| RS07790 | -0.51466 | 0.028648 | 0.086028 | RS23770 | 2.093123 | 1.39E-10 | 1.03E-08 |
| RS08150 | -0.51455 | 0.008293 | 0.032201 | RS27690 | 2.102625 | 7.26E-09 | 2.84E-07 |
| RS03175 | -0.51447 | 0.004768 | 0.020992 | RS19265 | 2.107257 | 3.41E-10 | 2.13E-08 |
| RS15910 | -0.51443 | 0.000479 | 0.003108 | RS04095 | 2.112158 | 1.03E-05 | 0.000121 |
| RS16340 | -0.51342 | 0.022368 | 0.071085 | RS06210 | 2.178439 | 1.53E-06 | 2.53E-05 |
| RS27465 | -0.51287 | 0.025471 | 0.079125 | RS25460 | 2.213511 | 2.73E-08 | 8.35E-07 |
| RS07815 | -0.51253 | 2.92E-06 | 4.3E-05 | RS01845 | 2.247354 | 7.12E-15 | 2.4E-12 |
| RS21515 | -0.5121 | 0.027715 | 0.08434 | RS07540 | 2.260433 | 2.07E-12 | 2.67E-10 |
| RS05035 | -0.51202 | 0.007638 | 0.030222 | RS06420 | 2.269473 | 2.78E-10 | 1.76E-08 |
| RS16105 | -0.50991 | 0.037142 | 0.104724 | RS03060 | 2.284422 | 1.14E-10 | 8.91E-09 |
| RS13685 | -0.50757 | 0.025749 | 0.079773 | RS22205 | 2.299878 | 2.94E-15 | 1.17E-12 |
| RS25550 | -0.50647 | 0.000551 | 0.003507 | RS16805 | 2.318684 | 5.44E-13 | 9.15E-11 |
| RS16125 | -0.506 | 0.008885 | 0.034061 | RS22210 | 2.346489 | 2.92E-09 | 1.28E-07 |
| RS28270 | -0.50577 | 0.009502 | 0.036095 | RS23715 | 2.382532 | 1.61E-13 | 3.52E-11 |
| RS21415 | -0.50541 | 0.001846 | 0.009628 | RS09730 | 2.387531 | 6.28E-10 | 3.66E-08 |
| RS08410 | -0.50495 | 0.049676 | 0.132308 | RS01360 | 2.43221 | 0.02607 | NA |
| RS19720 | -0.5024 | 0.03899 | 0.108885 | RS03375 | 2.442737 | 2.45E-14 | 6.69E-12 |
| RS29010 | 0.500673 | 0.03025 | 0.089805 | RS11655 | 2.445592 | 8.85E-12 | 9.67E-10 |
| RS31195 | 0.501034 | 0.001209 | 0.006812 | RS06220 | 2.490375 | 1.31E-08 | 4.6E-07 |
| RS07895 | 0.501302 | 0.040612 | 0.112836 | RS12415 | 2.4913 | 1.27E-12 | 1.85E-10 |
| RS20160 | 0.501734 | 0.000337 | 0.002318 | RS11700 | 2.534146 | 2.69E-19 | 2.36E-16 |
| RS26740 | 0.502209 | 0.026721 | 0.081886 | RS00935 | 2.61063 | 9.06E-05 | 0.000765 |
| RS31375 | 0.505297 | 0.002467 | 0.012214 | RS16755 | 2.617153 | 0.000193 | 0.001446 |
| RS08530 | 0.509164 | 0.00106 | 0.006145 | RS06005 | 2.694946 | 1.86E-18 | 1.25E-15 |
| RS19835 | 0.510851 | 0.04122 | 0.114236 | RS01855 | 2.712294 | 4.04E-09 | 1.67E-07 |
| RS23485 | 0.512082 | 0.025477 | 0.079125 | RS31735 | 2.782137 | 0.031609 | NA |
| RS03020 | 0.512125 | 0.021693 | 0.069392 | RS06590 | 2.941013 | 1.96E-05 | 0.000209 |
| RS07965 | 0.512615 | 0.035212 | 0.100646 | RS22400 | 3.064882 | 7.82E-09 | 3E-07 |
| RS07565 | 0.513872 | 0.027389 | 0.083521 | RS19280 | 3.707372 | 4.88E-56 | 2.14E-52 |
| RS29355 | 0.514189 | 0.011774 | 0.042757 | RS28310 | 3.740694 | 3.28E-08 | 9.58E-07 |
| RS28195 | 0.51527 | 0.002107 | 0.010732 | RS26895 | 3.918192 | 1.96E-05 | 0.000209 |
| RS02475 | 0.515598 | 0.004759 | 0.020974 | RS11905 | 4.391446 | 0.024751 | NA |
| RS12370 | 0.519347 | 0.034978 | 0.100239 | RS24010 | 4.593673 | 0.011917 | NA |
| RS10465 | 0.519596 | 0.006044 | 0.025335 |  |  |  |  |

**Table S2. Strains and plasmids used in this study.**

| **Strains and plasmids** | **Characteristics** | **Source** |
| --- | --- | --- |
| ***Psa*** |  |  |
| WT | *Pseudomonas syringae* pv. *actinidiae* M228 Wild Type | (Wang et al. 2021) |
| Δ*TrpR2* | In-frame deletion of *TrpR2*(RS16350) in strain M228 Wild Type | This study |
| ΔRS02395 | In-frame deletion of RS02395 in strain M228 Wild Type | This study |
| ΔRS07360 | In-frame deletion of RS07360 in strain M228 Wild Type | This study |
| ΔRS29160 | In-frame deletion of RS29160 in strain M228 Wild Type | This study |
| ΔRS25575 | In-frame deletion of RS25575 in strain M228 Wild Type | This study |
| ΔRS27290 | In-frame deletion of RS27290 in strain M228 Wild Type | This study |
| ΔRS27605 | In-frame deletion of RS27605 in strain M228 Wild Type | This study |
| ΔRS01550 | In-frame deletion of RS01550 in strain M228 Wild Type | This study |
| ΔRS05155 | In-frame deletion of RS05155 in strain M228 Wild Type | This study |
| ΔRS18405 | In-frame deletion of RS18405 in strain M228 Wild Type | This study |
| ΔRS21050 | In-frame deletion of RS21050 in strain M228 Wild Type | This study |
| ΔRS24920 | In-frame deletion of RS24920 in strain M228 Wild Type | This study |
| ΔRS25395 | In-frame deletion of RS25395 in strain M228 Wild Type | This study |
| ΔRS25535 | In-frame deletion of RS25535 in strain M228 Wild Type | This study |
| ΔRS25570 | In-frame deletion of RS25570 in strain M228 Wild Type | This study |
| ***E. coli*** |  |  |
| DH5α | *F –φ80lacZ ΔM15 Δ(lacZYA-argF)U169 recA1 endA1 hsdR17(rk– , mk+)phoA supE44 thi-1 gyrA96 relA1 tonA* | Stratagene |
| BL21 | *F-, ompT, hsdSB (rBB- mB- ), gal, dcm ( DE3 )* | Stratagene |
| XL1-Blue MRF^’^ Kan | Host for bacterial two-hybrid assay | (Xu et al. 2018) |
| Top10 | *E. coli* Top10 harbouring plasmid pPROBE-*gfp*, Gm^R^ | Laboratory stock |
| B2H (+) | The positive control strain of bacterial two-hybrid assey, Km^R^, Str^R^, TC^R^, Chlo^R^ | (Xu et al. 2018) |
| B2H (-) | The negative control strain of bacterial two-hybrid assey, Km^R^, Str^R^, TC^R^, Chlo^R^ | (Xu et al. 2018) |
| **Plasmids** |  |  |
| pMS402 | Reporter plasmid carring the promoterless *lux* CDABE,Km^R^ | (Duan et al. 2003) |
| pK18mobsacB | Suicide vector with a sacB gene, Km^R^ | (Shao et al. 2021) |
| pHM1 | Broad-host-range *cos* IncW derivative of Pri40, Spe^R^ | (Innes, Hirose, and Kuempel 1988) |
| pBT | The bait vector used for protein expression in bacterial two-hybridization assay, Chlo^R^ | (Liao et al. 2021) |
| pTRG | The target vector used for protein expression in bacterial two-hybridization assay, Tc^R^ | (Liao et al. 2021) |
| pGEX-6p-1 | Protein expression vector with a GST tag, AmpR | Laboratory stock |
| pET28a | Protein expression vector with a His tag, Km^R^ | Laboratory stock |
| pVS10 | *E. coli rpoA*-*rpoB*-*rpoC*[His6] and *rpoZ* genes under control of the T7 promoter. Amp^R^ | (Belogurov et al. 2007) |
| pK18-*TrpR2* | Suicide vector for *TrpR2* deletion mutant in M228, Km^R^ | This study |
| pKD-*hopY1* (M228)*-lux* | Transcriptional fusion between *hopY1*(M228) promoter and *lux* in pMS402, Km^R^ | This study |
| pKD-*hopAK1* (M228)*-lux* | Transcriptional fusion between *hopAK1*(M228) promoter and *lux* in pMS402, Km^R^ | This study |
| pKD-*hrpK1* (M228)*-lux* | Transcriptional fusion between *hrpK1*(M228) promoter and *lux* in pMS402, Km^R^ | This study |
| pKD-*hrpZ1* (M228)*-lux* | Transcriptional fusion between *hrpZ1*(M228) promoter and *lux* in pMS402, Km^R^ | This study |
| pPROBE-GFP | The vector used for GFP expression, Km^R^ | Laboratory stock |
| pHM1-TrpR2 | Overexpression TrpR2 in M228, Spe^R^ | This study |
| pHM1-TrpR2_D18A_ | Overexpression TrpR2 but the 18th aspartic acid mutated to alanine in M228, Spe^R^ | This study |
| pHM1-TrpR2_P37A_ | Overexpression TrpR2 but the 37th proline acid mutated to alanine in M228, Spe^R^ | This study |
| pHM1-TrpR2_G57A_ | Overexpression TrpR2 but the 57th glycine acid mutated to alanine in M228, Spe^R^ | This study |
| pHM1-TrpR2_L72A_ | Overexpression TrpR2 but the 72nd leucine acid mutated to alanine in M228, Spe^R^ | This study |
| pHM1-TrpR2_G76A_ | Overexpression TrpR2 but the 76th glycine acid mutated to alanine in M228, Spe^R^ | This study |
| pHM1-TrpR2_F98A_ | Overexpression TrpR2 but the 98th phenylalanine acid mutated to alanine in M228, Spe^R^ | This study |
| pHM1-TrpR2_L104A_ | Overexpression TrpR2 but the 104th leucine acid mutated to alanine in M228, Spe^R^ | This study |
| pHM1-TrpR2_G118A_ | Overexpression TrpR2 but the 118th glycine acid mutated to alanine in M228, Spe^R^ | This study |
| pHM1-TrpR2_L120A_ | Overexpression TrpR2 but the 120th leucine acid mutated to alanine in M228, Spe^R^ | This study |
| pHM1-TrpR2_S14A_ | Overexpression TrpR2 but the 14th serine acid mutated to alanine in M228, Spe^R^ | This study |
| pHM1-TrpR2_R96A_ | Overexpression TrpR2 but the 96th arginine acid mutated to alanine in M228, Spe^R^ | This study |
| pBT-TrpR2 | pBT carrying TrpR2 in M228, Chlo^R^ | This study |
| pTRG-HrpR | pTRG carrying HrpR in M228, Tc^R^ | This study |
| pTRG-HrpS | pTRG carrying HrpS in M228, Tc^R^ | This study |
| pTRG-HrpL | pTRG carrying HrpL in M228, Tc^R^ | This study |
| pBT-TrpR2_D18A_ | pBT carrying TrpR2_D18A_ in M228, Chlo^R^ | This study |
| pBT-TrpR2_P37A_ | pBT carrying TrpR2_P37A_ in M228, Chlo^R^ | This study |
| pBT-TrpR2_G57A_ | pBT carrying TrpR2_G57A_ in M228, Chlo^R^ | This study |
| pBT-TrpR2_L72A_ | pBT carrying TrpR2_L72A_ in M228, Chlo^R^ | This study |
| pBT-TrpR2_G76A_ | pBT carrying TrpR2_G76A_ in M228, Chlo^R^ | This study |
| pBT-TrpR2_F98A_ | pBT carrying TrpR2_F98A_ in M228, Chlo^R^ | This study |
| pBT-TrpR2_L104A_ | pBT carrying TrpR2_L104A_ in M228, Chlo^R^ | This study |
| pBT-TrpR2_G118A_ | pBT carrying TrpR2_G118A_ in M228, Chlo^R^ | This study |
| pBT-TrpR2_L120A_ | pBT carrying TrpR2_L120A_ in M228, Chlo^R^ | This study |
| pBT-TrpR2_R12A-S14A_ | pBT carrying TrpR2_R12A-S14A_ in M228, Chlo^R^ | This study |
| pBT-TrpR2_N30A -P37A_ | pBT carrying TrpR2_N30A-P37A_ in M228, Chlo^R^ | This study |
| pBT-TrpR2_R96A_ | pBT carrying TrpR2_R96A_ in M228, Chlo^R^ | This study |
| pBT-TrpR2_R12A_ | pBT carrying TrpR2_R12A_ in M228, Chlo^R^ | This study |
| pBT-TrpR2_H13A_ | pBT carrying TrpR2_H13A_ in M228, Chlo^R^ | This study |
| pBT-TrpR2_S14A_ | pBT carrying TrpR2_S14A_ in M228, Chlo^R^ | This study |
| pTRG-HrpL_N43A-D48A_ | pTRG carrying HrpL_N43A to D48A_ in M228, Tc^R^ | This study |
| pTRG-HrpL_H86A_ | pTRG carrying HrpL_H86A_ in M228, Tc^R^ | This study |
| pTRG-HrpL_L89A-M90A_ | pTRG carrying HrpL_L89A to M90A_ in M228, Tc^R^ | This study |
| pTRG-HrpL_S144A_ | pTRG carrying HrpL_S144A_ in M228, Tc^R^ | This study |
| pTRG-HrpL_D148A-Y151A_ | pTRG carrying HrpL_D148A to Y151A_ in M228, Tc^R^ | This study |
| pTRG-HrpL_D148A_ | pTRG carrying HrpL_D148A_ in M228, Tc^R^ | This study |
| pTRG-HrpL_G149A_ | pTRG carrying HrpL_G149A_ in M228, Tc^R^ | This study |
| pTRG-HrpL_N150A_ | pTRG carrying HrpL_N150A_ in M228, Tc^R^ | This study |
| pTRG-HrpL_Y151A_ | pTRG carrying HrpL_Y151A_ in M228, Tc^R^ | This study |
| pETDuet | pETDuet-1 is used to express two genes of interest simultaneously, Amp^R^ | (Addinall et al. 2005) |
| pETDuet-HrpL | Express HrpL in BL21, Amp^R^ | This study |
| pETDuet-HrpL+TrpR2 | Express HrpL and TrpR2 simultaneously in BL21, Amp^R^ | This study |

**Table S3. Primers used in this study.**

| Primer | Sequence | Purpose |
| --- | --- | --- |
| M13 (-47) F | CGCCAGGGTTTTCCCAGTCACGAC | Universal primer for pHM1 |
| M13 (-48) R | AGCGGATAACAATTTCACACAGGA |  |
| pKD-*hopY1*-*lux*-F (*BamH*I) | TCGTCTTCACCTCGAGGGGATCCCTCTGGCGGATCTGGAGC | To detect the expression level of *hopY1* by luciferase(*lux*)-based reporter assay |
| pKD-*hopY1*-*lux*-R (*BamH*I) | GCGGCCGCAACTAGAGGATCCCGCGTGGAGTTGGGAATG |  |
| pKD-*hrpL*-*lux*-F (*BamH*I) | TCGTCTTCACCTCGAGGGGATCCAGCTGGCCGATGTTTTTG | To detect the expression level of *hrpL* by luciferase(*lux*)-based reporter assay |
| pKD-*hrpL*-*lux*-R (*BamH*I) | GCGGCCGCAACTAGAGGATCCACATGGGCTTACCCTGAT |  |
| pKD-*hopAK1*-*lux*-F (*BamH*I) | TCGTCTTCACCTCGAGGGGATCCGATTTTGGCCCCGCGTAA | To detect the expression level of *hopAK1* by luciferase(*lux*)-based reporter assay |
| pKD-*hopAK1*-*lux*-R (*BamH*I) | GCGGCCGCAACTAGAGGATCCCGATGAGTGCGGACCATTG |  |
| pKD-*hrpK1*-*lux*-F (*BamH*I) | TCGTCTTCACCTCGAGGGGATCCGGAAGTATTCGCATGATA | To detect the expression level of *hrpK1* by luciferase(*lux*)-based reporter assay |
| pKD-*hrpK1*-*lux*-R (*BamH*I) | GCGGCCGCAACTAGAGGATCCGGGCTTACCCTGATTTAA |  |
| pKD-*hrpZ1*-*lux*-F (*BamH*I) | TCGTCTTCACCTCGAGGGGATCCCAATTGCGAGTGATCGAA | To detect the expression level of *hrpZ1* by luciferase(*lux*)-based reporter assay |
| pKD-*hrpZ1*-*lux*-R (*BamH*I) | GCGGCCGCAACTAGAGGATCCGATTTGATGCCCCTTAAG |  |
| pHM1-TrpR2(M228)-F (*Hind*III) | ATGACCATGATTACGCCAAGCTTTGACAAACAGGAGAAATT | To construct a vector that Overexpress TrpR2 in M228 |
| pHM1-TrpR2(M228)-R (*Hind*III) | GACCTGCAGGCATGCAAGCTTTTGCTTACCGCGTATTGA |  |
| pBT-TrpR2(M228)-F (*BamH*I) | GGCGCGGCCGCATCGAATTCCTGACAAACAGGAGAAATT | To construct pBT carrying TrpR2 in M228 |
| pBT-TrpR2(M228)-R (*EcoR*I) | AATTAATTAACTCGAGGATCCACCGCGTATTGATCAGCT |  |
| pRTG-HrpS(M228)-F (*BamH*I) | AAACCAGAGGCGGCCGGATCCATGAGTCTTGATGAAAGG | To construct pTRG carrying HrpS in M228 |
| pRTG-HrpS(M228)-R (*EcoR*I) | GCGCCAGCTCAGACTGAATTCCAGCGTCTTTGCAAAAAA |  |
| pRTG-HrpL(M228)-F (*BamH*I) | AAACCAGAGGCGGCCGGATCCCCATTAAATCAGGGTAAG | To construct pTRG carrying HrpL in M228 |
| pRTG-HrpL(M228)-R (*EcoR*I) | GCGCCAGCTCAGACTGAATTCAAAAAATGATCGAGATCG |  |
| pRTG-HrpR(M228)-F (*BamH*I) | AAACCAGAGGCGGCCGGATCCCACCCACAATGAGAGTGA | To construct pTRG carrying HrpR in M228 |
| pRTG-HrpR(M228)-R (*EcoR*I) | GCGCCAGCTCAGACTGAATTCCATAACCACACCTGCGTT |  |
| pK18-16350(M228)-up-F (*EcoR*I) | ctatgacatgattacgaattcGCCGTATACGTCTCCTGGCTG | To amplify a 1500-bp fragment of the upstream of 16350 |
| pK18-16350(M228)-up-R (*BamH*I) | gcttaccgcgtattgaCATGATAATTTCTCCTGTTTGTCAGC |  |
| pK18-16350(M228)-down-F (*EcoR*I) | catgTCAATACGCGGTAAGCAACACT | To amplify a 1500-bp fragment of the downstream of 16350 |
| pK18-16350(M228)-down-R(*BamH*I) | caggtcgactctagaggatccTGCTGGATGTATCGGTCGAAT |  |
| pK18-02395(M228)-up-F (*EcoR*I) | ctatgacatgattacgaattcAAGACCTGACGCAAGGCTTTT | To amplify a 1500-bp fragment of the upstream of 02395 |
| pK18-02395(M228)-up-R (*BamH*I) | cagtcatAGAGCATTCAGCGCGTCTCC |  |
| pK18-02395(M228)-down-F (*EcoR*I) | cgctgaatgctctATGACTGACTGCGCGCCG | To amplify a 1500-bp fragment of the downstream of 02395 |
| pK18-02395(M228)-down-R (*BamH*I) | caggtcgactctagaggatccCAGGCGTAAACCCGTCGG |  |
| pK18-07360(M228)-up-F(*EcoR*I) | ctatgacatgattacgaattcGGGGTTTGCCTGTTTTTACGA | To amplify a 1500-bp fragment of the upstream of 07360 |
| pK18-07360(M228)-up-R (*BamH*I) | aacaatccgaGGATCAGGTCTTAATAACACAGTCATAA |  |
| pK18-07360(M228)-down-F (*EcoR*I) | gacctgatccTCGGATTGTTCTACTGACAATCTCC | To amplify a 1500-bp fragment of the downstream of 07360 |
| pK18-07360(M228)-down-R (*BamH*I) | caggtcgactctagaggatccGCGACGAAATCGTTTTTTCTACT |  |
| pK18-29160(M228)-up-F(*EcoR*I) | ctatgacatgattacgaattcAGTTGGCAGTTGCGAAGAAAA | To amplify a 1500-bp fragment of the upstream of 29160 |
| pK18-29160(M228)-up-R (*BamH*I) | cagccagcatGGTTATTCACGGCCCTCTGG |  |
| pK18-29160(M228)-down-F (*EcoR*I) | gtgaataaccATGCTGGCTGCAAGCGGT | To amplify a 1500-bp fragment of the downstream of 29160 |
| pK18-29160(M228)-down-R (*BamH*I) | caggtcgactctagaggatccAGGCTTTTTTCCGGGCTCA |  |
| pK18-27610(M228)-up-F (*EcoR*I) | ctatgacatgattacgaattcAAACCGATCGCAGAAGACGC | To amplify a 1500-bp fragment of the upstream of 27610 |
| pK18-27610(M228)-up-R (*BamH*I) | gccGTCTTTTACGTTCCAGTCAAAAAACG |  |
| pK18-27610(M228)-down-F (*EcoR*I) | actggaacgtaaaagacGGCGGCAGCGGACAAAGG | To amplify a 1500-bp fragment of the downstream of 27610 |
| pK18-27610(M228)-down-R (*BamH*I) | caggtcgactctagaggatccACGCCAGCGCCAAGACGC |  |
| pK18-25575(M228)-up-F(*EcoR*I) | ctatgacatgattacgaattcGCTGTTCGTCGCCGGAGT | To amplify a 1500-bp fragment of the upstream of 25575 |
| pK18-25575(M228)-up-R (*BamH*I) | cCTTGACTCTCCCGAAACTTGCA |  |
| pK18-25575(M228)-down-F (*EcoR*I) | aagtttcgggagagtcaagGCGCAGGCTCACTTGCAA | To amplify a 1500-bp fragment of the downstream of 25575 |
| pK18-25575(M228)-down-R(*BamH*I) | caggtcgactctagaggatccTACGTCGCATGCCCTGCT |  |
| pK18-27290(M228)-up-F(*EcoR*I) | ctatgacatgattacgaattcTGCCTTGCTGCACCACGT | To amplify a 1500-bp fragment of the upstream of 27290 |
| pK18-27290(M228)-up-R (*BamH*I) | cacgatctTAGTCACCTTCCTTTTAATGCAGG |  |
| pK18-27290(M228)-down-F (*EcoR*I) | ggaaggtgactaAGATCGTGTTTGGCTGATCGG | To amplify a 1500-bp fragment of the downstream of 27290 |
| pK18-27290(M228)-down-R (*BamH*I) | caggtcgactctagaggatccAACCTGACTCAGAACTCAACCAGAA |  |
| pK18-27605(M228)-up-F(*EcoR*I) | ctatgacatgattacgaattcGCATGGCCGAGACGCTGT | To amplify a 1500-bp fragment of the upstream of 27605 |
| pK18-27605(M228)-up-R (*BamH*I) | aactgcctgcGTAACGATCACCCATCCGAATT |  |
| pK18-27605(M228)-down-F (*EcoR*I) | tgatcgttacGCAGGCAGTTGCTCAGCCT | To amplify a 1500-bp fragment of the downstream of 27605 |
| pK18-27605(M228)-down-R (*BamH*I) | caggtcgactctagaggatccCCAGCATACCGAGGTCCTGA |  |
| pK18-01550(M228)-up-F (*EcoR*I) | ctatgacatgattacgaattcATGATCGTGTGGATCTCGTCG | To amplify a 1500-bp fragment of the upstream of 01550 |
| pK18-01550(M228)-up-R (*BamH*I) | gtgacagattCGCCATATCCCTCTGCAAAG |  |
| pK18-01550(M228)-down-F (*EcoR*I) | ggatatggcgAATCTGTCACTCACAAATAGTATGACAGTC | To amplify a 1500-bp fragment of the downstream of 01550 |
| pK18-01550(M228)-down-R (*BamH*I) | caggtcgactctagaggatccGTGTCAGCGTGCTTGGTCAA |  |
| pK18-05155(M228)-up-F (*EcoR*I) | ctatgacatgattacgaattcCAGCACTTCCTCGTCAAGGATG | To amplify a 1500-bp fragment of the upstream of 05155 |
| pK18-05155(M228)-up-R (*BamH*I) | ataggcggtttatcAGGCGTCCCCGGAAACTG |  |
| pK18-05155(M228)-down-F (*EcoR*I) | acgcctGATAAACCGCCTATGGTGGTGT | To amplify a 1500-bp fragment of the downstream of 05155 |
| pK18-05155(M228)-down-R (*BamH*I) | caggtcgactctagaggatccGAAAACTTCTGCAGAGGAGGAGC |  |
| pK18-18405(M228)-up-F (*EcoR*I) | ctatgacatgattacgaattcAGGCAACACCTCCTCCAATCG | To amplify a 1500-bp fragment of the upstream of 18405 |
| pK18-18405(M228)-up-R (*BamH*I) | cttgtcaatcggctaCTCATAATCATCAAGCTCCGATCTG |  |
| pK18-18405(M228)-down-F (*EcoR*I) | atgagTAGCCGATTGACAAGAAACCCG | To amplify a 1500-bp fragment of the downstream of 18405 |
| pK18-18405(M228)-down-R (*BamH*I) | caggtcgactctagaggatccGCGCCAAGCGGTGAAGGT |  |
| pK18-21050(M228)-up-F (*EcoR*I) | ctatgacatgattacgaattcCCCAGAGGACTATCAGAACCTCG | To amplify a 1500-bp fragment of the upstream of 21050 |
| pK18-21050(M228)-up-R (*BamH*I) | CGTTGCGGGTGTCAGTCATT |  |
| pK18-21050(M228)-down-F (*EcoR*I) | aatgactgacacccgcaacgGCCCGCCAAGGTTGCTCC | To amplify a 1500-bp fragment of the downstream of 21050 |
| pK18-21050(M228)-down-R (*BamH*I) | caggtcgactctagaggatccGCCACGCATGTCGCTGCC |  |
| pK18-24920(M228)-up-F (*EcoR*I) | ctatgacatgattacgaattcGCACCCTTGCCGGCCAGG | To amplify a 1500-bp fragment of the upstream of 24920 |
| pK18-24920(M228)-up-R (*BamH*I) | CGGCCAGCATCGCCGAAC |  |
| pK18-24920(M228)-down-F (*EcoR*I) | aagttcggcgatgctggccgGACATGGGCGGCATGGGT | To amplify a 1500-bp fragment of the downstream of 24920 |
| pK18-24920(M228)-down-R (*BamH*I) | caggtcgactctagaggatccGATCGTGCCACGGCTGGC |  |
| pK18-25395(M228)-up-F (*EcoR*I) | ctatgacatgattacgaattcTCACAGCGGTAGCCGAAGC | To amplify a 1500-bp fragment of the upstream of 25395 |
| pK18-25395(M228)-up-R (*BamH*I) | gcgaattactGGGATAAGGGACCCTAGGGAA |  |
| pK18-25395(M228)-down-F (*EcoR*I) | cccttatcccAGTAATTCGCCGGGGTGC | To amplify a 1500-bp fragment of the downstream of 25395 |
| pK18-25395(M228)-down-R (*BamH*I) | caggtcgactctagaggatccCATCAGCCCCAATATCCACG |  |
| pK18-25535(M228)-up-F (*EcoR*I) | ctatgacatgattacgaattcAAATCGTGGGTGAGGACGAGG | To amplify a 1500-bp fragment of the upstream of 25535 |
| pK18-25535(M228)-up-R (*BamH*I) | gGCCGCGATGATCAACCACA |  |
| pK18-25535(M228)-down-F (*EcoR*I) | tgtggttgatcatcgcggcCGGCGGTCCTGCTGCTGA | To amplify a 1500-bp fragment of the downstream of 25535 |
| pK18-25535(M228)-down-R (*BamH*I) | caggtcgactctagaggatccGGGCTGCGCCGAGAGCAC |  |
| pK18-25570(M228)-up-F (*EcoR*I) | ctatgacatgattacgaattcGCATGAGCCTCAAAGGCGA | To amplify a 1500-bp fragment of the upstream of 25570 |
| pK18-25570(M228)-up-R (*BamH*I) | aagccAATTCTTCCGCACTCTTGAGGA |  |
| pK18-25570(M228)-down-F (*EcoR*I) | gagtgcggaagaattGGCTTATCAACGGGAGCAGG | To amplify a 1500-bp fragment of the downstream of 25570 |
| pK18-25570(M228)-down-R (*BamH*I) | caggtcgactctagaggatccAGGCCAGGGAACCCGCGA |  |
| 16350 (M228)-verify-F | CCGAAGCGACCTTGATGTTC | To verify the mutant Δ16350 is successfully constructed or not |
| 16350 (M228)-verify-R | TCATCCGTGCCATCGTCG |  |
| 02395 (M228)-verify-F | CATCTGGTGCTGATCGACAA | To verify the mutant Δ02395 is successfully constructed or not |
| 02395 (M228)-verify-R | TCGGGCTGATCAACATCGTA |  |
| 07360 (M228)-verify-F | CGACCCGTTAGCTTGATTCG | To verify the mutant Δ07360 is successfully constructed or not |
| 07360 (M228)-verify-R | GTCAGCGGAATCATAAGGCG |  |
| 29160 (M228)-verify-F | GACGCGTCCTTGATCATCAC | To verify the mutant Δ29160 is successfully constructed or not |
| 29160 (M228)-verify-R | GGGAGCATTAAGAGGAGCCT |  |
| 27610 (M228)-verify-F | TGTCACGAGGCATCTGGATT | To verify the mutant Δ27610 is successfully constructed or not |
| 27610 (M228)-verify-R | CGTATCTGAAGAGCGAGCTG |  |
| 25575 (M228)-verify-F | CTTAATGGTCGTCTGCTGCG | To verify the mutant Δ25575 is successfully constructed or not |
| 25575 (M228)-verify-R | GTTCAACAGCCAGACGGATC |  |
| 27290 (M228)-verify-F | ACATTTATAAGCTGGCCCGC | To verify the mutant Δ27290 is successfully constructed or not |
| 27290 (M228)-verify-R | CCTCATGAAAAGGGCCCTCA |  |
| 27605 (M228)-verify-F | GCTGCCAACACTTGAACTGA | To verify the mutant Δ27605 is successfully constructed or not |
| 27605 (M228)-verify-R | GAAGAGAGCGACACCCAGAG |  |
| 01550 (M228)-verify-F | AGGTGTCATTGCGTCTGG | To verify the mutant Δ01550 is successfully constructed or not |
| 01550 (M228)-verify-R | GTCGGGGATTCTCTCGGA |  |
| 05155 (M228)-verify-F | CGAAATGCATGCCAACGC | To verify the mutant Δ05155 is successfully constructed or not |
| 05155 (M228)-verify-R | CGGAGAGATGCGGGAGAG |  |
| 18405 (M228)-verify-F | TGTCGCGCCGGTTTCAAG | To verify the mutant Δ18405 is successfully constructed or not |
| 18405 (M228)-verify-R | CGGCATCCTGACTGACACC |  |
| 21050 (M228)-verify-F | ACCATCGAGCGTGATACCG | To verify the mutant Δ21050 is successfully constructed or not |
| 21050 (M228)-verify-R | CTCTACGGCGGCCAGTTG |  |
| 24920 (M228)-verify-F | CAAGGTTGTGTTCGGTCCT | To verify the mutant Δ24920 is successfully constructed or not |
| 24920 (M228)-verify-R | GCCATCACATACGCCGAG |  |
| 25395 (M228)-verify-F | TCGCGTTATTACTGTCTCAAGC | To verify the mutant Δ25395 is successfully constructed or not |
| 25395 (M228)-verify-R | AGACGTACACCCAGCGAC |  |
| 25535 (M228)-verify-F | GTGCAGCATCAACGAACATTACG | To verify the mutant Δ25535 is successfully constructed or not |
| 25535 (M228)-verify-R | CTCGACTTCTTGCGCGGG |  |
| 25570 (M228)-verify-F | ATTTCGGCGTGCGGATTC | To verify the mutant Δ25570 is successfully constructed or not |
| 25570 (M228)-verify-R | CACCTGACAACGGCACAC |  |
| M228-16SrRNA-F | GTGAAATCCCCGGGCTCA | To detect the transcription level of 16srRNA in M228 |
| M228-16SrRNA-R | GTTTGCTCCCCACGCTTT |  |
| RT-*hopY1* (M228) -F | GGCTGACCCCAATCACGA | To detect the transcription level of *hopY1* in M228 |
| RT-*hopY1* (M228) -R | CAAACCTTGCCCCGTTCC |  |
| RT-*hopAK1* (M228) -F | AAGACCTCGACACCTGCC | To detect the transcription level of *hopAK1* in M228 |
| RT-*hopAK1* (M228) -R | GGCCTTGTCGTCCTCCAT |  |
| RT-*hrpZ1* (M228) -F | TCACTGCATCGCTCGACA | To detect the transcription level of *hopZ1* in M228 |
| RT-*hrpZ1* (M228) -R | ACTGGGCCTTGTTGTCGT |  |
| RT-*hrpK1* (M228) -F | AAAAGCGCACCCACCAAC | To detect the transcription level of *hrpk1* in M228 |
| RT-*hrpK1* (M228) -R | CGCTTGCACGTCAGGATC |  |
| RT-16350 (M228)-F | GTTTCGACCGCTTCAACG | To detect the transcription level of RS16350 in M228 |
| RT-16350 (M228)-R | TGCAACTCGAGATCCTGC |  |
| RT-02395 (M228)-F | TACCTGAGGACGAGCTGTTG | To detect the transcription level of RS02395 in M228 |
| RT-02395 (M228)-R | TCTTTCAGATTGATGCGGCG |  |
| RT-07360 (M228)-F | GATCTGCAAACCGTCGAGAC | To detect the transcription level of RS07360 in M228 |
| RT-07360 (M228)-R | CCGATCATTTCAGGCTTGGG |  |
| RT-29160 (M228)-F | TGTAGGCCGTGATGTTGAGT | To detect the transcription level of RS29160 in M228 |
| RT-29160 (M228)-R | TGGAATCGTTACTTTGCGCC |  |
| RT-27610 (M228)-F | TGGAGGACGACAAGATCACC | To detect the transcription level of RS27610 in M228 |
| RT-27610 (M228)-R | CTGGTGTCGTGCTTGATCAG |  |
| RT-25575 (M228)-F | TCAACCTGCCGTACATCACT | To detect the transcription level of RS25575 in M228 |
| RT-25575 (M228)-R | GCGCTGAACCAAGTCTTCAA |  |
| RT-27290 (M228)-F | TGCCCAAAATTCAGAACCCG | To detect the transcription level of RS27290 in M228 |
| RT-27290 (M228)-R | CTTGCCGAGTTTGCTGTTCT |  |
| RT-27605 (M228)-F | GAGATGGAACTGGCGGTCT | To detect the transcription level of RS27605 in M228 |
| RT-27605 (M228)-R | GGCCGGAATCTTCACGTTC |  |
| RT-01550 (M228)-F | GGACAAGGCGGGGTATGT | To detect the transcription level of RS01550 in M228 |
| RT-01550 (M228)-R | CGAAACTGAACACCGCCC |  |
| RT-05155 (M228)-F | TGGGCCTCTCGGATATGT | To detect the transcription level of RS05155 in M228 |
| RT-05155 (M228)-R | TAATCCTTGCGACGCTCC |  |
| RT-18405 (M228)-F | TCGACGAGTGGCTGATGA | To detect the transcription level of RS18405 in M228 |
| RT-18405 (M228)-R | AGACGTTCGATCAGGCCT |  |
| RT-21050 (M228)-F | CGCAAATCCGACAACCCT | To detect the transcription level of RS21050 in M228 |
| RT-21050 (M228)-R | GATGCTTGCGACCACCTT |  |
| RT-24920 (M228)-F | TGATCAAGGTCGGTGCTG | To detect the transcription level of RS24920 in M228 |
| RT-24920 (M228)-R | CGCCTTTCAGACCTTCGA |  |
| RT-25395 (M228)-F | TTCTGCAGCCTAGCCCTC | To detect the transcription level of RS25395 in M228 |
| RT-25395 (M228)-R | CACGTGCTTCTTCGACCG |  |
| RT-25535 (M228)-F | CTGTCCAAGCGTGCACTG | To detect the transcription level of RS25535 in M228 |
| RT-25535 (M228)-R | TGGTGACCATGTTCCGGG |  |
| RT-25570 (M228)-F | GAGCTTGAAGTGCCGACG | To detect the transcription level of RS25570 in M228 |
| RT-25570 (M228)-R | GCACATCAAGTCACCCGC |  |
| pHM1-TrpR2 (M228)-F1 (*Hind*III) | ATGACCATGATTACGCCAAGCTTATGGCGACTGCATTTTCA | Overexpress TrpR2 in M228 |
| pHM1-TrpR2 (M228)-R2 (*Hind*III) | GACCTGCAGGCATGCAAGCTTTCAGCTGTTCAGTACTTT | Overexpress TrpR2 in M228 |
| pHM1-TrpR2_D18A_-F1 (*Hind*III) | ACGCCATGGCGACTGCATTTTCACTGGCTCCACTCTTTCGTCATTCCGTGGGTTTCGCC | Overexpress *TrpR2* in M228 and the 18th amino acid residue mutated to alanine |
| pHM1-TrpR2_D18A_-R1 (*Hind*III) | GGCGAAACCCACGGAATGACGAAAGAGTGGAGCCAGTGAAAATGCAGTCGCCATGGCGT |  |
| pHM1-TrpR2_D18A_-F2 (*Hind*III) | CATTCCGTGGGTTTCGCC |  |
| pHM1-TrpR2_P37A_-R1 (*Hind*III) | AGCGTAACCGCTGGCTGT | Overexpress *TrpR2* in M228 and the 37th amino acid residue mutated to alanine |
| pHM1-TrpR2_P37A_-F2 (*Hind*III) | ACAGCCAGCGGTTACGCT |  |
| pHM1-TrpR2_G57A_-R1 (*Hind*III) | GGCTGCTGCAGCGATCAC | Oerexpressn *TrpR2* in M228 and the 57th amino acid residue mutated to alanine |
| pHM1-TrpR2_G57A_-F2 (*Hind*III) | GTGATCGCTGCAGCAGCC |  |
| pHM1-TrpR2_L72A_-R1 (*Hind*III) | CGCCACACCTTTTTCGAC | Overexpress *TrpR2* in M228 and the 72nd amino acid residue mutated to alanine |
| pHM1-TrpR2_L72A_-F2 (*Hind*III) | GTCGAAAAAGGTGTGGCG |  |
| pHM1-TrpR2_G76A_-R1 (*Hind*III) | GGCGATGACGGTCAGCAC | Overexpress *TrpR2* in M228 and the 76th amino acid residue mutated to alanine |
| pHM1-TrpR2_G76A_-F2 (*Hind*III) | GTGCTGACCGTCATCGCC |  |
| pHM1-TrpR2_F98A_-R1 (*Hind*III) | GGCTTCACGCCGGGCAAT | Overexpress *TrpR2* in M228 and the 98th amino acid residue mutated to alanine |
| pHM1-TrpR2_F98A_-F2 (*Hind*III) | ATTGCCCGGCGTGAAGCC |  |
| pHM1-TrpR2_L104A_-R1 (*Hind*III) | CGCCCTGAACGACAGCTT | Overexpress *TrpR2* in M228 and the 104th amino acid residue mutated to alanine |
| pHM1-TrpR2_L104A_-F2 (*Hind*III) | AAGCTGTCGTTCAGGGCG |  |
| pHM1-TrpR2_G118A_-R1 (*Hind*III) | GGCATGAGACAGATCGGC | Overexpress *TrpR2* in M228 and the 118th amino acid residue mutated to alanine |
| pHM1-TrpR2_G118A_-F2 (*Hind*III) | GCCGATCTGTCTCATGCC |  |
| pHM1-TrpR2_L120A_-R1+ (*Hind*III) | CGCCAGGCCATGAGACAG | Overexpress *TrpR2* in M228 and the 120th amino acid residue mutated to alanine |
| pHM1-TrpR2_L120A_-F2 (*Hind*III) | CTGTCTCATGGCCTGGCG |  |
| pBT-TrpR2(M228)-new-F (*BamH*I) | GGCGCGGCCGCATCGAATTCCATGGCGACTGCATTTTCA | To construct pBT carrying TrpR2 in M228 |
| pBT-TrpR2(M228)-new-R (*EcoR*I) | AATTAATTAACTCGAGGATCCTCAGCTGTTCAGTACTTT |  |
| pBT-TrpR2_R12A-S14A_ (M228)-F (*BamH*I) | GGCGCGGCCGCATCCATGGCGACTGCATTTTCACTGGCTCCACTCTTTGCTGCTGCCGT | To construct pBT carrying TrpR2 in M228 and the 12nd to 14th amino acid residue mutated to alanine |
| pBT-TrpR2_N30A-P37A_ (M228)-R1 | GTTGTAAGGAGCGGCAGCGGCGGCTGCCGCAGCTCGCGCCGC | To construct pBT carrying TrpR2 in M228 and the 30th to 37th amino acid residue mutated to alanine |
| pBT-TrpR2_N30A-P37A_ (M228)-F2 | GCGGCGCGAGCTGCGGCAGCCGCCGCTGCCGCTCCTTACAAC |  |
| pBT-TrpR2_R96A_ (M228)-R1 | CAGCTTGAATTCAGCCCGGGCAAT | To construct pBT carrying TrpR2 in M228 and the 96th amino acid residue mutated to alanine |
| pBT-TrpR2_R96A_ (M228)-F2 | ATTGCCCGGGCTGAATTCAAGCTG |  |
| pBT-TrpR2_R12A_ (M228)-F (*BamH*I) | GGCGCGGCCGCATCCATGGCGACTGCATTTTCACTGGCTCCACTCTTTGCTCATTCCGT | To construct pBT carrying TrpR2 in M228 and the 12nd amino acid residue mutated to alanine |
| pBT-TrpR2_H13A_ (M228)-F (*BamH*I) | GGCGCGGCCGCATCCATGGCGACTGCATTTTCACTGGCTCCACTCTTTCGTGCTTCCGT | To construct pBT carrying TrpR2 in M228 and the 13th amino acid residue mutated to alanine |
| pBT-TrpR2_S14A_ (M228)-F (*BamH*I) | GGCGCGGCCGCATCCATGGCGACTGCATTTTCACTGGCTCCACTCTTTCGTCATGCCGT | To construct pBT carrying TrpR2 in M228 and the 14th amino acid residue mutated to alanine |
| pRTG-HrpL_N43A to D48A_ (M228)-R1 | GAGAATGTCAGCCGCAGCAGCAGCGGCCATCAC | To construct pTRG carrying HrpL in M228 and the 43th to 48th amino acid residue mutated to alanine |
| pRTG-HrpL_N43A to D48A_ (M228)-F2 | GTGATGGCCGCTGCTGCTGCGGCTGACATTCTC |  |
| pRTG-HrpL_H86A_ (M228)-R1 | GCGGAAGGCATTGCGGATCAG | To construct pTRG carrying HrpL in M228 and the 86th amino acid residue mutated to alanine |
| pRTG-HrpL_H86A_ (M228)-F2 | CTGATCCGCAATGCCTTCCGC |  |
| pRTG-HrpL_L89A to M90A_ (M228)-R1 | CTGACGATACGCTGCGCGGAA | To construct pTRG carrying HrpL in M228 and the 89th to 90th amino acid residue mutated to alanine |
| pRTG-HrpL_L89A to M90A_ (M228)-F2 | TTCCGCGCAGCGTATCGTCAG |  |
| pRTG-HrpL_S144A_ (M228)-R1 | CTCCAGCGACACTTCCAG | To construct pTRG carrying HrpL in M228 and the 144th amino acid residue mutated to alanine |
| pRTG-HrpL_S144A_ (M228)-F2 | CTGGAAGTGTCGCTGGAG |  |
| pRTG-HrpL_D148A to Y151A_ (M228)-R1 | TTCCTGAGCAGCGGCGGCCATCTC | To construct pTRG carrying HrpL in M228 and the 148th to 151st amino acid residue mutated to alanine |
| pRTG-HrpL_D148A to Y151A_ (M228)-F2 | GAGATGGCCGCCGCTGCTCAGGAA |  |
| pRTG-HrpL_D148A_ (M228)-R1 | TCGCTGGAGATGGCCGGCAATTATCAGGAAACAGCG | To construct pTRG carrying HrpL in M228 and the 148th amino acid residue mutated to alanine |
| pRTG-HrpL_D148A_ (M228)-F2 | TCGCTGGAGATGGCCGGCAATTATCAGGAAACAGCG |  |
| pRTG-HrpL_G149A_ (M228)-R1 | CGCTGTTTCCTGATAATTGGCGTCCATCTCCAGCGA | To construct pTRG carrying HrpL in M228 and the 149th amino acid residue mutated to alanine |
| pRTG-HrpL_G149A_ (M228)-F2 | TCGCTGGAGATGGACGCCAATTATCAGGAAACAGCG |  |
| pRTG-HrpL_N150A_ (M228)-R1 | CGCTGTTTCCTGATAAGCGCCGTCCATCTCCAGCGA | To construct pTRG carrying HrpL in M228 and the 150th amino acid residue mutated to alanine |
| pRTG-HrpL_N150A_ (M228)-F2 | TCGCTGGAGATGGACGGCGCTTATCAGGAAACAGCG |  |
| pRTG-HrpL_Y151A_ (M228)-R1 | CGCTGTTTCCTGAGCATTGCCGTCCATCTCCAGCGA | To construct pTRG carrying HrpL in M228 and the 151st amino acid residue mutated to alanine |
| pRTG-HrpL_Y151A_ (M228)-F2 | TCGCTGGAGATGGACGGCAATGCTCAGGAAACAGCG |  |
| pHM1-TrpR2_R96A_-R1 | CAGCTTGAATTCAGCCCGGGCAATACC | To construct a vector that Overexpress TrpR2 in M228 and the 96th amino acid residue mutated to alanine |
| pHM1-TrpR2_R96A_-F2 | GGTATTGCCCGGGCTGAATTCAAGCTG |  |
| pHM1-TrpR2_S14A_-F (*Hind*III) | TGATTACGCCAAGCTTGATGGCGACTGCATTTTCACTGGCTCCACTCTTTCGTCATGCC | To construct a vector that Overexpress TrpR2 in M228 and the 14th amino acid residue mutated to alanine |
| pETDuet-HrpL (M228)-F (*EcoR*I) | tcatcaccacagccaggatccATGTTTCAGAAGATTGTGATCCTCG | To construct pETDuet carrying HrpL |
| pETDuet-HrpL (M228)-R (*BamH*I) | aggcgcgccgagctcgaattcTCAGGCGAACGGGTCGAT |  |
| pETDuet-TrpR2 (M228)-F (*Kpn*Ⅰ) | gcgatcgctgacgtcggtaccATGGCGACTGCATTTTCACTG | To construct pETDuet carrying HrpL and TrpR2 simultaneously |
| pETDuet-TrpR2 (M228)-R (*Xho*Ⅰ) | ggtttctttaccagactcgagTCAGCTGTTCAGTACTTTCTGAGTATTG |  |

**References:**

Addinall, Stephen G., Kenneth A. Johnson, Timothy Dafforn, Corinne Smith, Alison Rodger, Raul Paco Gomez, Katherine Sloan, Anne Blewett, David J. Scott, and David I. Roper. 2005. 'Expression, purification and crystallization of the cell-division protein YgfE fromEscherichia coli', *Acta Crystallographica Section F Structural Biology and Crystallization Communications*, 61: 305-07.

Belogurov, G. A., M. N. Vassylyeva, V. Svetlov, S. Klyuyev, N. V. Grishin, D. G. Vassylyev, and I. Artsimovitch. 2007. 'Structural basis for converting a general transcription factor into an operon-specific virulence regulator', *Mol Cell*, 26: 117-29.

Duan, K., C. Dammel, J. Stein, H. Rabin, and M. G. Surette. 2003. 'Modulation of Pseudomonas aeruginosa gene expression by host microflora through interspecies communication', *Molecuar Microbiology*, 50: 1477-91.

Innes, R. W., M. A. Hirose, and P. L. Kuempel. 1988. 'Induction of nitrogen-fixing nodules on clover requires only 32 kilobase pairs of DNA from the Rhizobium trifolii symbiosis plasmid', *Journal of bacteriology*, 170: 3793-802.

Liao, J., D. Shen, L. Lin, H. Chen, Y. Jin, S. H. Chou, X. Q. Yu, T. Li, and G. Qian. 2021. 'Bacterial quorum sensing quenching activity of Lysobacter leucyl aminopeptidase acts by interacting with autoinducer synthase', *Computational and Structural Biotechnology Journal*, 19: 6179-90.

Shao, X., M. Tan, Y. Xie, C. Yao, T. Wang, H. Huang, Y. Zhang, Y. Ding, J. Liu, L. Han, C. Hua, X. Wang, and X. Deng. 2021. 'Integrated regulatory network in Pseudomonas syringae reveals dynamics of virulence', *Cell Reports*, 34: 108920.

Wang, N., N. Han, R. Tian, J. Chen, X. Gao, Z. Wu, Y. Liu, and L. Huang. 2021. 'Role of the Type VI Secretion System in the Pathogenicity of Pseudomonas syringae pv. actinidiae, the Causative Agent of Kiwifruit Bacterial Canker', *Frontiers in Microbiology*, 12: 627785.

Xu, G., S. Han, C. Huo, K. H. Chin, S. H. Chou, M. Gomelsky, G. Qian, and F. Liu. 2018. 'Signaling specificity in the c-di-GMP-dependent network regulating antibiotic synthesis in Lysobacter', *Nucleic Acids Resarch*, 46: 9276-88.
